# Circulating miR-20b-5p and miR-330-3p are novel biomarkers for progression of atrial fibrillation: Intracardiac/extracardiac plasma sample analysis by small RNA sequencing

**DOI:** 10.1371/journal.pone.0283942

**Published:** 2023-04-04

**Authors:** Masahide Harada, Daisuke Okuzaki, Akemi Yamauchi, Shiho Ishikawa, Yoshihiro Nomura, Asuka Nishimura, Yuji Motoike, Masayuki Koshikawa, Keisuke Hitachi, Kunihiro Tsuchida, Kentaro Amano, Atsuo Maekawa, Yasushi Takagi, Eiichi Watanabe, Yukio Ozaki, Hideo Izawa

**Affiliations:** 1 Department of Cardiology, Fujita Health University, Toyoake, Japan; 2 Genome Information Research Center, Research Institute for Microbial Diseases, Osaka University, Suita, Japan; 3 Division for Therapies Against Intractable Diseases, Institute for Comprehensive Medical Science, Fujita Health University, Toyoake, Japan; 4 Department of Cardiovascular Surgery, Fujita Health University Hospital, Toyoake, Japan; Ohio State University, UNITED STATES

## Abstract

**Background:**

Circulating microRNAs (miRNAs, miR) have been considered as biomarkers reflecting the underlying pathophysiology in atrial fibrillation (AF). Nevertheless, miRNA expression in the peripheral blood samples might not reflect a cardiac phenomenon since most miRNAs are expressed in numerous organs. This study aimed to identify the cardiac-specific circulating miRNAs as biomarkers for AF.

**Methods:**

Plasma samples were obtained from a luminal coronary sinus catheter (CS, cardiac-specific samples) and femoral venous sheath (FV, peripheral samples) in patients with AF and paroxysmal supraventricular tachycardia (control, CTL) undergoing catheter ablation. The circulating miRNA profiles were analyzed by small RNA sequencing. Differently expressed miRNAs between AF and CTL were identified in each sample of the CS and FV; miRNAs exhibiting similar expression patterns in the CS and FV samples were selected as candidates for cardiac-specific biomarkers. The selected miRNAs were related to the outcome of catheter ablation of AF.

**Results:**

Small RNA sequencing detected 849 miRNAs. Among the top 30 most differently expressed miRNAs between AF and CTL, circulating hsa-miR-20b-5p, hsa-miR-330-3p, and hsa-miR-204-5p had a similar pattern in the CS and FV samples. Another set of peripheral blood samples was obtained from AF patients undergoing catheter ablation (n = 141). The expression of the miR-20b-5p and miR-330-3p, but not the miR-204-5p, negatively correlated with the echocardiographic left-atrial dimension and was decreased in patients with AF recurrence as compared to those without AF recurrence during a 1-year follow-up.

**Conclusion:**

Circulating miR-20b-5p and miR-330-3p can be cardiac-specific biomarkers for atrial remodeling progression and arrhythmia recurrence after catheter ablation in AF patients.

## Introduction

Atrial fibrillation (AF) is the most common sustained arrhythmia, associated with increased morbidity and mortality. The increasing prevalence turns this complex arrhythmia into a major health problem [1].

AF evolves from a paroxysmal to a persistent form with the progression of atrial remodeling [2]. Catheter ablation is recognized as a standard treatment of AF but its efficacy has been limited in patients who develop atrial remodeling progression, resulting in a rather high rate of AF recurrence [3]. Although the progression of AF can be assessed by laboratory tests, imaging examinations, and electrophysiological findings, reliable clinical biomarkers have yet to be identified.

MicroRNAs (miRNAs) are small (20–25 bp), single-stranded, non-coding RNAs, which post-transcriptionally regulate the gene/protein expression. MiRNAs are reportedly involved in the pathophysiological processes of atrial remodeling and therefore become novel therapeutic targets [4]. MiRNAs can be incorporated into microparticles and are released from the cells when the complex fuses with the plasma membrane, and thus can be observed in the circulating blood. Circulating miRNAs have been considered as attractive biomarkers reflecting an underlying pathophysiology in various diseases [5].

Nevertheless, miRNA expression in the peripheral blood samples might not reflect a cardiac phenomenon since most miRNAs are expressed in numerous organs. Therefore, identifying the specific miRNAs reflecting cardiac events is important for exploiting a miRNA-based AF assessment. Given that miRNAs are expressed in the myocardium and are secreted into the coronary blood circulation, the altered miRNA expression seen in peripheral blood samples would originate in the heart and therefore be observed specifically in coronary sinus (CS) blood samples.

This study performed small RNA sequencing in the CS blood samples and compared the miRNA expression profiles between sinus rhythm and AF patients; the findings in the CS samples were also confirmed in the femoral vein (FV) blood samples. The aim of this study was to explore the cardiac-specific circulating miRNAs as biomarkers for AF progression and recurrence after catheter ablation.

## Materials and methods

### Patient selection

This was a single-center prospective study. Patients undergoing catheter ablation of AF or supraventricular tachycardia (control, CTL) were eligible during the period from September 2016 to June 2019. The study was conducted in accordance with the Declaration of Helsinki, and the protocol was approved by the Bioethics Committee of Fujita Health University (HM20-507). Written informed consent was obtained from all patients undergoing catheter ablation before participation in the study. The baseline demographics/clinical information (AF type, past history, comorbidities, medications, etc.) was obtained, and the CHADS_2_ and CHA_2_DS_2_-VASc scores were calculated. AF type was categorized into two groups: paroxysmal AF (self-terminating within 7 days after onset) and persistent AF (lasting longer than 7 days after onset). Laboratory examinations (creatinine and brain natriuretic peptide, etc.) and transthoracic echocardiography (ejection fraction and left atrial dimension, etc.) were performed before the catheter ablation. Transesophageal echocardiography was performed one day before the procedure, and patients with a left atrial appendage thrombus detected by transesophageal echocardiography were excluded. Patients with a creatinine clearance (calculated by Cockcroft-Gault formula) <15 mL/min and those on hemodialysis were excluded from the study. Patients with mechanical valves were also excluded.

**Fig 1:**
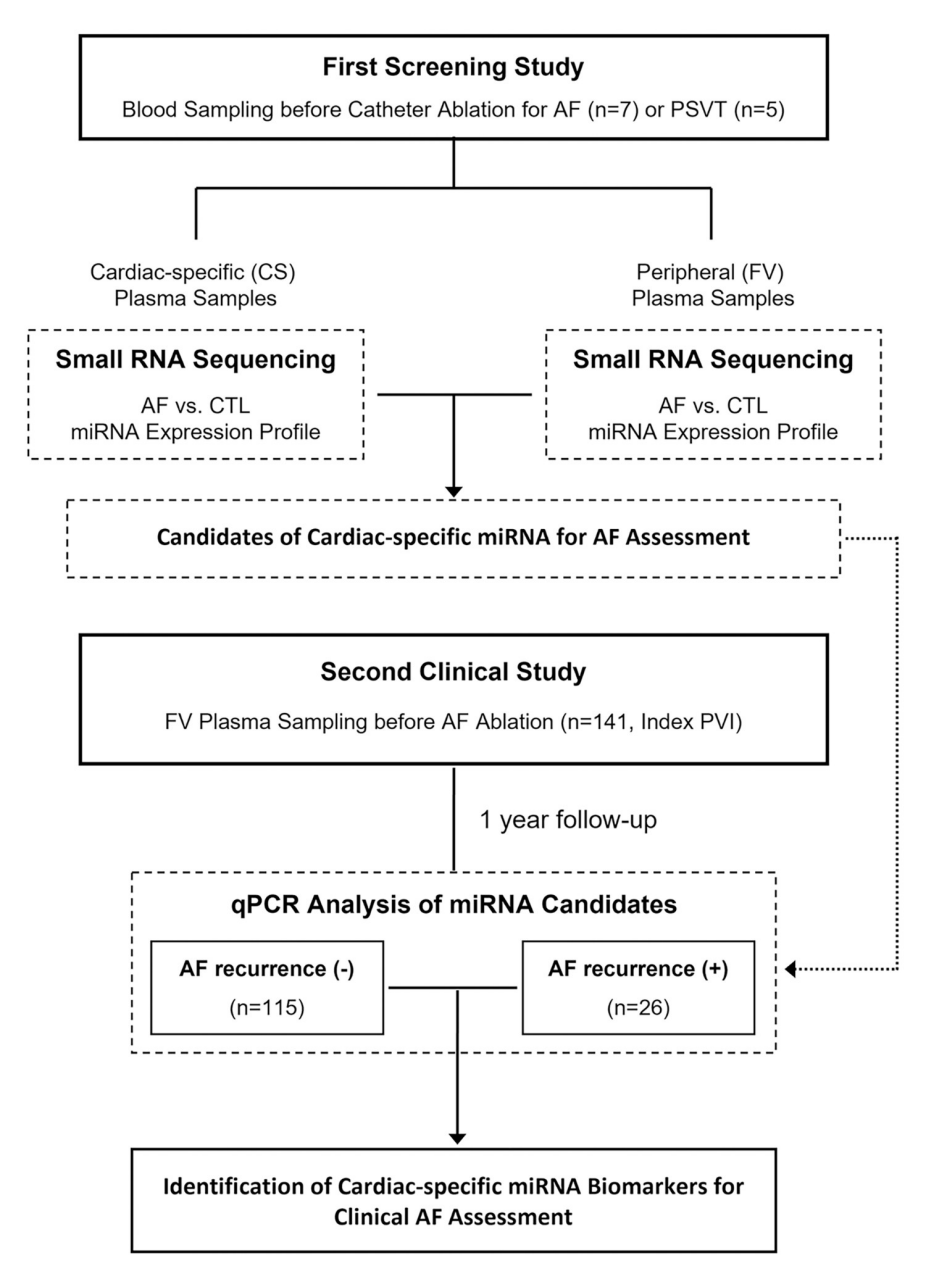
Schematic diagram of the study protocol. AF, atrial fibrillation; CS, coronary sinus; CTL, control; FV, femoral vein; PSVT, paroxysmal supraventricular tachycardia; PVI, pulmonary vein isolation.

This study protocol consisted of two parts: in the first screening study, we performed small RNA sequencing to find the candidate of cardiac-specific circulating miRNA associated with AF pathophysiology. In the second clinical study, we targeted the candidates of cardiac-specific miRNAs and assessed their availability as biomarkers using qPCR in the second clinical study (Fig 1).

### Blood sample collection

After puncturing the right FV and right internal jugular vein, the sheaths were inserted. An electrode catheter with an internal lumen was advanced to the CS via the sheath placed in the right internal jugular vein. Before the heparin administration prior to the trans-septal puncture, blood was simultaneously withdrawn from a right FV sheath and luminal CS electrode catheter; a 30 ml sample was obtained for the study purpose after discarding the first 10 ml of blood. When a blood sample could not be successfully withdrawn from the luminal CS catheter, typically due to an obstruction of the tip by the CS wall, the catheter was withdrawn until adequate blood flow was confirmed. However, the CS catheter was never withdrawn beyond the mid CS as visualized using the left anterior oblique fluoroscopic view. While being hemolyzed, the blood samples were not used for the miRNA analysis. The blood samples were centrifuged at 3500 rpm for 5 minutes at room temperature. The resulting plasma samples were transferred to cryogenic tubes and stored at −80°C until the analysis.

### Small RNA extraction and quantitative PCR

Small RNAs were extracted from 200μl plasma samples with a miRNeasy Serum/Plasma Kit (Qiagen) and from RA-appendage tissues with a miRNeasy Kit (Qiagen) in accordance with the manufacturer’s protocol. As no plasma housekeeping genes in the context of plasma miRNA experiments have been established and validated for normalization of the miRNA content, we chose to use a fixed volume of a synthetic *Caenorhabditis elegans* miR-39 (cel-miR-39, 20 fmol/sample, synthesized by Qiagen) per sample as a spiked-in control to normalize for individual RNA isolation-related variations. Twenty fmol cel-miR-39 were introduced to each 200μl plasma sample [6]. The RNA quality was assessed with a NanoDrop ND-1000 spectrophotometer (NanoDrop Technologies, Rockland, DE), and a fixed volume of diluted RNA (5 μL) was subjected to reverse transcription using the TaqMan microRNA Reverse Transcription kit (Applied Biosystems) according to the manufacturer’s protocol. Subsequently, 1.33 μL of the product was used to detect the miRNA-expression by quantitative PCR using miRNA-specific stem-loop primers (Applied Biosystems) for the corresponding microRNA. Quantitative PCR reactions were performed on the ABI Prism 7900HT using the following program: 10 minutes of pre-incubation at 95°C, 45 cycles of 15 seconds of denaturation at 95°C, and 60 seconds of elongation at 60°C. Fluorescence-signals were detected in triplicate. The amplification data were normalized to a spiked-in cel-miR-39 expression for the plasma samples and to U6 snRNA expression for the RA-appendage samples and was quantified using the 2DDCt method.

### Small RNA sequencing

The RNA sequencing was performed by BIKEN Biomics services (Osaka, Japan). Small RNAs were extracted from 200μl plasma samples with a miRNeasy Serum/Plasma Kit (Qiagen) in accordance with the manufacturer’s protocol. The small RNA libraries were prepared from 6 μl of the RNA sample using an NEBNext Multiplex Small RNA Library Prep Set for Illumina (NEB) according to the manufacturer’s instructions. The concentration and size distribution of the libraries was measured by a Qubit (Thremo Fisher Scientific) and LabChip GX Touch (Perkin Elmer) and the size selection was performed by electrophoresis with 2% E-Gel EX Agarose Gel (Thremo Fisher Scientific) to trim the primer-dimer products. Sequencing was performed on an Illumina MiSeq platform in a 50-base single-end mode. The sequencing reads were mapped to a human miRNA reference sequence and analyzed by using miRbase (http://ccb.jhu.edu/software/tophat/index.shtml) and CLC genomics workbench v9.5.3 software (Qiagen). After trimming under default parameter settings to retain only reads with lengths 15–25 bp, the annotated miRNAs were normalized by measuring endogenous cel-miR-39 that was spiked in all samples at the same concentration (5 fmol) and then calculated as read counts per million mapped reads (RPM). MiRNAs with read counts more than 10 were analyzed for further data analysis.

### Ablation procedure

In paroxysmal AF patients, pulmonary vein (PV) isolation was performed using either cryo-balloon ablation (single short freezing for 180 sec in each PV) or radiofrequency catheter ablation (RFCA) in the first session. RFCA was selected in patients with common PV or large PV ostia (>28mm) based on the left atrium (LA) and PV anatomy evaluated by cardiac-computed tomography imaging before the procedure. In persistent AF patients, only the RFCA-based PVI strategy was used for the first AF ablation and no additional linear ablation in the LA was performed; only cavotricuspid isthmus ablation was permitted if typical atrial flutter was documented.

The cryo-balloon ablation procedure was achieved using electro-anatomical mapping (EnSite NavX, Abbott, St. Paul, MN, USA) and fluoroscopic guidance to position the cryo-balloon catheter. In the RFCA procedure, the PVI was achieved using a focal “point-by-point” catheter approach, delivering radiofrequency energy to the cardiac tissue with irrigation tip catheters (THERMOCOOL SMARTTOUCH® SF, Biosense Webster, Diamond Bar, CA, USA [target contact force: 10-20g, RF time: 30–60 seconds, irrigation flow rate: 8 ml/min for ≤30W, 15 ml/min for >30W, power control mode], or FlexAbility^TM^, Abbott, St Paul, MN, USA [RF time: 30–60 sec, irrigation flow rate: 10 ml/min for <38°C, 13 ml/min for ≥38°C, temperature control mode]). The RFCA lesion sets encircled the PV antra using electro-anatomical mapping (CARTO3, Biosense Webster, Diamond Bar, CA, USA or EnSite NavX, Abbott, St. Paul, MN, USA) and fluoroscopy guidance. All procedures were performed under sinus rhythm; internal (3-35J) or external (50-200J) electrical cardioversion was performed by gradually increasing the shock intensity to restore sinus rhythm when AF was observed before/during the procedure.

### Follow-up

After catheter ablation, patients were followed up at the outpatient department in Fujita Health University at 1, 3, 6, 9, and 12 months. All patients were asked about their symptoms and underwent a 12-lead electrocardiogram. All patients discontinued antiarrhythmic drugs after a 3-month blanking period. Holter ECG monitoring was performed at the 6- and 12-month follow-ups. AF recurrence (AR) was defined as any atrial tachy-arrhythmias lasting more than 30 seconds after the blanking period.

### Statistical methods

Continuous variables, represented as the mean ± standard deviation, were compared using unpaired t-tests for parametric data and the Mann-Whitney test for nonparametric data. Categorical data, expressed as frequencies and percentages, were compared using a Fisher’s exact test. A linear regression model was used to examine the correlation between the plasma expression levels of miRNAs and the LAD on echocardiography.

All tests were 2-sided, and a p value <0.05 was considered statistically significant. The statistical analyses were performed using JMP11 software (SAS Institute, Cary, NC, USA).

## Results

Small RNA sequencing was performed to screen the circulating miRNA profiles. Twelve patients undergoing catheter ablation were eligible (CTL, n = 5 and persistent AF, n = 7). Patient characteristics are shown in Table 1. AF patients were older than the CTL, but the difference did not reach statistical significance. AF patients had a significantly larger LAD than the CTL. The serum NT-ProBNP levels were higher in the AF patients than in the CTL. The EF and LV diastolic dimension were unchanged between the two groups.

**Table 1:** Patient characteristics in the CTL and AF patients for the small RNA sequencing.

	CTL (n = 5)	AF (n = 7)	P value
Age, y	52±15	61±9	0.212
Male, n (%)	3 (60)	5 (71)	
BMI, kg/m^2^	23.2±3.6	25.1±2.5	0.307
CHADS_2_ score, pts	0.2±0.44	0.9±0.9	0.167
CHA_2_DS_2_-VASc score, pts	0.8±0.8	1.7±1.3	0.188
Blood test			
Cr, mg/dl	0.70±0.13	0.93±0.23	0.077
CrCl, ml/min	82±25	97±26	0.349
NT-ProBNP, pg/ml	60±44	449±333	0.028
HbA1c, %	5.6±0.2	5.6±0.3	0.896
Echocardiography			
LVDd, mm	44±3	49±5	0.058
LVDs, mm	28±3	37±7	0.011
EF, %	59±2	51±11	0.112
LAD, mm	32±4	44±3	0.013

BMI: body mass index, Cr: creatinine, CrCl: creatinine clearance, EF: ejection fraction, LAD: left atrial diameter, LVDd/Ds: left ventricular diastolic/systolic dimension.

We first analyzed the miRNA expression profile in the CS samples and compared that between the AF and CTL patients. Small RNA sequencing detected 849 circulating miRNAs. The top 30 of the differently expressed miRNAs in the CS sample are shown in Table 2. The functional role of the miR-27b-5p and miR-133a-3p in the pathophysiology of AF has been reported in previous experimental studies [7, 8].

**Table 2:** Differently expressed miRNAs between the CTL and AF patients in the CS samples.

	miRNA	Sequence	FC	P-value
1	hsa-miR-133a-3p	UUUGGUCCCCUUCAACCAGCUG	-19.556	<0.001
2	hsa-miR-20b-5p	CAAAGUGCUCAUAGUGCAGGUAG	-7.646	0.015
3	hsa-miR-937-3p	AUCCGCGCUCUGACUCUCUGCC	-7.498	0.024
4	hsa-miR-141-3p	UAACACUGUCUGGUAAAGAUGG	3.805	0.028
5	hsa-miR-30b-3p	CUGGGAGGUGGAUGUUUACUUC	-7.887	0.033
6	hsa-miR-27b-5p	AGAGCUUAGCUGAUUGGUGAAC	7.703	0.034
7	hsa-miR-92a-1-5p	AGGUUGGGAUCGGUUGCAAUGCU	-6.551	0.039
8	hsa-miR-1260b	AUCCCACCACUGCCACCAU	-11.356	0.039
9	hsa-miR-363-5p	CGGGUGGAUCACGAUGCAAUUU	-8.367	0.043
10	hsa-miR-3150a-5p	CAACCUCGACGAUCUCCUCAGC	-8.733	0.044
11	hsa-miR-24-2-5p	UGCCUACUGAGCUGAAACACAG	-5.726	0.044
12	hsa-miR-889-3p	UUAAUAUCGGACAACCAUUGU	-10.146	0.047
13	hsa-miR-191-3p	GCUGCGCUUGGAUUUCGUCCCC	-4.957	0.050
14	hsa-miR-329-3p	AACACACCUGGUUAACCUCUUU	-11.306	0.063
15	hsa-miR-204-5p	UUCCCUUUGUCAUCCUAUGCCU	-5.907	0.065
16	hsa-miR-378c	ACUGGACUUGGAGUCAGAAGAGUGG	-5.392	0.081
17	hsa-miR-664a-3p	UAUUCAUUUAUCCCCAGCCUACA	-6.148	0.081
18	hsa-miR-197-3p	UUCACCACCUUCUCCACCCAGC	2.554	0.081
19	hsa-miR-330-3p	GCAAAGCACACGGCCUGCAGAGA	-3.966	0.082
20	hsa-miR-30c-1-3p	CUGGGAGAGGGUUGUUUACUCC	-6.193	0.082
21	hsa-miR-370-3p	GCCUGCUGGGGUGGAACCUGGU	-6.790	0.083
22	hsa-miR-3177-3p	UGCACGGCACUGGGGACACGU	-7.210	0.086
23	hsa-miR-1343-3p	CUCCUGGGGCCCGCACUCUCGC	-8.052	0.088
24	hsa-miR-106a-5p	AAAAGUGCUUACAGUGCAGGUAG	-4.723	0.093
25	hsa-miR-144-3p	UACAGUAUAGAUGAUGUACU	-5.426	0.095
26	hsa-miR-193b-5p	CGGGGUUUUGAGGGCGAGAUGA	8.609	0.101
27	hsa-miR-6786-3p	UGACGCCCCUUCUGAUUCUGCCU	-3.504	0.103
28	hsa-miR-324-3p	CCCACUGCCCCAGGUGCUGCUGG	-5.426	0.104
29	hsa-miR-181c-3p	AACCAUCGACCGUUGAGUGGAC	-5.297	0.105
30	hsa-miR-330-5p	UCUCUGGGCCUGUGUCUUAGGC	-5.697	0.107

FC, fold change.

**Fig 2:**
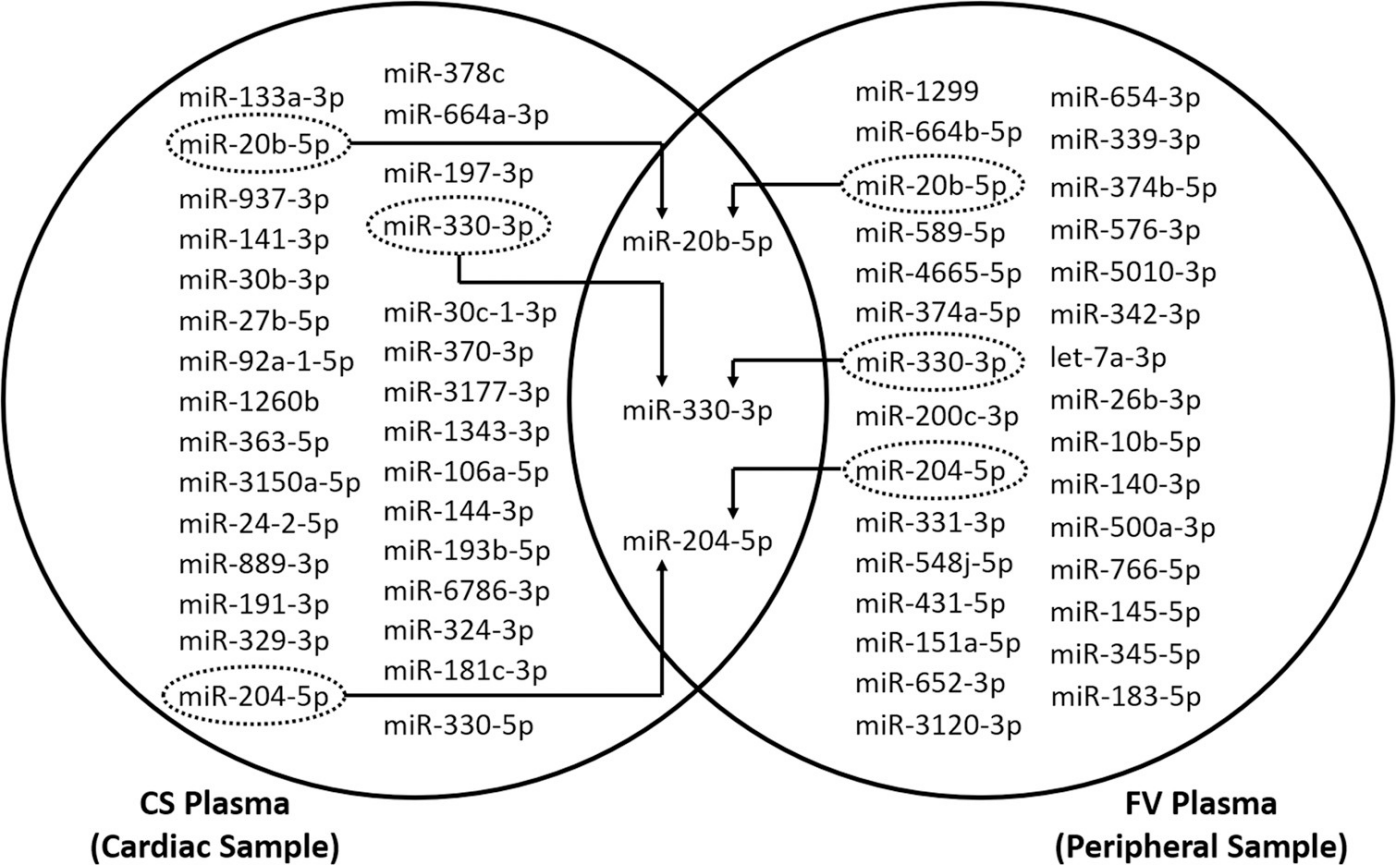
Top 30 differently expressed miRNAs between the CTL and AF patients in the CS and FV samples. CTL, control; CS, coronary sinus; FV, femoral vein; AF, atrial fibrillation.

Similarly, we analyzed the miRNA expression profile in the FV samples and compared them between the AF and CTL patients. The top 30 differently expressed miRNAs in the FV samples are shown in Table 3. We looked for specific miRNAs, which were simultaneously dysregulated in both the CS and FV samples: miR-20b-5p, miR-204-5p, and miR-330-3p, were identified (Fig 2). The miRNA expression difference between the AF and CTL patients was detectable in the both the CS and FV blood, and therefore was considered as a candidate for the cardiac-specific miRNA biomarkers.

**Table 3:** Differently expressed miRNAs between the CTL and AF patients in the FV samples.

	miRNA	Sequence	FC	P-value
1	hsa-miR-1299	UUCUGGAAUUCUGUGUGAGGGA	-17.837	0.001
2	hsa-miR-664b-5p	UGGGCUAAGGGAGAUGAUUGGGUA	-14.650	0.003
3	hsa-miR-20b-5p	CAAAGUGCUCAUAGUGCAGGUAG	-12.323	0.015
4	hsa-miR-589-5p	UGAGAACCACGUCUGCUCUGAG	-16.461	0.015
5	hsa-miR-4665-5p	CUGGGGGACGCGUGAGCGCGAGC	-9.877	0.019
6	hsa-miR-374a-5p	UUAUAAUACAACCUGAUAAGUG	-20.900	0.025
7	hsa-miR-330-3p	GCAAAGCACACGGCCUGCAGAGA	-7.661	0.028
8	hsa-miR-200c-3p	UAAUACUGCCGGGUAAUGAUGGA	-24.759	0.030
9	hsa-miR-204-5p	UUCCCUUUGUCAUCCUAUGCCU	-11.020	0.031
10	hsa-miR-331-3p	GCCCCUGGGCCUAUCCUAGAA	-16.777	0.033
11	hsa-miR-548j-5p	AAAAGUAAUUGCGGUCUUUGGU	-13.170	0.034
12	hsa-miR-431-5p	UGUCUUGCAGGCCGUCAUGCA	-18.582	0.034
13	hsa-miR-151a-5p	UCGAGGAGCUCACAGUCUAGU	-6.104	0.038
14	hsa-miR-652-3p	AAUGGCGCCACUAGGGUUGUG	-9.540	0.041
15	hsa-miR-3120-3p	CACAGCAAGUGUAGACAGGCA	-10.712	0.047
16	hsa-miR-654-3p	UAUGUCUGCUGACCAUCACCUU	-10.832	0.049
17	hsa-miR-339-3p	UCCCUGUCCUCCAGGAGCUCACG	-13.263	0.050
18	hsa-miR-374b-5p	AUAUAAUACAACCUGCUAAGUG	-10.630	0.054
19	hsa-miR-576-3p	AAGAUGUGGAAAAAUUGGAAUC	-5.149	0.057
20	hsa-miR-5010-3p	UUUUGUGUCUCCCAUUCCCCAG	-13.377	0.060
21	hsa-miR-342-3p	UCUCACACAGAAAUCGCACCCGU	-6.400	0.061
22	hsa-let-7a-3p	CUAUACAAUCUACUGUCUUUC	-7.358	0.065
23	hsa-miR-26b-3p	CCUGUUCUCCAUUACUUGGCU	-9.613	0.066
24	hsa-miR-10b-5p	UACCCUGUAGAACCGAAUUUGUG	2.639	0.068
25	hsa-miR-140-3p	UACCACAGGGUAGAACCACGG	-7.023	0.068
26	hsa-miR-500a-3p	AUGCACCUGGGCAAGGAUUCUG	-6.684	0.069
27	hsa-miR-766-5p	AGGAGGAAUUGGUGCUGGUCUU	-4.597	0.072
28	hsa-miR-145-5p	GUCCAGUUUUCCCAGGAAUCCCU	-6.673	0.073
29	hsa-miR-345-5p	GCUGACUCCUAGUCCAGGGCUC	-5.761	0.073
30	hsa-miR-183-5p	UAUGGCACUGGUAGAAUUCACU	3.015	0.075

FC, fold change.

**Fig 3:**
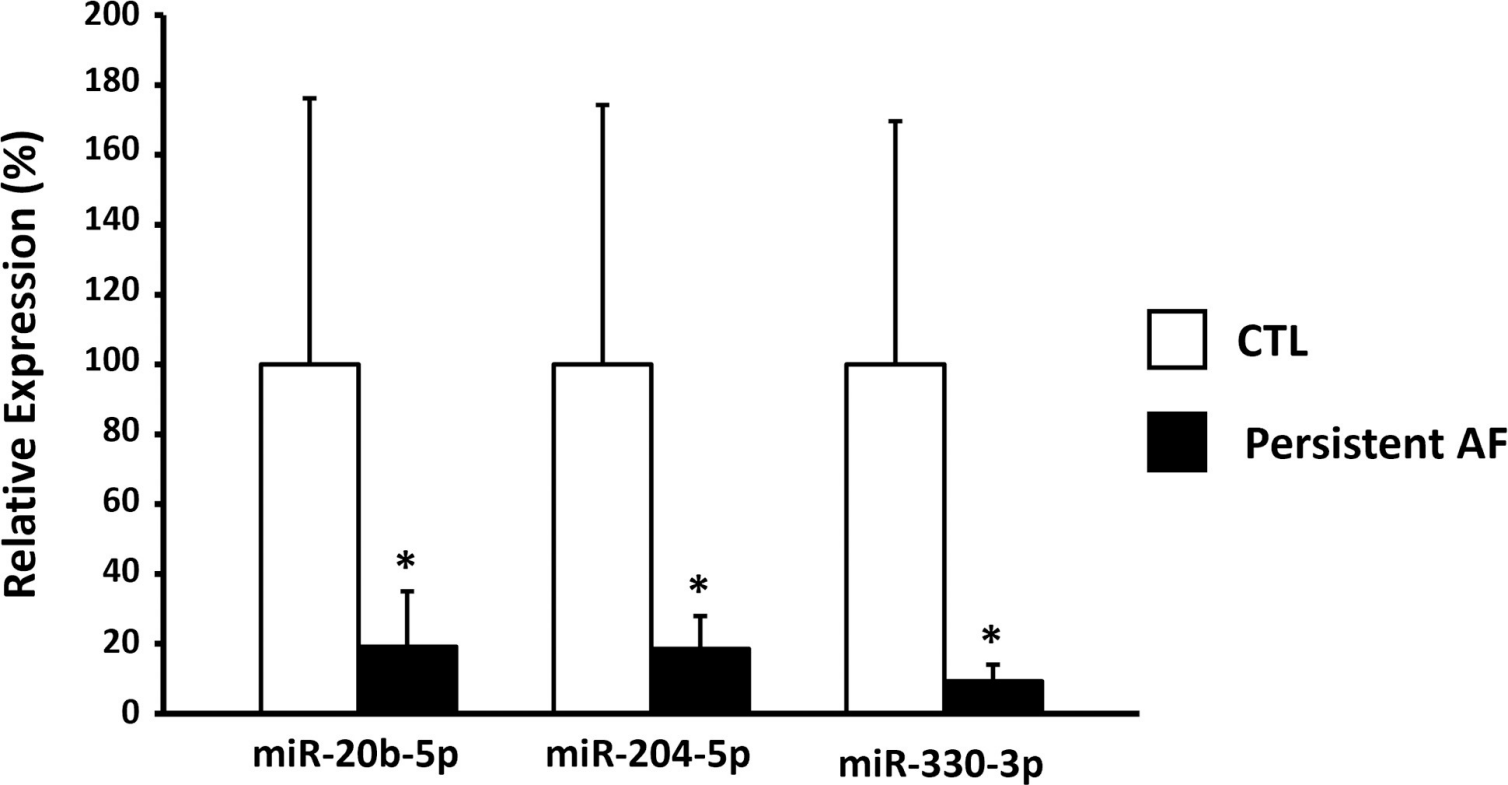
Mean±SD circulating miR-20b-5p, miR-204-5p, and miR-330-3p expression in the FV samples measured by qPCR. The open column indicates CTL and the solid column AF. CTL, control; AF, atrial fibrillation. *p<0.05.

The expression levels of the selected circulating miRNAs were validated by qPCR in the FV samples. AF patients had a significantly lower expression of miR-20b-5p, miR-204-5p, and miR-330-3p than that in the CTL in the FV samples (Fig 3), which was consistent with the results of the RNA sequencing.

**Fig 4:**
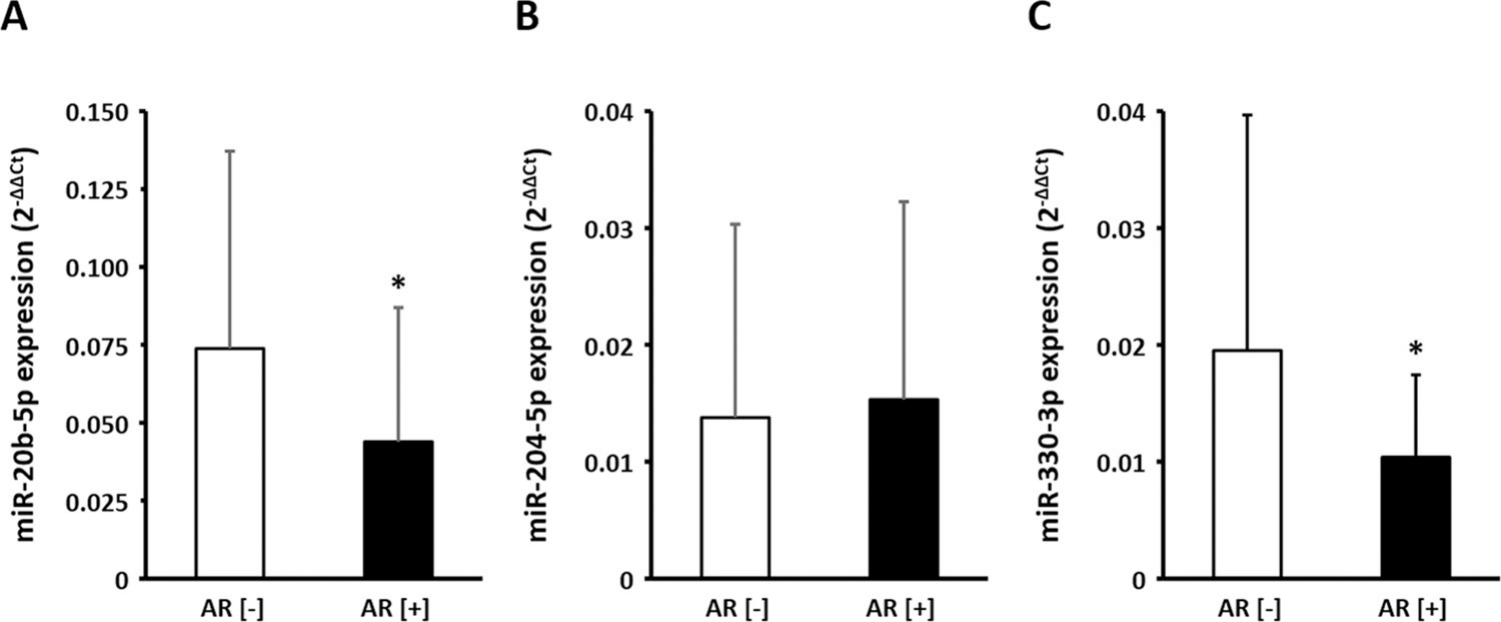
Mean±SD miR-20b-5p, miR-204-5p, and miR-330-3p expression in AF patients with and without AR after catheter ablation. AR, atrial tachy-arrhythmia recurrence. *p<0.05.

**Fig 5:**
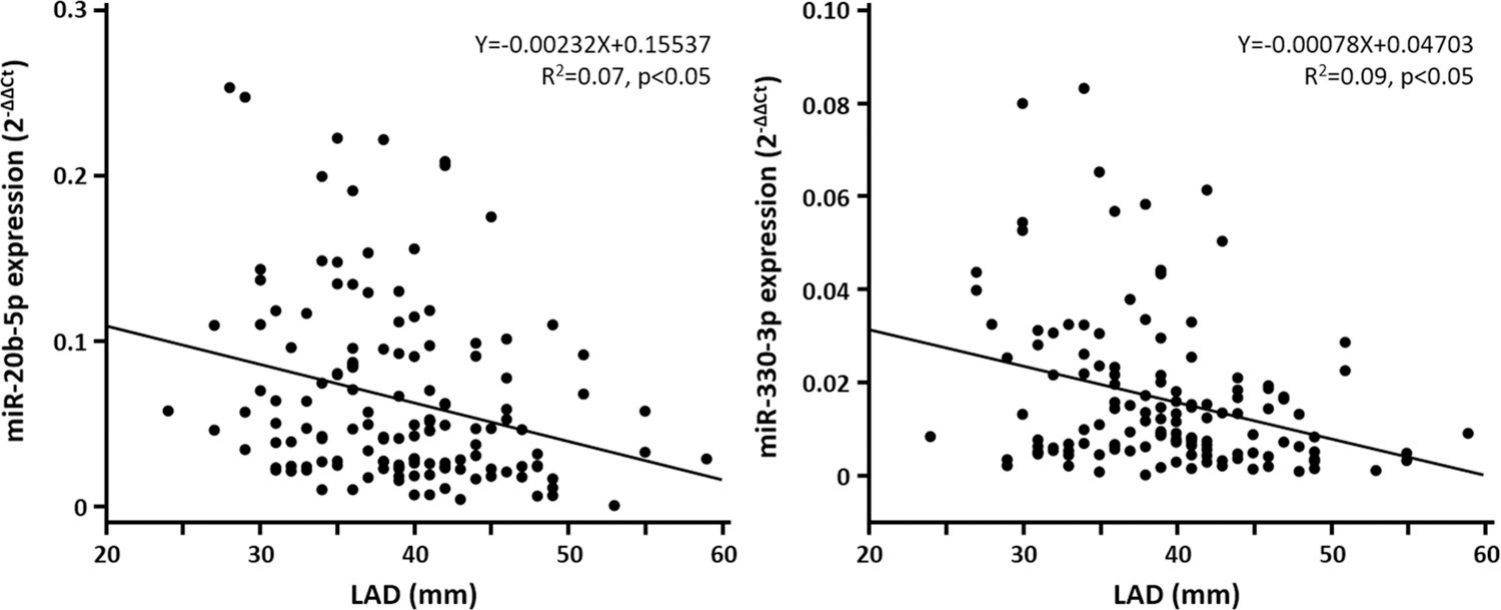
Relationship between the miR-20b-5p/miR-330-3p and LAD. LAD, left-atrial diameter.

In AF patients who underwent catheter ablation, we examined whether the miR-20b-5p, miR-204-5p, and miR-330-3p expression differed between the patients with and without AF recurrence (AR) one year after the catheter ablation. The plasma FV samples obtained at the time of the AF ablation were stored in a -80°C freezer in 141 AF patients. The patient characteristics are shown in Table 4. Twenty-six patients had AR during the 1-year follow-up whereas one-hundred fifteen patients did not. Patients with AR had a significantly larger left-atrial diameter (LAD) on echocardiography than those without AR (Table 4). The plasma expression of miR-20b-5p and miR-330-3p decreased in patients with AR as compared to those without AR. On the other hand, there were no significant differences in the miR-204-5p expression between patients with and without AR (Fig 4). A linear regression analysis demonstrated that the plasma expression of miR-20b-5p and miR-330-3p negatively correlated with the left atrial dimension (Fig 5).

**Table 4:** Patient characteristics of AF patients with and without AR.

	Total (n = 141)	AF patients without AR (n = 115)	AF patients with AR (n = 26)	p value
Age, y/o	66±11	66±11	67±11	0.796
Female, n (%)	38 (27)	30 (26)	8 (31)	0.631
BMI, kg/m^2^	24.1±3.9	24.0±3.9	24.5±3.7	0.560
AF type, persistent, n (%)	55 (39)	41 (36)	14 (54)	0.089
CHADS₂ sore, pts	1.3±1.2	1.3±1.2	1.5±1.1	0.295
CHA₂DS₂-VASc sore, pts	2.2±1.5	2.2±1.6	2.5±1.2	0.300
CHF, n (%)	40 (28)	26 (23)	14 (54)	0.002
HT, n (%)	71 (50)	60 (52)	11 (42)	0.363
DM, n (%)	28 (20)	23 (20)	5 (19)	0.929
Stroke/TIA, n (%)	8 (6)	6 (5)	2 (8))	0.635
Vascular disease, n (%)	2 (1)	2 (2)	0 (0)	0.365
Laboratory data				
Cr, mg/dl	0.85±0.26	0.84±0.23	0.89±0.38	0.806
CrCl, ml/min	82±30	83±29	81±31	0.390
NT-proBNP, pg/ml	516±893	431±703	887±1427	0.039
Echocardiography				
LVDd, mm	47±6	46±6	49±6	0.102
LVDs, mm	32±7	31±7	34±8	0.079
LVEF, %	57.4±8.7	58.1±7.8	54.3±11.7	0.046
LAD, mm	38.8±6.5	38.0±6.2	42.7±6.7	<0.001
Medication				
ACEI/ARB, n (%)	49 (35)	42 (37)	7 (27)	0.345
β-blocker, n (%)	64 (45)	51 (44)	13 (50)	0.602
Diuretic, n (%)	26 (19)	18 (16)	8 (31)	0.092
AAD, n (%)	20 (14)	16 (14)	4 (15)	0.860

AAD: anti-arrhythmic drug, ACEI/ARB: angiotensin converting enzyme inhibitor/angiotensin 2 receptor blocker, AR: atrial fibrillation/atrial tachycardia recurrence, BMI: body mass index, CHF: congestive heart failure, Cr: creatinine, CrCl: creatinine clearance, DM: diabetes mellitus, HT: hypertension, LAD: left-atrial diameter, LVEF: left-ventricular ejection fraction, LVDd/s: left-ventricular diastolic/systolic diameter, NT-proBNP: N-terminal pro-brain natriuretic peptide, AF: atrial fibrillation, TIA: Transient ischemic attack.

## Discussion

The major findings of this study were as follows. Small RNA sequencing could detect a wide range of circulating miRNAs in the CS and FV plasma samples in the AF patients undergoing catheter ablation. Among the detected circulating miRNAs, miR-20b-5p, miR-330-3p, and miR-204-5p, they exhibited similar expression differences between the AF and CTL patients for the both CS and FV samples. In catheter ablation for AF, patients with AR had a significantly larger baseline LAD than those without AR; the plasma expression levels of miR-20b-5p and miR-330-3p were decreased at the time of the catheter ablation in patients with AR as compared to those without AR, and negatively correlated with LAD. Therefore, circulating miR-20b-5p and miR-330-3p can be utilized as cardiac-specific miRNA biomarkers for atrial remodeling progression and AR after catheter ablation in AF patients.

Because of the small size and low abundance of miRNAs in body fluids, miRNA expression profiling is technically a challenge; next-generation sequencing (NGS) has recently become the preferred method for small RNA profiling [9]. When compared with standard quantitative real-time PCR, the NGS platforms are the most robust for a comprehensive expression profiling of miRNAs and offer the greatest detection sensitivity, largest dynamic range of detection, and highest accuracy [10]. Circulating miRNAs are considered as useful biomarkers for evaluating AF occurrence, progression, and recurrence after catheter ablation, and numerous studies have proposed various microRNAs as candidates. However, studies that used an NGS technology to detect circulating microRNAs for AF evaluation are largely limited.

A previous study by Kiliszek et al. performed small RNA sequencing in peripheral serum samples from AF patients undergoing catheter ablation [11]. They demonstrated 34 differentially expressed miRNAs in patients with and without AF recurrence. Actually, some of the differentially expressed miRNAs in the previous study were detected here as differently expressed miRNAs in the FV samples between the CTL and AF patients (hsa-miR-1299, hsa-miR-145-5p, and has-miR-183-5p, etc.). However, in the CS samples, we did not observe a similar gene expression difference between the CTL and AF patients, suggesting a non-cardiac origin of those miRNAs. The strongly different signals of the circulating miRNAs likely provided insight into the pathophysiology of AF but may also have reflected the response to AF in non-cardiac organs.

McManus et al. examined the plasma expression levels of miRNAs in the peripheral blood samples obtained from patients with and without AF [12]. They focused on 86 miRNAs expressed in the heart or associated with processes implicated in the pathogenesis of atrial remodeling or AF. The plasma levels of miR-21 (regulatory miRNA in fibrosis formation) and miR-150 (inflammation related miRNA) were lower in the patients with AF than in those without AF. In this study, the expression levels of miR-21 and miR-150 also differed between the CTL and AF patients for the FV samples, which was consistent with the previous study. However, the expression difference in the miR-21 and miR-150 between the CTL and AF patients was relatively small as compared to the top listed differently expressed miRNAs in this study.

There are several pathophysiological processes in atrial remodeling of AF, including electrical remodeling and fibrosis formation. Various miRNAs have been implicated in the pathophysiology [2, 4]. The previous experimental studies have demonstrated that miR-1, miR-26, and miR-328 regulate the gene encoding connexin 43, *I*_K1_, and *I*_CaL_ [13–15]. MiR-21, miR-26, miR-29, miR-133, and miR-590 have reportedly contributed to atrial fibrosis [8, 16–18]. AF itself and/or underlying diseases comorbid with AF (e.g., hypertension and heart failure) cause dysregulation of the miRNA, promoting AF and atrial remodeling. These functionally known miRNAs are likely secreted from the cells into the blood circulation, and thus have been examined to explore miRNA-based clinical biomarkers. However, circulating miRNAs do not always reflect the miRNA tissue levels. One possible explanation could be the retention of cellular miRNA at the expense of miRNA secretion into the circulation. It may be intriguing to speculate that the uptake by affected cells of circulating regulatory miRNAs to restore intracellular levels might contribute to the difference between the tissue and plasma levels.

There are inconsistent levels of expression of circulating miRNAs among the previous studies. The specificity and origin of the miRNAs in the blood circulation are largely unknown in these studies. To the best of our knowledge, this is the first clinical study to assess the source of circulating miRNAs in patients with AF. For this purpose, we examined the plasma miRNA expression difference between patients with and without AF in each sample from the FV and CS. Actually, this experimental approach has been reported to explore cardiac-specific biochemical markers of fibrosis and inflammation [19, 20]. In general, miRNAs can be secreted into the bloodstream from cells of numerous organs, including the liver, kidney, and brain. Regardless of the mechanism underlying the concentration pattern of miRNAs, it could be speculated that the differently expressed miRNAs between patients with and without AF in the CS samples are associated with a change in the miRNA amount in the cardiac tissue, likely representing the intracardiac phenomenon. The disparity in the levels of miRNA expression between the FV and CS suggests that the miRNAs may not reflect a cardiac-specific phenomenon but another non-cardiac process associated with AF.

In a previous study by Zhu S et al., miR-20b is implicated in the apoptosis, differentiation, and mitochondrial function of in in-vitro experiments using a cell line that can develop into cardiac muscle [21]. Mukhopadhyay et al. reported that miR-20b was significantly decreased in rat hearts subjected to ischemia/reperfusion. Pre-treatment of resveratrol and longevinex, components of red wine, suppress ischemia/reperfusion injury in the heart, likely via restoration of the miR-20b expression; pretreatment with antagomir-20b cancels the cardio-protective effects of resveratrol and longevinex [22]. MiR-20b may be responsible for heart development, ischemia/reperfusion injury, and cardiac hypertrophy [21–23], but the precise role of miR-20b in the AF pathophysiology has not yet been reported. Mir-330-3p has been reported to act as an oncogene in a wide variety of cancers including esophageal cancer [24] and breast cancer [25]. Regarding cardiovascular diseases, Zheng et al. demonstrated that miR-330-3p plays a role in aortic valve calcification and is upregulated in stenotic leaflets especially in patients with bicuspid aortic valve disease [26]. However, the information on the miR-330-3p in cardiovascular diseases also has been limited.

In this study, the plasma miR-20b-5p and miR-330-3p expression levels significantly decreased in the AF patients as compared to the CTL, suggesting that these miRNAs may contribute to AF progression. In AF ablation, the plasma miR-20b-5p and miR-330-3p expression levels decreased in patients with AR at 1 year after the procedure as compared to those without AR, and also negatively correlated with the LAD, an index of atrial structural remodeling. Atrial remodeling progression is one of the major causes of AF recurrence, and these miRNAs may be involved in the pathophysiology of the remodeling process.

There are some limitations of this study. This was a single-center study with a small number of patients; the statistical power was limited, and interpretations should be made with caution. Especially in the first screening study, we performed small RNA sequencing in 24 blood samples (each CS and FV from 5 controls and 7 AF patients) to find the candidate of cardiac-specific circulating miRNA associated with AF pathophysiology. Because of the limited number of NGS experiments based on our budget, we compared the miRNA profiles between persistent AF patients (who apparently have had atrial remodeling) and controls (who apparently have not had atrial remodeling), which might lead to selection bias. We targeted the candidates of cardiac-specific miRNAs and assessed their availability as biomarkers using qPCR in the second clinical study. However, NGS experiments should ideally be performed in all patients in both the first screening study and the second clinical study. There remains a discrepancy between the tissue levels and the plasma levels of the candidate miRNAs, and further impact on the disease progression. The correlation between the selected microRNA and LAD (Fig 4) is week weak and the causal relationship is still debatable. Our study involved only a 12-month follow-up and a longer prospective study will provide information on the clinical usefulness of miR-20b-5p and miR-330-3p. AF is a multi-factorial disease and other unmeasured factors might affect the miRNA expression. This study focused only on the AF progression and recurrence after AF treatment. Future studies should involve larger-scale AF populations and be performed in more detailed miRNA profiling to investigate the prognostic value of a single miRNA or a combination of multiple miRNAs for the occurrence, therapeutic response, and progression of AF. The miRNA-dependent mechanisms underlying atrial remodeling remain to be clarified and future experimental studies will be required to explore the role of miR-20b-5p and miR-330-3p in the AF pathophysiology.

## Conclusions

Small RNA sequencing of CS and FV samples revealed that miR-20b-5p and miR-330-3p are potential cardiac-specific miRNAs involved in AF progression; those can be utilized as clinical biomarkers for the development of atrial remodeling and AR after catheter ablation in AF patients.

## Supporting information

S1 File (PDF)
