# Supplementary material for: Circulating miR-20b-5p and miR-330-3p are novel biomarkers for progression of atrial fibrillation: Intracardiac/extracardiac plasma sample analysis by small RNA sequencing
Source: PLoS One. 2023 Apr 4;18(4):e0283942. doi: 10.1371/journal.pone.0283942 (PMC10072479; doi:10.1371/journal.pone.0283942)
Supplement: S1 File — (PDF) [file pone.0283942.s001.pdf]

**Data Set for Table 1**

| Pt | Diagnosis | Age (y/o) | Gender (male=1) | BMI | LVDd (mm) | LVDs (mm) | EF (%) | LAD (mm) | CRNN (mg/dl) | CrCl (ml/min) | HbA1c (%) | NT-ProBNP (pg/ml) | CHADS2 (point) | CHA2DS2-VASc (point) |
|---|---|---|---|---|---|---|---|---|---|---|---|---|---|---|
| #1 | Persistent AF | 66 | 1 | 24.6 | 46 | 30 | 57 | 42 | 0.80 | 100 | 5.5 | 200 | 0 | 1 |
| #2 | Persistent AF | 68 | 0 | 24.7 | 55 | 47 | 30 | 50 | 0.66 | 77 | 5.4 | 116 | 1 | 3 |
| #3 | Persistent AF | 49 | 1 | 28.4 | 45 | 31 | 56 | 47 | 0.91 | 114 | 5.8 | 362 | 1 | 1 |
| #4 | Persistent AF | 64 | 0 | 28.9 | 49 | 37 | 60 | 40 | 0.81 | 73 | 5.4 | 561 | 2 | 3 |
| #5 | Persistent AF | 47 | 1 | 23.6 | 55 | 45 | 43 | 44 | 0.92 | 104 | 5.7 | 221 | 0 | 0 |
| #6 | Persistent AF | 70 | 1 | 22.8 | 47 | 39 | 50 | 44 | 1.40 | 43 | 5.8 | 1078 | 2 | 3 |
| #7 | Persistent AF | 66 | 1 | 22.8 | 44 | 33 | 58 | 43 | 1.00 | 64 | 5.6 | 606 | 0 | 1 |
| **Pt** | **Diagnosis** | **Age (y/o)** | **Gender (male=1)** | **BMI** | **LVDd (mm)** | **LVDs (mm)** | **EF (%)** | **LAD (mm)** | **CRNN (mg/dl)** | **CrCl (ml/min)** | **HbA1c (%)** | **NT-ProBNP (pg/ml)** | **CHADS2 (point)** | **CHADS2-Vasc (point)** |
| #1 | AVNRT（CTL） | 56 | 0 | 27.3 | 43 | 24 | 60 | 37 | 0.58 | 115 | 6.2 | 67 | 1 | 2 |
| #2 | AVNRT（CTL） | 52 | 0 | 23.4 | 44 | 28 | 60 | 28 | 0.63 | 99 | 5.6 | 126 | 0 | 1 |
| #3 | AVRT（CTL） | 54 | 1 | 23.7 | 48 | 29 | 56 | 28 | 0.88 | 95 | 5.5 | 33 | 0 | 0 |
| #4 | AVRT（CTL） | 70 | 0 | 17.3 | 39 | 26 | 58 | 34 | 0.61 | 54 | 5.5 | 63 | 0 | 1 |
| #5 | AVRT（CTL） | 29 | 1 | 24.2 | 44 | 31 | 62 | 31 | 0.79 | 121 | 5.3 | 9 | 0 | 0 |

**Data Set for Table 2**

| | | | Control | | | | | Persistent | | | | | | | Control | Persistent |
|---|---|---|---|---|---|---|---|---|---|---|---|---|---|---|---|---|
| | | | CS | CS | CS | CS | CS | CS | CS | CS | CS | CS | CS | CS | CS | CS |
| | CS: Persitent vs Control | | #1 | #2 | #2 | #4 | #5 | #1 | #2 | #3 | #4 | #5 | #6 | #7 | #1-#5 | #1-#7 |
| ID | fold change | p-value | PS | PS | PS | PS | PS | PS | PS | PS | PS | PS | PS | PS | Average | Average |
| hsa-miR-133a-3p (miRBase21_mature_hsa) | -19.556 | 0.000 | | | | | | | | | | | | | 2.6213267 | -1.6681951 |
| hsa-miR-20b-5p (miRBase21_mature_hsa) | -7.646 | 0.015 | | | | | | | | | | | | | 2.2944674 | -0.64015853 |
| hsa-miR-937-3p (miRBase21_mature_hsa) | -7.498 | 0.024 | | | | | | | | | | | | | 1.9543221 | -0.9521409 |
| hsa-miR-141-3p (miRBase21_mature_hsa) | 3.805 | 0.028 | | | | | | | | | | | | | -0.29422677 | 1.6335484 |
| hsa-miR-30b-3p (miRBase21_mature_hsa) | -7.887 | 0.033 | | | | | | | | | | | | | 1.6047947 | -1.3746756 |
| hsa-miR-27b-5p (miRBase21_mature_hsa) | 7.703 | 0.034 | | | | | | | | | | | | | -2.1903512 | 0.75500196 |
| hsa-miR-92a-1-5p (miRBase21_mature_hsa) | -6.551 | 0.039 | | | | | | | | | | | | | 2.5457675 | -0.16600482 |
| hsa-miR-1260b (miRBase21_mature_hsa) | -11.356 | 0.039 | | | | | | | | | | | | | 1.8697735 | -1.6356425 |
| hsa-miR-363-5p (miRBase21_mature_hsa) | -8.367 | 0.043 | | | | | | | | | | | | | 1.7484956 | -1.3162426 |
| hsa-miR-3150a-5p (miRBase21_mature_hsa) | -8.733 | 0.044 | | | | | | | | | | | | | 2.2143052 | -0.91220313 |
| hsa-miR-24-2-5p (miRBase21_mature_hsa) | -5.726 | 0.044 | | | | | | | | | | | | | 2.3626254 | -0.15481208 |
| hsa-miR-889-3p (miRBase21_mature_hsa) | -10.146 | 0.047 | | | | | | | | | | | | | 2.1274986 | -1.2153863 |
| hsa-miR-191-3p (miRBase21_mature_hsa) | -4.957 | 0.050 | | | | | | | | | | | | | 2.2550986 | -0.05426826 |
| hsa-miR-329-3p (miRBase21_mature_hsa) | -11.306 | 0.063 | | | | | | | | | | | | | 2.3486211 | -1.1503754 |
| hsa-miR-204-5p (miRBase21_mature_hsa) | -5.907 | 0.065 | | | | | | | | | | | | | 2.450854 | -0.11161338 |
| hsa-miR-378c (miRBase21_mature_hsa) | -5.392 | 0.081 | | | | | | | | | | | | | 2.2710457 | -0.15970746 |
| hsa-miR-664a-3p (miRBase21_mature_hsa) | -6.148 | 0.081 | | | | | | | | | | | | | 1.6336969 | -0.986501 |
| hsa-miR-197-3p (miRBase21_mature_hsa) | 2.554 | 0.081 | | | | | | | | | | | | | -0.7830991 | 0.56939244 |
| hsa-miR-330-3p (miRBase21_mature_hsa) | -3.966 | 0.082 | | | | | | | | | | | | | 1.3645434 | -0.6230102 |
| hsa-miR-30c-1-3p (miRBase21_mature_hsa) | -6.193 | 0.082 | | | | | | | | | | | | | 1.5867858 | -1.0439674 |
| hsa-miR-370-3p (miRBase21_mature_hsa) | -6.790 | 0.083 | | | | | | | | | | | | | 1.3636509 | -1.3997979 |
| hsa-miR-3177-3p (miRBase21_mature_hsa) | -7.210 | 0.086 | | | | | | | | | | | | | 1.7050467 | -1.1450032 |
| hsa-miR-1343-3p (miRBase21_mature_hsa) | -8.052 | 0.088 | | | | | | | | | | | | | 1.7659082 | -1.2434698 |
| hsa-miR-106a-5p (miRBase21_mature_hsa) | -4.723 | 0.093 | | | | | | | | | | | | | 1.7536267 | -0.4862164 |
| hsa-miR-144-3p (miRBase21_mature_hsa) | -5.426 | 0.095 | | | | | | | | | | | | | 1.3605275 | -1.0794607 |
| hsa-miR-193b-5p (miRBase21_mature_hsa) | 8.609 | 0.101 | | | | | | | | | | | | | -2.8048835 | 0.30104455 |
| hsa-miR-6786-3p (miRBase21_mature_hsa) | -3.504 | 0.103 | | | | | | | | | | | | | 0.7894646 | -1.0197006 |
| hsa-miR-324-3p (miRBase21_mature_hsa) | -5.426 | 0.104 | | | | | | | | | | | | | 2.065076 | -0.374767 |
| hsa-miR-181c-3p (miRBase21_mature_hsa) | -5.297 | 0.105 | | | | | | | | | | | | | 1.9119402 | -0.4931708 |
| hsa-miR-330-5p (miRBase21_mature_hsa) | -5.697 | 0.107 | | | | | | | | | | | | | 2.3636246 | -0.14665268 |
| hsa-miR-1183 (miRBase21_mature_hsa) | -4.338 | 0.107 | | | | | | | | | | | | | 1.657965 | -0.45910293 |
| hsa-miR-4732-5p (miRBase21_mature_hsa) | -4.515 | 0.109 | | | | | | | | | | | | | 1.5366691 | -0.63791114 |
| hsa-miR-203a-3p (miRBase21_mature_hsa) | 2.916 | 0.112 | | | | | | | | | | | | | -0.5302466 | 1.0136156 |
| hsa-miR-23a-3p (miRBase21_mature_hsa) | -4.137 | 0.113 | | | | | | | | | | | | | 1.3366284 | -0.71204406 |
| hsa-miR-1248 (miRBase21_mature_hsa) | -3.051 | 0.114 | | | | | | | | | | | | | 0.93805534 | -0.67111 |
| hsa-miR-5189-3p (miRBase21_mature_hsa) | -3.051 | 0.114 | | | | | | | | | | | | | 1.4152675 | -0.1938978 |
| hsa-miR-618 (miRBase21_mature_hsa) | -3.051 | 0.114 | | | | | | | | | | | | | 1.4152675 | -0.1938978 |
| hsa-miR-6802-3p (miRBase21_mature_hsa) | -3.051 | 0.114 | | | | | | | | | | | | | 1.4152675 | -0.1938978 |
| hsa-miR-1908-5p (miRBase21_mature_hsa) | -3.807 | 0.115 | | | | | | | | | | | | | 0.75382763 | -1.174842 |
| hsa-miR-1273h-5p (miRBase21_mature_hsa) | -5.815 | 0.116 | | | | | | | | | | | | | 1.7444134 | -0.7954552 |
| hsa-miR-3161 (miRBase21_mature_hsa) | -4.289 | 0.118 | | | | | | | | | | | | | 1.6567093 | -0.44382784 |
| hsa-miR-4745-5p (miRBase21_mature_hsa) | -4.289 | 0.118 | | | | | | | | | | | | | 1.8532294 | -0.24730776 |
| hsa-miR-4732-3p (miRBase21_mature_hsa) | -4.838 | 0.121 | | | | | | | | | | | | | 1.2490672 | -1.0253711 |
| hsa-miR-148b-5p (miRBase21_mature_hsa) | 2.641 | 0.122 | | | | | | | | | | | | | -0.6275929 | 0.7734122 |
| hsa-miR-495-3p (miRBase21_mature_hsa) | -4.303 | 0.123 | | | | | | | | | | | | | 1.2934016 | -0.81182015 |
| hsa-miR-30e-5p (miRBase21_mature_hsa) | -2.955 | 0.125 | | | | | | | | | | | | | 1.3511331 | -0.21190473 |
| hsa-miR-654-3p (miRBase21_mature_hsa) | -3.632 | 0.128 | | | | | | | | | | | | | 1.8307383 | -0.030162303 |
| hsa-miR-3175 (miRBase21_mature_hsa) | 2.392 | 0.132 | | | | | | | | | | | | | -0.43311572 | 0.82503223 |
| hsa-miR-26b-3p (miRBase21_mature_hsa) | -6.544 | 0.137 | | | | | | | | | | | | | 1.250057 | -1.4600179 |
| hsa-miR-758-3p (miRBase21_mature_hsa) | 3.449 | 0.139 | | | | | | | | | | | | | -1.0575836 | 0.72866297 |
| hsa-miR-615-3p (miRBase21_mature_hsa) | 3.644 | 0.143 | | | | | | | | | | | | | -2.1230505 | -0.25744998 |
| hsa-miR-150-3p (miRBase21_mature_hsa) | -3.696 | 0.146 | | | | | | | | | | | | | 1.059598 | -0.82633394 |
| hsa-miR-494-3p (miRBase21_mature_hsa) | -4.276 | 0.151 | | | | | | | | | | | | | 2.390936 | 0.2945167 |
| hsa-miR-194-5p (miRBase21_mature_hsa) | -2.388 | 0.151 | | | | | | | | | | | | | 1.8935051 | 0.63788414 |
| hsa-miR-493-5p (miRBase21_mature_hsa) | -4.602 | 0.154 | | | | | | | | | | | | | 1.7075663 | -0.4946664 |
| hsa-miR-499a-5p (miRBase21_mature_hsa) | -4.959 | 0.161 | | | | | | | | | | | | | 0.79249024 | -1.5175847 |
| hsa-miR-548h-3p (miRBase21_mature_hsa) | -4.959 | 0.161 | | | | | | | | | | | | | 1.7030498 | -0.6070252 |
| hsa-miR-5010-3p (miRBase21_mature_hsa) | -6.468 | 0.163 | | | | | | | | | | | | | 1.5878494 | -1.1055796 |
| hsa-miR-1185-1-3p (miRBase21_mature_hsa) | 3.927 | 0.166 | | | | | | | | | | | | | -0.6888144 | 1.2846408 |
| hsa-miR-548j-5p (miRBase21_mature_hsa) | -3.592 | 0.167 | | | | | | | | | | | | | 1.4399843 | -0.4046432 |
| hsa-miR-4467 (miRBase21_mature_hsa) | -3.869 | 0.168 | | | | | | | | | | | | | 1.5166054 | -0.43521458 |
| hsa-miR-671-5p (miRBase21_mature_hsa) | -3.869 | 0.168 | | | | | | | | | | | | | 1.6204958 | -0.33132416 |
| hsa-miR-6857-3p (miRBase21_mature_hsa) | -3.869 | 0.168 | | | | | | | | | | | | | 1.6209416 | -0.33087823 |
| hsa-miR-181a-2-3p (miRBase21_mature_hsa) | -4.201 | 0.174 | | | | | | | | | | | | | 1.1045583 | -0.9660273 |
| hsa-miR-339-5p (miRBase21_mature_hsa) | -2.588 | 0.174 | | | | | | | | | | | | | 1.173838 | -0.19814959 |
| hsa-miR-337-3p (miRBase21_mature_hsa) | -3.457 | 0.175 | | | | | | | | | | | | | 1.4688778 | -0.32064232 |

| | | | | |
|---|---|---|---|---|
| hsa-miR-10a-3p (miRBase21_mature_hsa) | 2.555 | 0.175 | -0.22444305 | 1.1288216 |
| hsa-miR-5698 (miRBase21_mature_hsa) | 2.555 | 0.175 | -0.2209867 | 1.132278 |
| hsa-miR-6772-3p (miRBase21_mature_hsa) | -4.848 | 0.178 | 1.4961926 | -0.7811469 |
| hsa-miR-654-5p (miRBase21_mature_hsa) | -3.664 | 0.179 | 0.40620688 | -1.4670181 |
| hsa-miR-1306-5p (miRBase21_mature_hsa) | -4.503 | 0.189 | 1.4025931 | -0.76817584 |
| hsa-miR-340-5p (miRBase21_mature_hsa) | -2.730 | 0.193 | 0.5012281 | -0.9477412 |
| hsa-miR-122-5p (miRBase21_mature_hsa) | 3.595 | 0.193 | -1.2945867 | 0.5515425 |
| hsa-miR-200a-3p (miRBase21_mature_hsa) | 3.586 | 0.200 | -0.6710042 | 1.1715329 |
| hsa-miR-144-5p (miRBase21_mature_hsa) | -3.918 | 0.205 | 1.4525464 | -0.5176695 |
| hsa-miR-199b-3p (miRBase21_mature_hsa) | -2.609 | 0.208 | 0.844433 | -0.53893775 |
| hsa-miR-181a-5p (miRBase21_mature_hsa) | -2.874 | 0.216 | 0.8631958 | -0.6597648 |
| hsa-miR-339-3p (miRBase21_mature_hsa) | -2.778 | 0.217 | 0.9831742 | -0.49095753 |
| hsa-miR-379-5p (miRBase21_mature_hsa) | -5.222 | 0.218 | 0.8459112 | -1.5387241 |
| hsa-miR-30b-5p (miRBase21_mature_hsa) | -5.025 | 0.219 | 1.8006076 | -0.5284908 |
| hsa-miR-5584-5p (miRBase21_mature_hsa) | 3.695 | 0.220 | -0.7750598 | 1.1103522 |
| hsa-miR-548z (miRBase21_mature_hsa) | -4.736 | 0.227 | 2.23535 | -0.008426489 |
| hsa-miR-3188 (miRBase21_mature_hsa) | -3.203 | 0.229 | 0.72106713 | -0.95833 |
| hsa-miR-23b-5p (miRBase21_mature_hsa) | -5.608 | 0.230 | 1.3664656 | -1.1210594 |
| hsa-miR-15b-5p (miRBase21_mature_hsa) | -3.830 | 0.233 | 0.57206756 | -1.365094 |
| hsa-miR-200c-3p (miRBase21_mature_hsa) | -2.748 | 0.235 | 0.96761465 | -0.49069133 |
| hsa-miR-382-3p (miRBase21_mature_hsa) | -3.814 | 0.237 | 1.7358121 | -0.19548924 |
| hsa-miR-4750-5p (miRBase21_mature_hsa) | -3.411 | 0.240 | 0.5194276 | -1.2507883 |
| hsa-miR-502-3p (miRBase21_mature_hsa) | -3.104 | 0.241 | 0.79018897 | -0.8437282 |
| hsa-miR-6847-5p (miRBase21_mature_hsa) | -3.104 | 0.241 | 0.9082627 | -0.7256545 |
| hsa-miR-7854-3p (miRBase21_mature_hsa) | -3.104 | 0.241 | 0.94300866 | -0.6909085 |
| hsa-miR-1538 (miRBase21_mature_hsa) | -3.543 | 0.241 | 1.7350965 | -0.0897308 |
| hsa-miR-556-5p (miRBase21_mature_hsa) | -3.565 | 0.242 | 1.3008455 | -0.5330717 |
| hsa-miR-410-3p (miRBase21_mature_hsa) | 2.250 | 0.244 | -0.2874992 | 0.8826056 |
| hsa-miR-4646-3p (miRBase21_mature_hsa) | 2.250 | 0.244 | -0.32427537 | 0.8458295 |
| hsa-miR-145-5p (miRBase21_mature_hsa) | -3.339 | 0.248 | 0.88247913 | -0.857059 |
| hsa-miR-7706 (miRBase21_mature_hsa) | -2.403 | 0.251 | 0.21336587 | -1.0513393 |
| hsa-miR-185-3p (miRBase21_mature_hsa) | -3.945 | 0.252 | 1.7081714 | -0.271984 |
| hsa-miR-4433a-5p (miRBase21_mature_hsa) | 2.331 | 0.253 | -0.21221197 | 1.0087508 |
| hsa-miR-30d-3p (miRBase21_mature_hsa) | 2.533 | 0.257 | -0.46917003 | 0.87194854 |
| hsa-miR-1255b-5p (miRBase21_mature_hsa) | -3.012 | 0.258 | 2.0466306 | 0.4558159 |
| hsa-miR-6741-3p (miRBase21_mature_hsa) | -5.955 | 0.262 | 0.67848 | -1.8956512 |
| hsa-miR-363-3p (miRBase21_mature_hsa) | -1.759 | 0.264 | 1.0909773 | 0.27656576 |
| hsa-miR-142-3p (miRBase21_mature_hsa) | -2.274 | 0.265 | 0.7389388 | -0.44627306 |
| hsa-miR-323a-3p (miRBase21_mature_hsa) | -2.696 | 0.266 | 0.7811337 | -0.64968765 |
| hsa-miR-877-3p (miRBase21_mature_hsa) | 3.391 | 0.270 | -1.0925705 | 0.66917616 |
| hsa-miR-1273h-3p (miRBase21_mature_hsa) | -2.785 | 0.275 | 0.58117586 | -0.89669806 |
| hsa-miR-3909 (miRBase21_mature_hsa) | -3.369 | 0.277 | 1.3777112 | -0.3746932 |
| hsa-miR-5187-5p (miRBase21_mature_hsa) | -3.517 | 0.278 | 0.7829689 | -1.0311856 |
| hsa-let-7f-1-3p (miRBase21_mature_hsa) | -2.952 | 0.279 | 0.29473883 | -1.2667556 |
| hsa-miR-487a-5p (miRBase21_mature_hsa) | -2.952 | 0.279 | 1.0676862 | -0.49380824 |
| hsa-miR-877-5p (miRBase21_mature_hsa) | -2.952 | 0.279 | 0.113226414 | -1.448268 |
| hsa-miR-6734-5p (miRBase21_mature_hsa) | -2.707 | 0.280 | 1.5777378 | 0.14128096 |
| hsa-miR-6868-3p (miRBase21_mature_hsa) | 2.512 | 0.287 | -0.36971393 | 0.9591036 |
| hsa-miR-450a-5p (miRBase21_mature_hsa) | -2.272 | 0.288 | 0.535475 | -0.64832187 |
| hsa-miR-425-3p (miRBase21_mature_hsa) | 2.993 | 0.288 | -0.31166315 | 1.2701399 |
| hsa-miR-874-3p (miRBase21_mature_hsa) | -2.867 | 0.289 | 1.3194995 | -0.20023902 |
| hsa-miR-7848-3p (miRBase21_mature_hsa) | -3.212 | 0.289 | 0.4777274 | -1.2057548 |
| hsa-miR-493-3p (miRBase21_mature_hsa) | -2.173 | 0.291 | 0.7755985 | -0.3439338 |
| hsa-miR-450b-5p (miRBase21_mature_hsa) | 2.070 | 0.292 | -0.031622957 | 1.0178037 |
| hsa-miR-548ah-3p (miRBase21_mature_hsa) | -3.581 | 0.299 | 0.9454882 | -0.8949595 |
| hsa-miR-576-3p (miRBase21_mature_hsa) | 2.888 | 0.299 | -0.42361563 | 1.1065313 |
| hsa-miR-152-3p (miRBase21_mature_hsa) | -3.772 | 0.303 | 0.6513996 | -1.2637694 |
| hsa-miR-204-3p (miRBase21_mature_hsa) | -3.588 | 0.303 | 1.1716357 | -0.6717251 |
| hsa-miR-4786-5p (miRBase21_mature_hsa) | -2.497 | 0.304 | 0.88842094 | -0.43181106 |
| hsa-miR-3130-3p (miRBase21_mature_hsa) | -2.303 | 0.308 | 0.9541772 | -0.24906227 |
| hsa-miR-4449 (miRBase21_mature_hsa) | -2.303 | 0.308 | 0.7345202 | -0.4687193 |
| hsa-miR-541-3p (miRBase21_mature_hsa) | -2.303 | 0.308 | 0.81896764 | -0.38427177 |
| hsa-miR-4433a-3p (miRBase21_mature_hsa) | -2.952 | 0.311 | 1.0691236 | -0.49237093 |
| hsa-miR-125b-5p (miRBase21_mature_hsa) | -2.183 | 0.315 | 0.94334817 | -0.18270577 |
| hsa-miR-125b-2-3p (miRBase21_mature_hsa) | -2.625 | 0.316 | 1.4899572 | 0.097759195 |
| hsa-miR-505-5p (miRBase21_mature_hsa) | 2.891 | 0.318 | -0.5500962 | 0.98168796 |
| hsa-miR-199b-5p (miRBase21_mature_hsa) | -2.933 | 0.323 | 0.44097188 | -1.1114326 |
| hsa-miR-1238-3p (miRBase21_mature_hsa) | -2.004 | 0.325 | 0.87520313 | -0.1280363 |
| hsa-miR-1976 (miRBase21_mature_hsa) | -2.004 | 0.325 | 0.87520313 | -0.1280363 |
| hsa-miR-3164 (miRBase21_mature_hsa) | -2.004 | 0.325 | 0.5635513 | -0.43968818 |
| hsa-miR-34a-5p (miRBase21_mature_hsa) | -2.004 | 0.325 | 0.87520313 | -0.1280363 |

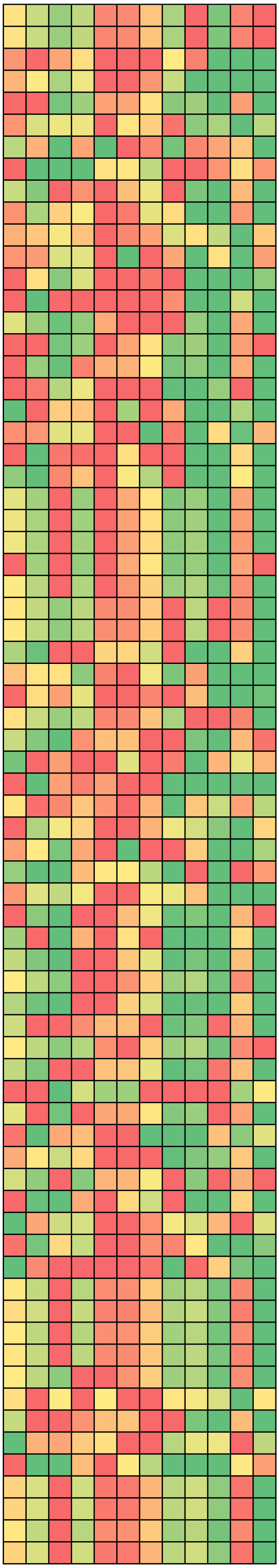

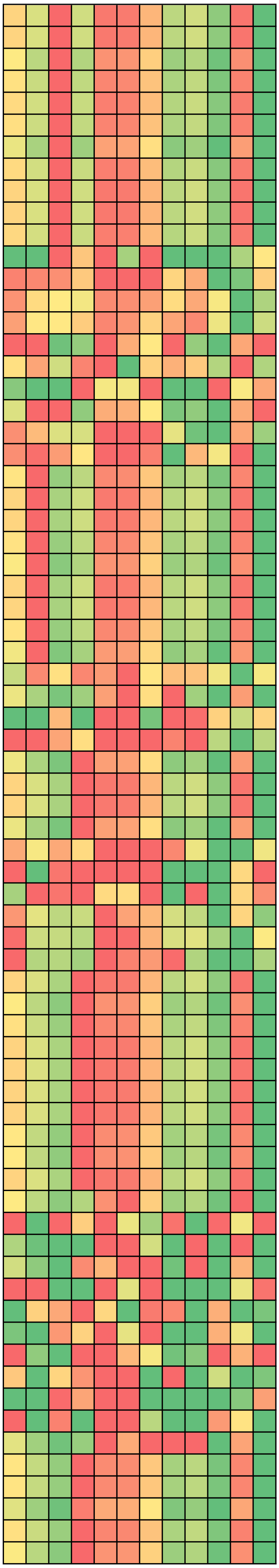

| | | | | |
|---|---|---|---|---|
| hsa-miR-3911 (miRBase21_mature_hsa) | -2.004 | 0.325 | 0.87520313 | -0.1280363 |
| hsa-miR-4497 (miRBase21_mature_hsa) | -2.004 | 0.325 | 0.87520313 | -0.1280363 |
| hsa-miR-4508 (miRBase21_mature_hsa) | -2.004 | 0.325 | 0.48748446 | -0.515755 |
| hsa-miR-4510 (miRBase21_mature_hsa) | -2.004 | 0.325 | 0.671544 | -0.33169547 |
| hsa-miR-598-3p (miRBase21_mature_hsa) | -2.004 | 0.325 | 0.7761593 | -0.2270802 |
| hsa-miR-6514-3p (miRBase21_mature_hsa) | -2.004 | 0.325 | 0.6979606 | -0.30527884 |
| hsa-miR-6515-3p (miRBase21_mature_hsa) | -2.004 | 0.325 | 0.24952802 | -0.75371146 |
| hsa-miR-6736-3p (miRBase21_mature_hsa) | -2.004 | 0.325 | 0.77591634 | -0.22732313 |
| hsa-miR-6750-3p (miRBase21_mature_hsa) | -2.004 | 0.325 | 0.87520313 | -0.1280363 |
| hsa-miR-6821-3p (miRBase21_mature_hsa) | -2.004 | 0.325 | 0.87520313 | -0.1280363 |
| hsa-miR-933 (miRBase21_mature_hsa) | -2.004 | 0.325 | 0.8010113 | -0.20222811 |
| hsa-miR-345-5p (miRBase21_mature_hsa) | -3.186 | 0.331 | 0.0907042 | -1.5808158 |
| hsa-miR-374b-5p (miRBase21_mature_hsa) | -2.302 | 0.334 | 1.5092485 | 0.30657208 |
| hsa-miR-30a-5p (miRBase21_mature_hsa) | -1.878 | 0.344 | 0.59346706 | -0.31588802 |
| hsa-miR-142-5p (miRBase21_mature_hsa) | -1.936 | 0.353 | 0.6654526 | -0.28735974 |
| hsa-miR-6837-3p (miRBase21_mature_hsa) | -3.420 | 0.354 | 2.3150687 | 0.5409195 |
| hsa-miR-22-5p (miRBase21_mature_hsa) | -2.069 | 0.356 | 1.1412964 | 0.09267887 |
| hsa-miR-125b-1-3p (miRBase21_mature_hsa) | 2.656 | 0.358 | -0.8792659 | 0.52973145 |
| hsa-miR-660-5p (miRBase21_mature_hsa) | -2.297 | 0.360 | 1.1693331 | -0.030676553 |
| hsa-miR-671-3p (miRBase21_mature_hsa) | -1.860 | 0.362 | 0.79252845 | -0.102629445 |
| hsa-miR-6747-3p (miRBase21_mature_hsa) | -3.216 | 0.363 | 1.3825935 | -0.30270943 |
| hsa-miR-1270 (miRBase21_mature_hsa) | -2.333 | 0.364 | 0.8427956 | -0.37939295 |
| hsa-miR-3148 (miRBase21_mature_hsa) | -2.333 | 0.364 | 1.0703534 | -0.15183511 |
| hsa-miR-3613-3p (miRBase21_mature_hsa) | -2.333 | 0.364 | 1.0703534 | -0.15183511 |
| hsa-miR-4683 (miRBase21_mature_hsa) | -2.333 | 0.364 | 0.87247527 | -0.3497134 |
| hsa-miR-4723-5p (miRBase21_mature_hsa) | -2.333 | 0.364 | 0.67924374 | -0.5429449 |
| hsa-miR-487b-5p (miRBase21_mature_hsa) | -2.333 | 0.364 | 1.0703534 | -0.15183511 |
| hsa-miR-548aq-3p (miRBase21_mature_hsa) | -2.333 | 0.364 | 1.0703534 | -0.15183511 |
| hsa-miR-6850-5p (miRBase21_mature_hsa) | -2.333 | 0.364 | 0.8572644 | -0.36492416 |
| hsa-miR-708-3p (miRBase21_mature_hsa) | -2.333 | 0.364 | 0.5704115 | -0.6517771 |
| hsa-miR-192-5p (miRBase21_mature_hsa) | -1.924 | 0.364 | 0.74868405 | -0.19528665 |
| hsa-miR-6780a-5p (miRBase21_mature_hsa) | 2.363 | 0.365 | -0.6122091 | 0.62856525 |
| hsa-miR-369-5p (miRBase21_mature_hsa) | 2.986 | 0.366 | -0.63361573 | 0.9445133 |
| hsa-miR-181d-5p (miRBase21_mature_hsa) | -1.908 | 0.367 | 1.8455696 | 0.9138654 |
| hsa-miR-136-3p (miRBase21_mature_hsa) | -2.067 | 0.369 | 0.33825865 | -0.7095506 |
| hsa-miR-194-3p (miRBase21_mature_hsa) | -2.067 | 0.369 | 0.9149284 | -0.13288084 |
| hsa-miR-5582-3p (miRBase21_mature_hsa) | -2.067 | 0.369 | 0.9149284 | -0.13288084 |
| hsa-miR-6513-3p (miRBase21_mature_hsa) | -2.067 | 0.369 | 0.30539137 | -0.7424179 |
| hsa-miR-140-3p (miRBase21_mature_hsa) | -2.128 | 0.371 | 1.0044626 | -0.084852025 |
| hsa-miR-409-5p (miRBase21_mature_hsa) | -2.992 | 0.373 | 2.185526 | 0.6043209 |
| hsa-miR-455-5p (miRBase21_mature_hsa) | -2.463 | 0.378 | 1.8071878 | 0.5065255 |
| hsa-miR-24-3p (miRBase21_mature_hsa) | -1.614 | 0.380 | 0.30790246 | -0.38248947 |
| hsa-miR-629-5p (miRBase21_mature_hsa) | -2.078 | 0.380 | 0.33770183 | -0.7177757 |
| hsa-miR-30e-3p (miRBase21_mature_hsa) | -2.556 | 0.381 | 0.44118875 | -0.9127299 |
| hsa-miR-34c-5p (miRBase21_mature_hsa) | -1.906 | 0.386 | 0.81065243 | -0.12016422 |
| hsa-miR-452-5p (miRBase21_mature_hsa) | -1.906 | 0.386 | 0.42097017 | -0.5098465 |
| hsa-miR-4669 (miRBase21_mature_hsa) | -1.906 | 0.386 | 0.60603905 | -0.32477757 |
| hsa-miR-4784 (miRBase21_mature_hsa) | -1.906 | 0.386 | 0.81065243 | -0.12016422 |
| hsa-miR-488-3p (miRBase21_mature_hsa) | -1.906 | 0.386 | 0.81065243 | -0.12016422 |
| hsa-miR-5192 (miRBase21_mature_hsa) | -1.906 | 0.386 | 0.81065243 | -0.12016422 |
| hsa-miR-5196-3p (miRBase21_mature_hsa) | -1.906 | 0.386 | 0.81065243 | -0.12016422 |
| hsa-miR-6505-3p (miRBase21_mature_hsa) | -1.906 | 0.386 | 0.5063839 | -0.4244328 |
| hsa-miR-668-3p (miRBase21_mature_hsa) | -1.906 | 0.386 | 0.47814268 | -0.45267397 |
| hsa-miR-6827-3p (miRBase21_mature_hsa) | -1.906 | 0.386 | 0.81065243 | -0.12016422 |
| hsa-miR-128-1-5p (miRBase21_mature_hsa) | 3.001 | 0.389 | -0.37142244 | 1.2141325 |
| hsa-miR-107 (miRBase21_mature_hsa) | -2.434 | 0.391 | 1.6015306 | 0.318061 |
| hsa-miR-548am-3p (miRBase21_mature_hsa) | 3.526 | 0.395 | -0.7534103 | 1.0648092 |
| hsa-miR-642a-3p (miRBase21_mature_hsa) | 2.515 | 0.403 | -0.34564316 | 0.98470104 |
| hsa-miR-374a-3p (miRBase21_mature_hsa) | -3.296 | 0.404 | 1.0550418 | -0.66562545 |
| hsa-miR-145-3p (miRBase21_mature_hsa) | -2.630 | 0.408 | 0.20312998 | -1.1917365 |
| hsa-miR-4473 (miRBase21_mature_hsa) | -2.803 | 0.411 | 0.5945725 | -0.8922569 |
| hsa-miR-143-5p (miRBase21_mature_hsa) | -2.509 | 0.413 | 1.6556214 | 0.32858548 |
| hsa-miR-151a-5p (miRBase21_mature_hsa) | -2.763 | 0.419 | 0.0741321 | -1.3920293 |
| hsa-miR-641 (miRBase21_mature_hsa) | -3.325 | 0.419 | 0.16630788 | -1.5669804 |
| hsa-miR-3191-3p (miRBase21_mature_hsa) | -2.753 | 0.432 | 0.9616537 | -0.4994375 |
| hsa-miR-605-3p (miRBase21_mature_hsa) | 2.427 | 0.432 | -0.093015276 | 1.1861663 |
| hsa-let-7e-3p (miRBase21_mature_hsa) | -1.660 | 0.437 | 0.32877785 | -0.40203887 |
| hsa-miR-1277-5p (miRBase21_mature_hsa) | -1.660 | 0.437 | 0.34463194 | -0.38618478 |
| hsa-miR-1278 (miRBase21_mature_hsa) | -1.660 | 0.437 | -0.15331312 | -0.8841298 |
| hsa-miR-200b-5p (miRBase21_mature_hsa) | -1.660 | 0.437 | 0.40564638 | -0.32517034 |
| hsa-miR-205-5p (miRBase21_mature_hsa) | -1.660 | 0.437 | 0.1916025 | -0.5392142 |

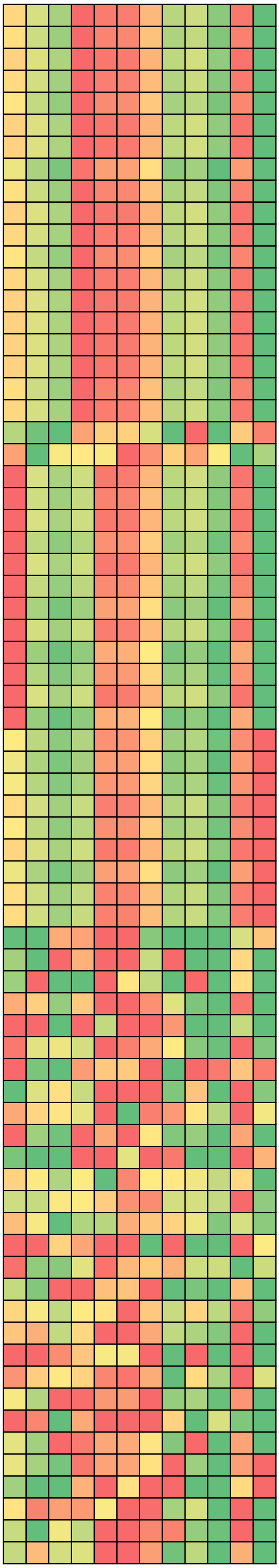

| | | | | |
|---|---|---|---|---|
| hsa-miR-3150b-3p (miRBase21_mature_hsa) | -1.660 | 0.437 | 0.55819976 | -0.17261693 |
| hsa-miR-365b-3p (miRBase21_mature_hsa) | -1.660 | 0.437 | 0.42738554 | -0.3034312 |
| hsa-miR-4686 (miRBase21_mature_hsa) | -1.660 | 0.437 | 0.6323916 | -0.09842511 |
| hsa-miR-4700-5p (miRBase21_mature_hsa) | -1.660 | 0.437 | 0.5101289 | -0.22068779 |
| hsa-miR-4726-5p (miRBase21_mature_hsa) | -1.660 | 0.437 | 0.3247235 | -0.4060932 |
| hsa-miR-4772-5p (miRBase21_mature_hsa) | -1.660 | 0.437 | 0.6323916 | -0.09842511 |
| hsa-miR-4787-3p (miRBase21_mature_hsa) | -1.660 | 0.437 | 0.6323916 | -0.09842511 |
| hsa-miR-5004-3p (miRBase21_mature_hsa) | -1.660 | 0.437 | 0.011562881 | -0.71925384 |
| hsa-miR-516b-5p (miRBase21_mature_hsa) | -1.660 | 0.437 | 0.40954587 | -0.32127085 |
| hsa-miR-550a-3-5p (miRBase21_mature_hsa) | -1.660 | 0.437 | 0.6323916 | -0.09842511 |
| hsa-miR-627-3p (miRBase21_mature_hsa) | -1.660 | 0.437 | 0.5449822 | -0.1858345 |
| hsa-miR-6505-5p (miRBase21_mature_hsa) | -1.660 | 0.437 | 0.34409568 | -0.38672107 |
| hsa-miR-6765-5p (miRBase21_mature_hsa) | -1.660 | 0.437 | 0.55819976 | -0.17261693 |
| hsa-miR-6812-5p (miRBase21_mature_hsa) | -1.660 | 0.437 | 0.6323916 | -0.09842511 |
| hsa-miR-6837-5p (miRBase21_mature_hsa) | -1.660 | 0.437 | 0.6323916 | -0.09842511 |
| hsa-miR-6849-3p (miRBase21_mature_hsa) | -1.660 | 0.437 | 0.6323916 | -0.09842511 |
| hsa-miR-935 (miRBase21_mature_hsa) | -1.660 | 0.437 | 0.6323916 | -0.09842511 |
| hsa-miR-9-3p (miRBase21_mature_hsa) | -1.660 | 0.437 | 0.43951693 | -0.2912998 |
| hsa-miR-942-3p (miRBase21_mature_hsa) | -1.660 | 0.437 | 0.54401433 | -0.18680236 |
| hsa-miR-1229-3p (miRBase21_mature_hsa) | 1.853 | 0.437 | -0.71060157 | 0.17959443 |
| hsa-miR-320c (miRBase21_mature_hsa) | 2.207 | 0.437 | -0.8837892 | 0.25832766 |
| hsa-miR-103a-2-5p (miRBase21_mature_hsa) | -2.104 | 0.440 | 0.9378011 | -0.13567017 |
| hsa-miR-1250-5p (miRBase21_mature_hsa) | -2.104 | 0.440 | 0.80624866 | -0.2672226 |
| hsa-miR-299-5p (miRBase21_mature_hsa) | -2.104 | 0.440 | 0.9378011 | -0.13567017 |
| hsa-miR-30c-2-3p (miRBase21_mature_hsa) | -2.104 | 0.440 | 0.594181 | -0.4792903 |
| hsa-miR-3190-3p (miRBase21_mature_hsa) | -2.104 | 0.440 | 0.9378011 | -0.13567017 |
| hsa-miR-3675-5p (miRBase21_mature_hsa) | -2.104 | 0.440 | 0.6878227 | -0.38564858 |
| hsa-miR-4654 (miRBase21_mature_hsa) | -2.104 | 0.440 | 0.32959804 | -0.7438732 |
| hsa-miR-4659b-3p (miRBase21_mature_hsa) | -2.104 | 0.440 | 0.8636093 | -0.20986198 |
| hsa-miR-548d-5p (miRBase21_mature_hsa) | -2.104 | 0.440 | 0.15573741 | -0.9177338 |
| hsa-miR-6767-5p (miRBase21_mature_hsa) | -2.104 | 0.440 | 0.46332476 | -0.6101466 |
| hsa-miR-7702 (miRBase21_mature_hsa) | -2.104 | 0.440 | 0.9378011 | -0.13567017 |
| hsa-miR-9-5p (miRBase21_mature_hsa) | -2.104 | 0.440 | 0.12319841 | -0.95027286 |
| hsa-miR-130b-3p (miRBase21_mature_hsa) | 1.551 | 0.442 | -0.3929906 | 0.23978893 |
| hsa-miR-27a-5p (miRBase21_mature_hsa) | 1.551 | 0.442 | -0.57057536 | 0.062204152 |
| hsa-miR-301a-5p (miRBase21_mature_hsa) | 1.551 | 0.442 | -0.47428334 | 0.15849622 |
| hsa-miR-4429 (miRBase21_mature_hsa) | 1.551 | 0.442 | -0.09716224 | 0.5356173 |
| hsa-miR-4742-5p (miRBase21_mature_hsa) | 1.551 | 0.442 | -0.3365209 | 0.29625863 |
| hsa-miR-4758-3p (miRBase21_mature_hsa) | 1.551 | 0.442 | -0.022970427 | 0.6098091 |
| hsa-miR-500a-3p (miRBase21_mature_hsa) | 1.551 | 0.442 | -0.61056864 | 0.022210922 |
| hsa-miR-615-5p (miRBase21_mature_hsa) | 1.551 | 0.442 | -0.14380738 | 0.48897216 |
| hsa-miR-6733-5p (miRBase21_mature_hsa) | 1.551 | 0.442 | -0.118901365 | 0.51387817 |
| hsa-miR-200b-3p (miRBase21_mature_hsa) | -2.468 | 0.445 | 0.29476237 | -1.0083647 |
| hsa-miR-132-3p (miRBase21_mature_hsa) | -2.623 | 0.445 | 1.1003382 | -0.2908321 |
| hsa-miR-504-5p (miRBase21_mature_hsa) | -2.731 | 0.446 | 0.4416517 | -1.007722 |
| hsa-miR-127-3p (miRBase21_mature_hsa) | -1.680 | 0.446 | 0.5745017 | -0.17363414 |
| hsa-miR-4746-5p (miRBase21_mature_hsa) | -2.796 | 0.447 | 0.8242806 | -0.65910375 |
| hsa-miR-26b-5p (miRBase21_mature_hsa) | -1.629 | 0.449 | 0.6470829 | -0.056787625 |
| hsa-miR-550a-5p (miRBase21_mature_hsa) | 2.098 | 0.451 | 0.33422297 | 1.4029876 |
| hsa-miR-574-3p (miRBase21_mature_hsa) | 2.269 | 0.452 | -0.74251986 | 0.43974963 |
| hsa-miR-16-5p (miRBase21_mature_hsa) | -1.746 | 0.452 | 0.7033551 | -0.10053187 |
| hsa-miR-365a-5p (miRBase21_mature_hsa) | -2.463 | 0.453 | 1.1336981 | -0.1666535 |
| hsa-miR-582-3p (miRBase21_mature_hsa) | 2.766 | 0.453 | -0.43558365 | 1.0321223 |
| hsa-miR-375 (miRBase21_mature_hsa) | 1.448 | 0.461 | -0.68507975 | -0.15080652 |
| hsa-miR-320b (miRBase21_mature_hsa) | 1.342 | 0.466 | -0.18639788 | 0.23818953 |
| hsa-miR-10b-5p (miRBase21_mature_hsa) | 1.414 | 0.467 | -0.7688207 | -0.26855722 |
| hsa-miR-374a-5p (miRBase21_mature_hsa) | -2.356 | 0.467 | 1.7280232 | 0.49162424 |
| hsa-miR-126-5p (miRBase21_mature_hsa) | -1.805 | 0.468 | 0.09576525 | -0.75619966 |
| hsa-miR-95-3p (miRBase21_mature_hsa) | -2.030 | 0.470 | 0.5635049 | -0.4581655 |
| hsa-miR-92b-3p (miRBase21_mature_hsa) | 1.359 | 0.474 | -0.08873621 | 0.35424915 |
| hsa-miR-4433b-3p (miRBase21_mature_hsa) | -1.627 | 0.476 | 0.7255758 | 0.023095813 |
| hsa-miR-4647 (miRBase21_mature_hsa) | -2.421 | 0.477 | 1.6808288 | 0.40531608 |
| hsa-miR-222-3p (miRBase21_mature_hsa) | -1.449 | 0.479 | 0.76261127 | 0.22718488 |
| hsa-miR-3690 (miRBase21_mature_hsa) | -2.030 | 0.480 | 1.2004848 | 0.17881428 |
| hsa-miR-3120-5p (miRBase21_mature_hsa) | -2.644 | 0.482 | 0.76409984 | -0.6388796 |
| hsa-miR-96-5p (miRBase21_mature_hsa) | -1.926 | 0.482 | 0.97261167 | 0.026788639 |
| hsa-miR-29b-3p (miRBase21_mature_hsa) | 1.621 | 0.487 | -0.055525016 | 0.64095587 |
| hsa-miR-326 (miRBase21_mature_hsa) | 2.114 | 0.487 | -0.31484357 | 0.7648776 |
| hsa-miR-6741-5p (miRBase21_mature_hsa) | -2.607 | 0.489 | 0.83264387 | -0.549964 |
| hsa-miR-6852-5p (miRBase21_mature_hsa) | 2.530 | 0.489 | -0.8728129 | 0.46614677 |
| hsa-miR-543 (miRBase21_mature_hsa) | -1.599 | 0.493 | 0.3096542 | -0.36754897 |

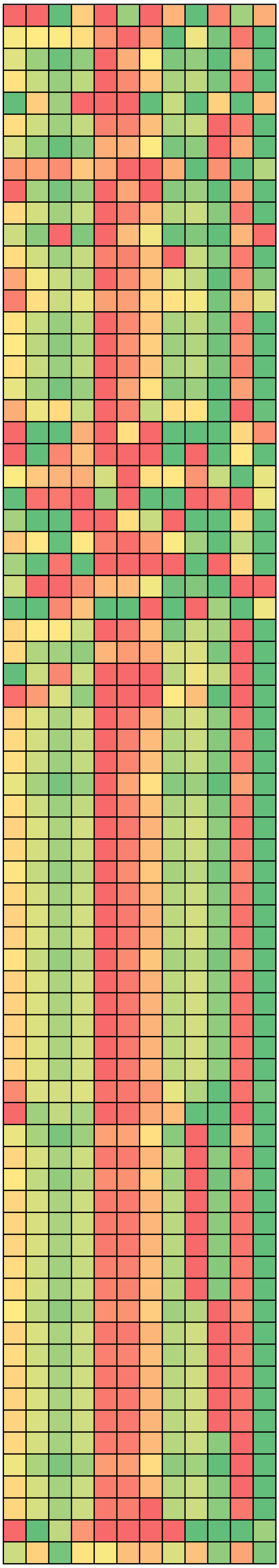

| | | | | |
|---|---|---|---|---|
| hsa-miR-338-5p (miRBase21_mature_hsa) | -2.205 | 0.497 | 1.0953516 | -0.04543684 |
| hsa-miR-328-3p (miRBase21_mature_hsa) | -1.467 | 0.499 | 0.28789195 | -0.2646095 |
| hsa-miR-369-3p (miRBase21_mature_hsa) | -2.228 | 0.503 | 0.25186998 | -0.90369874 |
| hsa-miR-548ar-3p (miRBase21_mature_hsa) | -2.228 | 0.503 | 0.7979016 | -0.35766712 |
| hsa-miR-485-3p (miRBase21_mature_hsa) | -2.193 | 0.507 | -0.025390238 | -1.158264 |
| hsa-miR-3936 (miRBase21_mature_hsa) | 1.562 | 0.509 | -0.14649701 | 0.49683768 |
| hsa-miR-4685-3p (miRBase21_mature_hsa) | 1.562 | 0.509 | -0.83397484 | -0.19064017 |
| hsa-miR-320d (miRBase21_mature_hsa) | -1.728 | 0.512 | 1.0788594 | 0.28961262 |
| hsa-miR-4661-5p (miRBase21_mature_hsa) | -2.394 | 0.513 | 1.122134 | -0.13737747 |
| hsa-miR-544b (miRBase21_mature_hsa) | -2.103 | 0.513 | 0.85879123 | -0.2137699 |
| hsa-miR-146a-3p (miRBase21_mature_hsa) | -1.836 | 0.513 | 0.5080274 | -0.36821944 |
| hsa-miR-490-3p (miRBase21_mature_hsa) | 1.611 | 0.526 | -0.12314523 | 0.56444824 |
| hsa-miR-126-3p (miRBase21_mature_hsa) | -1.425 | 0.527 | 0.28951997 | -0.22168629 |
| hsa-miR-423-3p (miRBase21_mature_hsa) | -1.292 | 0.529 | 0.4056004 | 0.036314897 |
| hsa-miR-374b-3p (miRBase21_mature_hsa) | -1.939 | 0.530 | 0.59652853 | -0.35904023 |
| hsa-miR-4498 (miRBase21_mature_hsa) | -1.939 | 0.530 | 0.44844744 | -0.50712126 |
| hsa-miR-4688 (miRBase21_mature_hsa) | -1.939 | 0.530 | 0.6198945 | -0.33567423 |
| hsa-miR-6766-5p (miRBase21_mature_hsa) | -1.939 | 0.530 | 0.17324105 | -0.7823277 |
| hsa-miR-182-5p (miRBase21_mature_hsa) | -1.451 | 0.531 | 0.45160767 | -0.085895784 |
| hsa-miR-454-5p (miRBase21_mature_hsa) | -2.155 | 0.533 | 0.8696894 | -0.2380774 |
| hsa-miR-664b-5p (miRBase21_mature_hsa) | -2.420 | 0.534 | 1.8107781 | 0.53580344 |
| hsa-miR-183-5p (miRBase21_mature_hsa) | -1.591 | 0.538 | 0.34889814 | -0.32107592 |
| hsa-miR-939-5p (miRBase21_mature_hsa) | 2.250 | 0.538 | 0.3099666 | 1.4797189 |
| hsa-miR-1285-3p (miRBase21_mature_hsa) | -1.856 | 0.549 | -0.07522142 | -0.96708864 |
| hsa-miR-409-3p (miRBase21_mature_hsa) | -1.420 | 0.556 | 0.07583273 | -0.42973453 |
| hsa-miR-146b-3p (miRBase21_mature_hsa) | 2.125 | 0.560 | 0.20975754 | 1.2972258 |
| hsa-miR-5010-5p (miRBase21_mature_hsa) | -1.911 | 0.562 | 1.5909321 | 0.6565474 |
| hsa-miR-511-5p (miRBase21_mature_hsa) | 1.960 | 0.566 | -1.7172985 | -0.7467981 |
| hsa-miR-340-3p (miRBase21_mature_hsa) | -1.488 | 0.566 | 0.4760461 | -0.09713312 |
| hsa-miR-139-3p (miRBase21_mature_hsa) | 1.445 | 0.567 | -0.49133366 | 0.03977237 |
| hsa-miR-6843-3p (miRBase21_mature_hsa) | 2.080 | 0.567 | -0.3115119 | 0.7453972 |
| hsa-miR-548o-3p (miRBase21_mature_hsa) | -2.344 | 0.568 | 1.0997833 | -0.12923732 |
| hsa-miR-1185-2-3p (miRBase21_mature_hsa) | -1.688 | 0.568 | 0.6544531 | -0.10111553 |
| hsa-miR-3157-3p (miRBase21_mature_hsa) | -1.688 | 0.568 | 0.53420186 | -0.2213668 |
| hsa-miR-377-3p (miRBase21_mature_hsa) | -1.688 | 0.568 | 0.47169122 | -0.28387743 |
| hsa-miR-379-3p (miRBase21_mature_hsa) | -1.688 | 0.568 | -0.020137608 | -0.7757063 |
| hsa-miR-4504 (miRBase21_mature_hsa) | -1.688 | 0.568 | 0.4597365 | -0.29583213 |
| hsa-miR-4521 (miRBase21_mature_hsa) | -1.688 | 0.568 | 0.6544531 | -0.10111553 |
| hsa-miR-4676-3p (miRBase21_mature_hsa) | -1.688 | 0.568 | 0.5542719 | -0.20129679 |
| hsa-miR-4762-3p (miRBase21_mature_hsa) | -1.688 | 0.568 | 0.371829 | -0.38373956 |
| hsa-miR-487a-3p (miRBase21_mature_hsa) | -1.688 | 0.568 | 0.55417603 | -0.20139258 |
| hsa-miR-509-3p (miRBase21_mature_hsa) | -1.688 | 0.568 | 0.6544531 | -0.10111553 |
| hsa-miR-556-3p (miRBase21_mature_hsa) | -1.688 | 0.568 | 0.6544531 | -0.10111553 |
| hsa-miR-628-5p (miRBase21_mature_hsa) | -1.688 | 0.568 | 0.38959336 | -0.3659753 |
| hsa-miR-6503-5p (miRBase21_mature_hsa) | -1.688 | 0.568 | 0.6544531 | -0.10111553 |
| hsa-miR-6736-5p (miRBase21_mature_hsa) | -1.688 | 0.568 | 0.6544531 | -0.10111553 |
| hsa-miR-6764-5p (miRBase21_mature_hsa) | -1.688 | 0.568 | 0.6544531 | -0.10111553 |
| hsa-miR-6787-5p (miRBase21_mature_hsa) | -1.688 | 0.568 | 0.6544531 | -0.10111553 |
| hsa-miR-892a (miRBase21_mature_hsa) | -1.688 | 0.568 | 0.6544531 | -0.10111553 |
| hsa-miR-744-5p (miRBase21_mature_hsa) | -1.428 | 0.569 | 0.37614852 | -0.13756731 |
| hsa-let-7g-5p (miRBase21_mature_hsa) | -1.755 | 0.571 | 0.65881115 | -0.15298073 |
| hsa-miR-1914-5p (miRBase21_mature_hsa) | 1.511 | 0.572 | -0.62913203 | -0.033537727 |
| hsa-miR-378f (miRBase21_mature_hsa) | 1.511 | 0.572 | -0.0173118 | 0.5782825 |
| hsa-miR-4306 (miRBase21_mature_hsa) | 1.511 | 0.572 | -0.31315887 | 0.28243545 |
| hsa-miR-4326 (miRBase21_mature_hsa) | 1.511 | 0.572 | -0.0173118 | 0.5782825 |
| hsa-miR-4755-3p (miRBase21_mature_hsa) | 1.511 | 0.572 | -0.0173118 | 0.5782825 |
| hsa-miR-550b-3p (miRBase21_mature_hsa) | 1.511 | 0.572 | -0.0173118 | 0.5782825 |
| hsa-miR-6876-3p (miRBase21_mature_hsa) | 1.511 | 0.572 | -0.10949553 | 0.4860988 |
| hsa-miR-6885-3p (miRBase21_mature_hsa) | 1.511 | 0.572 | -0.116355695 | 0.47923863 |
| hsa-miR-3691-5p (miRBase21_mature_hsa) | 1.415 | 0.574 | -0.35805285 | 0.14242467 |
| hsa-miR-542-5p (miRBase21_mature_hsa) | 1.415 | 0.574 | -0.002837515 | 0.49764007 |
| hsa-miR-6734-3p (miRBase21_mature_hsa) | 1.415 | 0.574 | -0.13384962 | 0.36662793 |
| hsa-miR-675-3p (miRBase21_mature_hsa) | 1.415 | 0.574 | -0.002837515 | 0.49764007 |
| hsa-miR-7113-5p (miRBase21_mature_hsa) | 1.415 | 0.574 | -0.002837515 | 0.49764007 |
| hsa-miR-7703 (miRBase21_mature_hsa) | 1.415 | 0.574 | -0.002837515 | 0.49764007 |
| hsa-miR-2278 (miRBase21_mature_hsa) | 1.853 | 0.576 | -0.062039144 | 0.8274778 |
| hsa-miR-6735-5p (miRBase21_mature_hsa) | 1.853 | 0.576 | -0.5720414 | 0.31747553 |
| hsa-miR-6740-5p (miRBase21_mature_hsa) | 1.853 | 0.576 | -0.062039144 | 0.8274778 |
| hsa-miR-6818-5p (miRBase21_mature_hsa) | 1.677 | 0.578 | -0.040225506 | 0.7059446 |
| hsa-miR-323b-3p (miRBase21_mature_hsa) | -2.356 | 0.579 | 0.65572613 | -0.58030725 |
| hsa-miR-125a-5p (miRBase21_mature_hsa) | 1.250 | 0.579 | -0.38097963 | -0.058761798 |

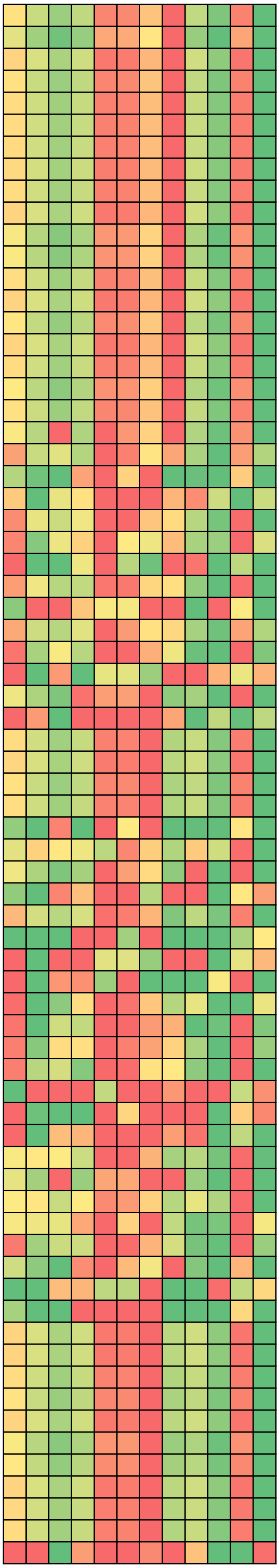

| | | | | |
|---|---|---|---|---|
| hsa-miR-1237-3p (miRBase21_mature_hsa) | 1.459 | 0.581 | -0.20771429 | 0.33702204 |
| hsa-miR-1249-3p (miRBase21_mature_hsa) | 1.459 | 0.581 | -0.73580825 | -0.19107196 |
| hsa-miR-1275 (miRBase21_mature_hsa) | 1.459 | 0.581 | -0.00957253 | 0.5351638 |
| hsa-miR-1307-5p (miRBase21_mature_hsa) | 1.459 | 0.581 | -0.20879817 | 0.3359382 |
| hsa-miR-195-5p (miRBase21_mature_hsa) | 1.459 | 0.581 | -0.12982377 | 0.4149126 |
| hsa-miR-2116-3p (miRBase21_mature_hsa) | 1.459 | 0.581 | -0.11390886 | 0.43082753 |
| hsa-miR-21-3p (miRBase21_mature_hsa) | 1.459 | 0.581 | -0.108616464 | 0.4361199 |
| hsa-miR-3135a (miRBase21_mature_hsa) | 1.459 | 0.581 | -0.083764344 | 0.460972 |
| hsa-miR-3154 (miRBase21_mature_hsa) | 1.459 | 0.581 | -0.11872109 | 0.4260153 |
| hsa-miR-376a-3p (miRBase21_mature_hsa) | 1.459 | 0.581 | -0.00957253 | 0.5351638 |
| hsa-miR-378d (miRBase21_mature_hsa) | 1.459 | 0.581 | -0.46180737 | 0.08292898 |
| hsa-miR-412-5p (miRBase21_mature_hsa) | 1.459 | 0.581 | -0.42605826 | 0.11867807 |
| hsa-miR-424-5p (miRBase21_mature_hsa) | 1.459 | 0.581 | -0.14058468 | 0.40415165 |
| hsa-miR-4638-5p (miRBase21_mature_hsa) | 1.459 | 0.581 | -0.00957253 | 0.5351638 |
| hsa-miR-4644 (miRBase21_mature_hsa) | 1.459 | 0.581 | -0.28214955 | 0.2625868 |
| hsa-miR-4727-3p (miRBase21_mature_hsa) | 1.459 | 0.581 | -0.00957253 | 0.5351638 |
| hsa-miR-627-5p (miRBase21_mature_hsa) | 1.459 | 0.581 | -0.10930792 | 0.4354284 |
| hsa-miR-6514-5p (miRBase21_mature_hsa) | 1.459 | 0.581 | -0.38880765 | 0.15592873 |
| hsa-miR-6819-3p (miRBase21_mature_hsa) | 1.459 | 0.581 | -0.19411302 | 0.35062334 |
| hsa-miR-1288-3p (miRBase21_mature_hsa) | -1.767 | 0.582 | 1.0795286 | 0.25809574 |
| hsa-miR-146a-5p (miRBase21_mature_hsa) | -1.461 | 0.582 | 0.42482004 | -0.122627184 |
| hsa-miR-6516-5p (miRBase21_mature_hsa) | -1.888 | 0.585 | 0.09619112 | -0.8206658 |
| hsa-miR-99b-3p (miRBase21_mature_hsa) | 1.854 | 0.585 | -0.30609918 | 0.584855 |
| hsa-miR-106b-3p (miRBase21_mature_hsa) | -1.394 | 0.587 | 0.52789074 | 0.048434418 |
| hsa-miR-17-5p (miRBase21_mature_hsa) | -1.496 | 0.592 | 0.73446906 | 0.15373993 |
| hsa-miR-1468-5p (miRBase21_mature_hsa) | -1.969 | 0.593 | -0.2638548 | -1.2409601 |
| hsa-miR-134-5p (miRBase21_mature_hsa) | -1.394 | 0.593 | 0.21922204 | -0.25968412 |
| hsa-miR-6515-5p (miRBase21_mature_hsa) | -1.809 | 0.594 | 1.2443702 | 0.38911945 |
| hsa-miR-26a-5p (miRBase21_mature_hsa) | -1.321 | 0.595 | 0.23371024 | -0.16819419 |
| hsa-let-7d-5p (miRBase21_mature_hsa) | -1.530 | 0.595 | 0.522298 | -0.09160066 |
| hsa-miR-106b-5p (miRBase21_mature_hsa) | 1.698 | 0.599 | -0.04304585 | 0.7211426 |
| hsa-miR-24-1-5p (miRBase21_mature_hsa) | 1.903 | 0.600 | 0.040005613 | 0.9682345 |
| hsa-let-7a-3p (miRBase21_mature_hsa) | -1.915 | 0.601 | 0.7282831 | -0.20891237 |
| hsa-miR-147b (miRBase21_mature_hsa) | 1.583 | 0.602 | -0.1276902 | 0.5349138 |
| hsa-miR-6500-3p (miRBase21_mature_hsa) | 1.583 | 0.602 | -0.027508939 | 0.635095 |
| hsa-miR-6511a-5p (miRBase21_mature_hsa) | 1.583 | 0.602 | -0.20799181 | 0.45461214 |
| hsa-miR-6892-5p (miRBase21_mature_hsa) | 1.583 | 0.602 | -0.14149407 | 0.5211099 |
| hsa-miR-411-3p (miRBase21_mature_hsa) | -1.798 | 0.602 | -0.22578092 | -1.0722033 |
| hsa-miR-486-5p (miRBase21_mature_hsa) | 1.241 | 0.605 | -0.1958981 | 0.1153184 |
| hsa-miR-6862-5p (miRBase21_mature_hsa) | 1.972 | 0.609 | 0.072210215 | 1.0515348 |
| hsa-miR-25-5p (miRBase21_mature_hsa) | 1.678 | 0.610 | 0.36352208 | 1.1103569 |
| hsa-miR-128-3p (miRBase21_mature_hsa) | -1.345 | 0.610 | 0.1383216 | -0.2887603 |
| hsa-miR-19a-3p (miRBase21_mature_hsa) | -2.033 | 0.612 | -0.59737724 | -1.6209493 |
| hsa-miR-1294 (miRBase21_mature_hsa) | -1.652 | 0.613 | 0.90135384 | 0.17710526 |
| hsa-miR-7110-3p (miRBase21_mature_hsa) | -2.031 | 0.614 | 0.20866573 | -0.8132656 |
| hsa-miR-181b-5p (miRBase21_mature_hsa) | -1.921 | 0.614 | -0.2358371 | -1.1773491 |
| hsa-miR-20a-5p (miRBase21_mature_hsa) | -1.870 | 0.616 | 0.42829764 | -0.4746698 |
| hsa-miR-199a-3p (miRBase21_mature_hsa) | -1.509 | 0.619 | 0.86604476 | 0.27254966 |
| hsa-miR-103a-3p (miRBase21_mature_hsa) | -1.558 | 0.619 | 0.3971035 | -0.24226798 |
| hsa-miR-18a-3p (miRBase21_mature_hsa) | 1.659 | 0.629 | 1.4671128 | 2.1976743 |
| hsa-miR-210-3p (miRBase21_mature_hsa) | 1.851 | 0.629 | 0.39126176 | 1.2795331 |
| hsa-miR-23a-5p (miRBase21_mature_hsa) | -1.929 | 0.630 | 1.1595345 | 0.2113299 |
| hsa-miR-130b-5p (miRBase21_mature_hsa) | -1.321 | 0.632 | 0.2881979 | -0.11358978 |
| hsa-miR-6877-5p (miRBase21_mature_hsa) | 1.899 | 0.635 | 0.5892931 | 1.5147264 |
| hsa-miR-16-2-3p (miRBase21_mature_hsa) | -1.258 | 0.637 | 0.16552025 | -0.1656983 |
| hsa-miR-29a-3p (miRBase21_mature_hsa) | -1.314 | 0.640 | 0.5186715 | 0.12509981 |
| hsa-miR-98-5p (miRBase21_mature_hsa) | -1.429 | 0.646 | 0.47925016 | -0.035504255 |
| hsa-miR-487b-3p (miRBase21_mature_hsa) | -1.828 | 0.647 | 0.7143244 | -0.15621422 |
| hsa-miR-760 (miRBase21_mature_hsa) | 1.503 | 0.649 | -0.9221103 | -0.33431867 |
| hsa-miR-431-5p (miRBase21_mature_hsa) | -2.029 | 0.650 | 0.7268197 | -0.29399917 |
| hsa-miR-1193 (miRBase21_mature_hsa) | 1.434 | 0.652 | -0.005769766 | 0.51397705 |
| hsa-miR-3614-3p (miRBase21_mature_hsa) | 1.434 | 0.652 | -0.005769766 | 0.51397705 |
| hsa-miR-4710 (miRBase21_mature_hsa) | 1.434 | 0.652 | -0.13732219 | 0.38242462 |
| hsa-miR-552-3p (miRBase21_mature_hsa) | 1.434 | 0.652 | -0.21668753 | 0.3030593 |
| hsa-miR-6509-5p (miRBase21_mature_hsa) | 1.434 | 0.652 | -0.005769766 | 0.51397705 |
| hsa-miR-6803-3p (miRBase21_mature_hsa) | 1.434 | 0.652 | -0.4386015 | 0.08114529 |
| hsa-miR-6806-3p (miRBase21_mature_hsa) | 1.434 | 0.652 | -0.3033874 | 0.2163594 |
| hsa-miR-6827-5p (miRBase21_mature_hsa) | 1.434 | 0.652 | -0.005769766 | 0.51397705 |
| hsa-miR-6894-5p (miRBase21_mature_hsa) | 1.434 | 0.652 | -0.093179196 | 0.42656764 |
| hsa-miR-7107-3p (miRBase21_mature_hsa) | 1.434 | 0.652 | -0.11975495 | 0.39999184 |
| hsa-miR-589-5p (miRBase21_mature_hsa) | -1.904 | 0.654 | 1.3053508 | 0.37612423 |

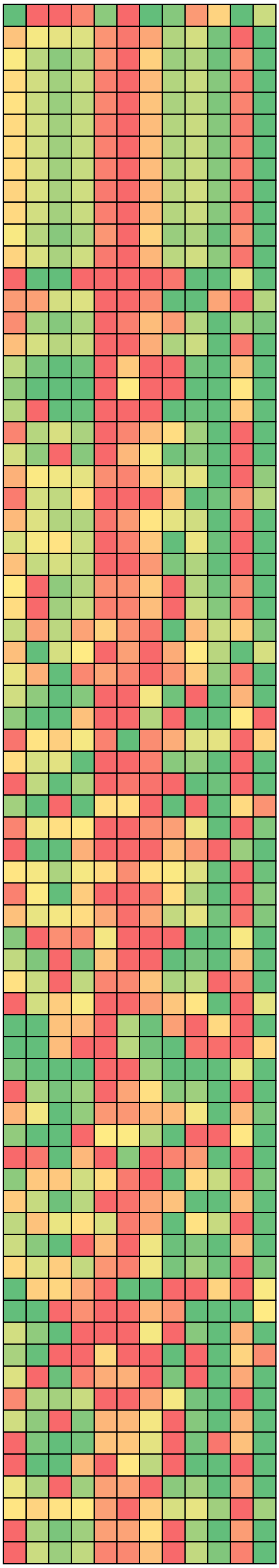

| | | | | |
|---|---|---|---|---|
| hsa-miR-1180-3p (miRBase21_mature_hsa) | -1.861 | 0.654 | -0.31568837 | -1.2119064 |
| hsa-miR-1307-3p (miRBase21_mature_hsa) | -1.266 | 0.656 | 0.21831237 | -0.122413024 |
| hsa-miR-135a-3p (miRBase21_mature_hsa) | 1.486 | 0.657 | -0.4089956 | 0.16215156 |
| hsa-miR-3187-3p (miRBase21_mature_hsa) | 1.486 | 0.657 | -0.1095225 | 0.46162462 |
| hsa-miR-4677-3p (miRBase21_mature_hsa) | 1.486 | 0.657 | -0.21676221 | 0.3543849 |
| hsa-miR-4734 (miRBase21_mature_hsa) | 1.486 | 0.657 | -0.10348333 | 0.4676638 |
| hsa-miR-4753-5p (miRBase21_mature_hsa) | 1.486 | 0.657 | -0.13506612 | 0.43608102 |
| hsa-miR-4804-5p (miRBase21_mature_hsa) | 1.486 | 0.657 | -0.113326944 | 0.4578202 |
| hsa-miR-522-5p (miRBase21_mature_hsa) | 1.486 | 0.657 | -0.013591563 | 0.55755556 |
| hsa-miR-6753-3p (miRBase21_mature_hsa) | 1.486 | 0.657 | -0.09179023 | 0.47935688 |
| hsa-miR-6777-3p (miRBase21_mature_hsa) | 1.486 | 0.657 | -0.4474666 | 0.123680525 |
| hsa-miR-6889-3p (miRBase21_mature_hsa) | 1.486 | 0.657 | -0.013591563 | 0.55755556 |
| hsa-miR-5189-5p (miRBase21_mature_hsa) | -1.866 | 0.661 | 0.7511873 | -0.1485846 |
| hsa-miR-6721-5p (miRBase21_mature_hsa) | -1.582 | 0.664 | 0.69001734 | 0.02813283 |
| hsa-miR-28-3p (miRBase21_mature_hsa) | -1.371 | 0.666 | 0.044028997 | -0.41164884 |
| hsa-miR-223-5p (miRBase21_mature_hsa) | -1.343 | 0.666 | 0.12089348 | -0.30499277 |
| hsa-miR-1271-5p (miRBase21_mature_hsa) | 1.626 | 0.666 | -0.57963264 | 0.12192079 |
| hsa-miR-6842-5p (miRBase21_mature_hsa) | 1.626 | 0.666 | -1.1001551 | -0.3986017 |
| hsa-miR-136-5p (miRBase21_mature_hsa) | -1.882 | 0.673 | 0.8127932 | -0.09939749 |
| hsa-miR-221-3p (miRBase21_mature_hsa) | -1.397 | 0.677 | 0.38389543 | -0.09874423 |
| hsa-miR-659-5p (miRBase21_mature_hsa) | -1.765 | 0.678 | 0.77833885 | -0.0411706 |
| hsa-miR-30c-5p (miRBase21_mature_hsa) | -1.249 | 0.680 | 0.3607541 | 0.04037292 |
| hsa-miR-382-5p (miRBase21_mature_hsa) | -1.331 | 0.689 | 0.5369327 | 0.12424294 |
| hsa-miR-146b-5p (miRBase21_mature_hsa) | -1.256 | 0.691 | 0.029770542 | -0.2989148 |
| hsa-miR-139-5p (miRBase21_mature_hsa) | -1.331 | 0.692 | 0.19687776 | -0.21614772 |
| hsa-miR-6842-3p (miRBase21_mature_hsa) | -1.346 | 0.695 | 0.21054244 | -0.21770933 |
| hsa-miR-1291 (miRBase21_mature_hsa) | -1.467 | 0.695 | 0.7447175 | 0.1921562 |
| hsa-miR-377-5p (miRBase21_mature_hsa) | -1.467 | 0.695 | 0.9684537 | 0.41589233 |
| hsa-miR-99a-5p (miRBase21_mature_hsa) | -1.234 | 0.696 | 0.22386165 | -0.07910534 |
| hsa-miR-28-5p (miRBase21_mature_hsa) | 1.559 | 0.697 | -0.6930434 | -0.052548014 |
| hsa-miR-100-5p (miRBase21_mature_hsa) | 1.291 | 0.697 | 0.18888927 | 0.55748266 |
| hsa-miR-3127-3p (miRBase21_mature_hsa) | 1.581 | 0.698 | -0.3309836 | 0.32997093 |
| hsa-miR-148a-5p (miRBase21_mature_hsa) | 1.708 | 0.698 | -0.08827548 | 0.68374115 |
| hsa-miR-766-5p (miRBase21_mature_hsa) | -1.288 | 0.700 | 0.78318393 | 0.41798764 |
| hsa-miR-151a-3p (miRBase21_mature_hsa) | -1.424 | 0.700 | 0.12859851 | -0.38179025 |
| hsa-miR-1-3p (miRBase21_mature_hsa) | -1.748 | 0.700 | 0.8033945 | -0.002411297 |
| hsa-miR-132-5p (miRBase21_mature_hsa) | 1.518 | 0.704 | -0.15836383 | 0.44419742 |
| hsa-miR-361-3p (miRBase21_mature_hsa) | -1.288 | 0.705 | 0.89954317 | 0.53482825 |
| hsa-miR-32-5p (miRBase21_mature_hsa) | 1.675 | 0.706 | -0.29140747 | 0.45239317 |
| hsa-miR-486-3p (miRBase21_mature_hsa) | 1.218 | 0.708 | -0.19315892 | 0.09081778 |
| hsa-miR-381-3p (miRBase21_mature_hsa) | -1.395 | 0.708 | 0.7157115 | 0.23568937 |
| hsa-miR-484 (miRBase21_mature_hsa) | -1.184 | 0.711 | 0.29587373 | 0.051972322 |
| hsa-miR-361-5p (miRBase21_mature_hsa) | -1.527 | 0.717 | 0.8807754 | 0.27032962 |
| hsa-miR-3605-5p (miRBase21_mature_hsa) | 1.540 | 0.718 | -0.27563143 | 0.3475191 |
| hsa-miR-6805-5p (miRBase21_mature_hsa) | -1.299 | 0.720 | 0.66707015 | 0.2891991 |
| hsa-miR-93-5p (miRBase21_mature_hsa) | -1.294 | 0.720 | 1.0667732 | 0.6954458 |
| hsa-miR-1292-5p (miRBase21_mature_hsa) | 1.597 | 0.724 | -0.1618623 | 0.51389766 |
| hsa-miR-1299 (miRBase21_mature_hsa) | 1.503 | 0.731 | 0.025256444 | 0.61277604 |
| hsa-miR-539-5p (miRBase21_mature_hsa) | -1.683 | 0.732 | -0.7374287 | -1.4888456 |
| hsa-miR-548l (miRBase21_mature_hsa) | -1.613 | 0.739 | 0.9416364 | 0.2518951 |
| hsa-miR-10a-5p (miRBase21_mature_hsa) | 1.219 | 0.739 | -0.32206693 | -0.036468644 |
| hsa-miR-331-3p (miRBase21_mature_hsa) | 1.357 | 0.741 | -0.833909 | -0.3930075 |
| hsa-miR-4665-5p (miRBase21_mature_hsa) | -1.644 | 0.743 | 0.8848278 | 0.16718929 |
| hsa-miR-342-5p (miRBase21_mature_hsa) | 1.208 | 0.745 | -0.14123224 | 0.13178903 |
| hsa-miR-27b-3p (miRBase21_mature_hsa) | -1.284 | 0.748 | 0.018018354 | -0.34318015 |
| hsa-let-7b-5p (miRBase21_mature_hsa) | 1.173 | 0.748 | -0.13915625 | 0.09100721 |
| hsa-miR-4755-5p (miRBase21_mature_hsa) | -1.413 | 0.752 | 0.04921379 | -0.44995043 |
| hsa-miR-941 (miRBase21_mature_hsa) | -1.223 | 0.756 | 0.16237298 | -0.12835343 |
| hsa-miR-501-3p (miRBase21_mature_hsa) | 1.394 | 0.761 | -0.09952899 | 0.37960702 |
| hsa-miR-652-3p (miRBase21_mature_hsa) | -1.642 | 0.762 | -0.28954655 | -1.0049174 |
| hsa-miR-454-3p (miRBase21_mature_hsa) | -1.511 | 0.762 | 0.89810604 | 0.3022768 |
| hsa-miR-129-5p (miRBase21_mature_hsa) | 1.407 | 0.762 | 0.73685163 | 1.2295251 |
| hsa-miR-6873-3p (miRBase21_mature_hsa) | -1.378 | 0.766 | 0.9000306 | 0.437039 |
| hsa-let-7i-5p (miRBase21_mature_hsa) | -1.266 | 0.768 | 0.2890082 | -0.050932895 |
| hsa-miR-3074-5p (miRBase21_mature_hsa) | -1.260 | 0.768 | -0.083108164 | -0.4167204 |
| hsa-miR-184 (miRBase21_mature_hsa) | 1.364 | 0.771 | -0.21018508 | 0.23776256 |
| hsa-miR-221-5p (miRBase21_mature_hsa) | -1.577 | 0.771 | 0.8493251 | 0.19200721 |
| hsa-miR-3127-5p (miRBase21_mature_hsa) | -1.282 | 0.779 | 0.25653848 | -0.10206331 |
| hsa-miR-425-5p (miRBase21_mature_hsa) | -1.120 | 0.780 | 0.36058462 | 0.19687805 |
| hsa-miR-200a-5p (miRBase21_mature_hsa) | -1.323 | 0.782 | 0.33566895 | -0.06817511 |
| hsa-miR-3124-5p (miRBase21_mature_hsa) | -1.323 | 0.782 | 0.75770265 | 0.35385862 |

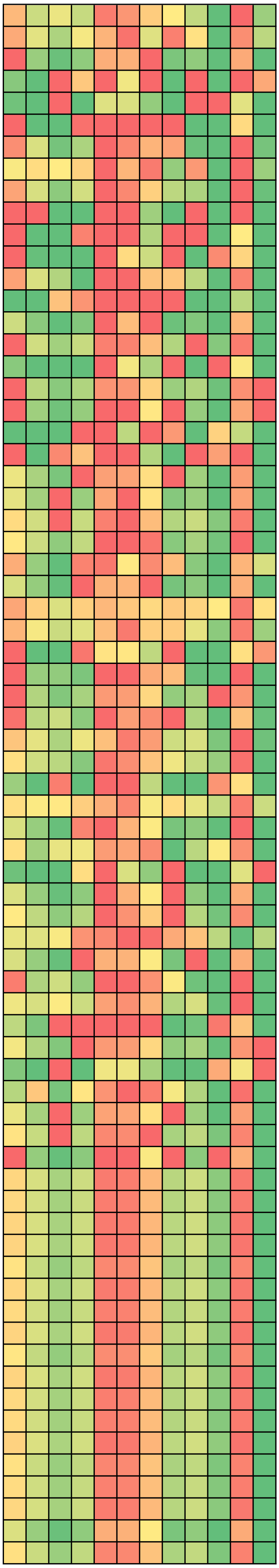

| | | | | |
|---|---|---|---|---|
| hsa-miR-191-5p (miRBase21_mature_hsa) | -1.151 | 0.783 | 0.16684234 | -0.036643505 |
| hsa-miR-532-5p (miRBase21_mature_hsa) | 1.141 | 0.783 | 0.07307255 | 0.26289976 |
| hsa-miR-6859-5p (miRBase21_mature_hsa) | -1.346 | 0.785 | 0.13121462 | -0.29761893 |
| hsa-miR-6738-3p (miRBase21_mature_hsa) | 1.437 | 0.786 | 0.73632 | 1.2595426 |
| hsa-miR-3064-5p (miRBase21_mature_hsa) | 1.291 | 0.787 | -1.1113691 | -0.74233174 |
| hsa-miR-196b-5p (miRBase21_mature_hsa) | -1.471 | 0.788 | 1.0341283 | 0.4772134 |
| hsa-miR-148b-3p (miRBase21_mature_hsa) | -1.232 | 0.792 | 0.17908254 | -0.12221775 |
| hsa-miR-342-3p (miRBase21_mature_hsa) | -1.170 | 0.793 | 0.5325 | 0.30660442 |
| hsa-miR-7-5p (miRBase21_mature_hsa) | -1.204 | 0.800 | 0.17156485 | -0.09663813 |
| hsa-miR-1255a (miRBase21_mature_hsa) | -1.553 | 0.804 | 1.2617922 | 0.6265719 |
| hsa-miR-125a-3p (miRBase21_mature_hsa) | -1.396 | 0.807 | 0.77860546 | 0.2975381 |
| hsa-miR-503-5p (miRBase21_mature_hsa) | -1.328 | 0.808 | 0.10974188 | -0.2994115 |
| hsa-miR-335-3p (miRBase21_mature_hsa) | -1.262 | 0.809 | 0.16255358 | -0.17283542 |
| hsa-miR-421 (miRBase21_mature_hsa) | -1.376 | 0.809 | -0.054208897 | -0.5150274 |
| hsa-miR-548u (miRBase21_mature_hsa) | -1.345 | 0.811 | -0.061009537 | -0.48893297 |
| hsa-miR-4645-3p (miRBase21_mature_hsa) | -1.277 | 0.814 | 0.8281621 | 0.47517598 |
| hsa-miR-3120-3p (miRBase21_mature_hsa) | 1.343 | 0.816 | -0.84436023 | -0.41921896 |
| hsa-miR-5587-3p (miRBase21_mature_hsa) | -1.245 | 0.816 | 0.531639 | 0.21583812 |
| hsa-miR-548j-3p (miRBase21_mature_hsa) | 1.337 | 0.817 | 0.830605 | 1.2497916 |
| hsa-miR-23b-3p (miRBase21_mature_hsa) | -1.438 | 0.818 | 0.001667547 | -0.5225416 |
| hsa-miR-4664-3p (miRBase21_mature_hsa) | -1.365 | 0.822 | 1.5818516 | 1.1327351 |
| hsa-miR-29c-5p (miRBase21_mature_hsa) | -1.198 | 0.825 | 0.19412775 | -0.0670617 |
| hsa-miR-4742-3p (miRBase21_mature_hsa) | -1.237 | 0.825 | 0.18582368 | -0.12137781 |
| hsa-miR-642a-5p (miRBase21_mature_hsa) | -1.237 | 0.825 | 0.76928437 | 0.4620829 |
| hsa-miR-148a-3p (miRBase21_mature_hsa) | -1.212 | 0.827 | 0.052341 | -0.22503726 |
| hsa-miR-143-3p (miRBase21_mature_hsa) | -1.167 | 0.828 | 0.093540624 | -0.12918566 |
| hsa-miR-6855-5p (miRBase21_mature_hsa) | -1.219 | 0.828 | -0.07907756 | -0.36525652 |
| hsa-miR-22-3p (miRBase21_mature_hsa) | 1.054 | 0.829 | 0.44963512 | 0.52505875 |
| hsa-miR-92a-3p (miRBase21_mature_hsa) | 1.091 | 0.830 | 0.07003015 | 0.19593884 |
| hsa-miR-18a-5p (miRBase21_mature_hsa) | -1.239 | 0.832 | 0.1134096 | -0.19583271 |
| hsa-let-7f-5p (miRBase21_mature_hsa) | -1.222 | 0.832 | 0.18422763 | -0.10551931 |
| hsa-miR-3679-5p (miRBase21_mature_hsa) | -1.236 | 0.833 | 0.43738014 | 0.13213442 |
| hsa-miR-584-5p (miRBase21_mature_hsa) | -1.162 | 0.839 | 0.17333356 | -0.043268196 |
| hsa-miR-423-5p (miRBase21_mature_hsa) | 1.105 | 0.839 | -0.06932706 | 0.07500413 |
| hsa-miR-185-5p (miRBase21_mature_hsa) | 1.120 | 0.839 | -0.20342636 | -0.040013857 |
| hsa-miR-15b-3p (miRBase21_mature_hsa) | -1.242 | 0.847 | -0.12150569 | -0.4340164 |
| hsa-miR-186-5p (miRBase21_mature_hsa) | -1.058 | 0.849 | 0.3256525 | 0.24378304 |
| hsa-miR-1287-5p (miRBase21_mature_hsa) | -1.272 | 0.851 | 0.3784406 | 0.031354032 |
| hsa-miR-4433b-5p (miRBase21_mature_hsa) | -1.100 | 0.852 | -0.04860241 | -0.18624008 |
| hsa-miR-335-5p (miRBase21_mature_hsa) | 1.249 | 0.856 | -0.6549294 | -0.3346901 |
| hsa-miR-3928-3p (miRBase21_mature_hsa) | -1.219 | 0.857 | -0.009528363 | -0.29546982 |
| hsa-miR-651-5p (miRBase21_mature_hsa) | -1.219 | 0.857 | 0.48853204 | 0.2025905 |
| hsa-miR-433-3p (miRBase21_mature_hsa) | -1.125 | 0.864 | 0.42100894 | 0.25069743 |
| hsa-miR-3173-5p (miRBase21_mature_hsa) | -1.157 | 0.864 | -0.053970553 | -0.26430207 |
| hsa-let-7a-5p (miRBase21_mature_hsa) | -1.152 | 0.865 | 0.166679 | -0.03787629 |
| hsa-miR-320a (miRBase21_mature_hsa) | -1.094 | 0.866 | -0.047738582 | -0.17673849 |
| hsa-miR-3913-5p (miRBase21_mature_hsa) | -1.235 | 0.867 | 1.2383085 | 0.93323135 |
| hsa-miR-1227-3p (miRBase21_mature_hsa) | -1.128 | 0.869 | 0.29071254 | 0.11756627 |
| hsa-miR-5193 (miRBase21_mature_hsa) | 1.153 | 0.870 | -0.7607862 | -0.5551222 |
| hsa-miR-485-5p (miRBase21_mature_hsa) | 1.116 | 0.870 | -0.12755293 | 0.03128852 |
| hsa-miR-6787-3p (miRBase21_mature_hsa) | -1.141 | 0.874 | 0.2323184 | 0.041563306 |
| hsa-miR-6821-5p (miRBase21_mature_hsa) | -1.161 | 0.875 | 0.6423987 | 0.42665407 |
| hsa-miR-548e-3p (miRBase21_mature_hsa) | 1.220 | 0.877 | 0.70409346 | 0.9909782 |
| hsa-let-7c-3p (miRBase21_mature_hsa) | -1.090 | 0.877 | -0.03922514 | -0.164116 |
| hsa-let-7f-2-3p (miRBase21_mature_hsa) | -1.090 | 0.877 | -0.07404829 | -0.19893916 |
| hsa-miR-10b-3p (miRBase21_mature_hsa) | -1.090 | 0.877 | -0.029688925 | -0.15457979 |
| hsa-miR-122-3p (miRBase21_mature_hsa) | -1.090 | 0.877 | -0.012009049 | -0.13689993 |
| hsa-miR-1224-5p (miRBase21_mature_hsa) | -1.090 | 0.877 | -0.23716438 | -0.36205527 |
| hsa-miR-1228-3p (miRBase21_mature_hsa) | -1.090 | 0.877 | -0.038684875 | -0.16357574 |
| hsa-miR-1234-3p (miRBase21_mature_hsa) | -1.090 | 0.877 | -0.108050436 | -0.2329413 |
| hsa-miR-1236-5p (miRBase21_mature_hsa) | -1.090 | 0.877 | -0.006507122 | -0.131398 |
| hsa-miR-1254 (miRBase21_mature_hsa) | -1.090 | 0.877 | -0.29264614 | -0.41753703 |
| hsa-miR-1256 (miRBase21_mature_hsa) | -1.090 | 0.877 | -0.006716645 | -0.13160752 |
| hsa-miR-1258 (miRBase21_mature_hsa) | -1.090 | 0.877 | -0.08069893 | -0.20558982 |
| hsa-miR-1260a (miRBase21_mature_hsa) | -1.090 | 0.877 | -0.105563045 | -0.2304539 |
| hsa-miR-1269b (miRBase21_mature_hsa) | -1.090 | 0.877 | 0.01813544 | -0.10675543 |
| hsa-miR-1273c (miRBase21_mature_hsa) | -1.090 | 0.877 | -0.21313775 | -0.3380286 |
| hsa-miR-1273d (miRBase21_mature_hsa) | -1.090 | 0.877 | -0.13443288 | -0.25932375 |
| hsa-miR-1277-3p (miRBase21_mature_hsa) | -1.090 | 0.877 | -0.060063213 | -0.18495408 |
| hsa-miR-1284 (miRBase21_mature_hsa) | -1.090 | 0.877 | -0.8258922 | -0.95078313 |
| hsa-miR-1303 (miRBase21_mature_hsa) | -1.090 | 0.877 | -0.19894063 | -0.3238315 |

| | | | | |
|---|---|---|---|---|
| hsa-miR-1306-3p (miRBase21_mature_hsa) | -1.090 | 0.877 | -0.035508513 | -0.16039936 |
| hsa-miR-15a-5p (miRBase21_mature_hsa) | -1.090 | 0.877 | 1.44E-04 | -0.12474734 |
| hsa-miR-17-3p (miRBase21_mature_hsa) | -1.090 | 0.877 | 1.44E-04 | -0.12474734 |
| hsa-miR-181c-5p (miRBase21_mature_hsa) | -1.090 | 0.877 | 1.44E-04 | -0.12474734 |
| hsa-miR-1827 (miRBase21_mature_hsa) | -1.090 | 0.877 | 0.09232725 | -0.032563616 |
| hsa-miR-187-3p (miRBase21_mature_hsa) | -1.090 | 0.877 | -0.00695957 | -0.13185045 |
| hsa-miR-190b (miRBase21_mature_hsa) | -1.090 | 0.877 | -0.10206196 | -0.22695284 |
| hsa-miR-193b-3p (miRBase21_mature_hsa) | -1.090 | 0.877 | -0.006716645 | -0.13160752 |
| hsa-miR-196a-5p (miRBase21_mature_hsa) | -1.090 | 0.877 | -0.16016486 | -0.28505573 |
| hsa-miR-19b-1-5p (miRBase21_mature_hsa) | -1.090 | 0.877 | -0.013206327 | -0.1380972 |
| hsa-miR-202-3p (miRBase21_mature_hsa) | -1.090 | 0.877 | -0.007408142 | -0.13229902 |
| hsa-miR-202-5p (miRBase21_mature_hsa) | -1.090 | 0.877 | -0.007408142 | -0.13229902 |
| hsa-miR-210-5p (miRBase21_mature_hsa) | -1.090 | 0.877 | -0.007408142 | -0.13229902 |
| hsa-miR-214-3p (miRBase21_mature_hsa) | -1.090 | 0.877 | -0.124748826 | -0.24963968 |
| hsa-miR-216b-5p (miRBase21_mature_hsa) | -1.090 | 0.877 | -0.11225022 | -0.2371411 |
| hsa-miR-217 (miRBase21_mature_hsa) | -1.090 | 0.877 | 0.01813544 | -0.10675543 |
| hsa-miR-219a-1-3p (miRBase21_mature_hsa) | -1.090 | 0.877 | -0.18466592 | -0.30955678 |
| hsa-miR-2276-3p (miRBase21_mature_hsa) | -1.090 | 0.877 | -0.0113796 | -0.1362705 |
| hsa-miR-2277-5p (miRBase21_mature_hsa) | -1.090 | 0.877 | -0.32621756 | -0.45110843 |
| hsa-miR-2355-3p (miRBase21_mature_hsa) | -1.090 | 0.877 | -0.71037865 | -0.8352695 |
| hsa-miR-2467-5p (miRBase21_mature_hsa) | -1.090 | 0.877 | -0.023064721 | -0.14795558 |
| hsa-miR-26a-1-3p (miRBase21_mature_hsa) | -1.090 | 0.877 | -0.08470575 | -0.20959662 |
| hsa-miR-26a-2-3p (miRBase21_mature_hsa) | -1.090 | 0.877 | 4.94E-04 | -0.12439718 |
| hsa-miR-296-3p (miRBase21_mature_hsa) | -1.090 | 0.877 | -0.006716645 | -0.13160752 |
| hsa-miR-296-5p (miRBase21_mature_hsa) | -1.090 | 0.877 | -0.49010074 | -0.6149916 |
| hsa-miR-29c-3p (miRBase21_mature_hsa) | -1.090 | 0.877 | -0.019989992 | -0.14488086 |
| hsa-miR-301b-3p (miRBase21_mature_hsa) | -1.090 | 0.877 | -0.27523097 | -0.40012184 |
| hsa-miR-301b-5p (miRBase21_mature_hsa) | -1.090 | 0.877 | -0.035508513 | -0.16039936 |
| hsa-miR-3074-3p (miRBase21_mature_hsa) | -1.090 | 0.877 | -0.00695957 | -0.13185045 |
| hsa-miR-3133 (miRBase21_mature_hsa) | -1.090 | 0.877 | 0.01412859 | -0.11076226 |
| hsa-miR-3138 (miRBase21_mature_hsa) | -1.090 | 0.877 | 0.01813544 | -0.10675543 |
| hsa-miR-3140-3p (miRBase21_mature_hsa) | -1.090 | 0.877 | -0.08159995 | -0.20649083 |
| hsa-miR-3143 (miRBase21_mature_hsa) | -1.090 | 0.877 | -0.012009049 | -0.13689993 |
| hsa-miR-3144-5p (miRBase21_mature_hsa) | -1.090 | 0.877 | 0.01813544 | -0.10675543 |
| hsa-miR-3146 (miRBase21_mature_hsa) | -1.090 | 0.877 | 0.00243547 | -0.1224554 |
| hsa-miR-3149 (miRBase21_mature_hsa) | -1.090 | 0.877 | 0.01813544 | -0.10675543 |
| hsa-miR-3157-5p (miRBase21_mature_hsa) | -1.090 | 0.877 | -0.012009049 | -0.13689993 |
| hsa-miR-3159 (miRBase21_mature_hsa) | -1.090 | 0.877 | -0.012170196 | -0.13706104 |
| hsa-miR-3168 (miRBase21_mature_hsa) | -1.090 | 0.877 | 4.94E-04 | -0.12439718 |
| hsa-miR-3174 (miRBase21_mature_hsa) | -1.090 | 0.877 | -0.43080568 | -0.55569655 |
| hsa-miR-3179 (miRBase21_mature_hsa) | -1.090 | 0.877 | 0.01813544 | -0.10675543 |
| hsa-miR-3180 (miRBase21_mature_hsa) | -1.090 | 0.877 | 0.09232725 | -0.032563616 |
| hsa-miR-3180-3p (miRBase21_mature_hsa) | -1.090 | 0.877 | -0.24925613 | -0.374147 |
| hsa-miR-3180-5p (miRBase21_mature_hsa) | -1.090 | 0.877 | 0.01412859 | -0.11076226 |
| hsa-miR-3184-5p (miRBase21_mature_hsa) | -1.090 | 0.877 | 1.44E-04 | -0.12474734 |
| hsa-miR-3192-5p (miRBase21_mature_hsa) | -1.090 | 0.877 | -0.006507122 | -0.131398 |
| hsa-miR-3198 (miRBase21_mature_hsa) | -1.090 | 0.877 | -0.43334135 | -0.55823225 |
| hsa-miR-3199 (miRBase21_mature_hsa) | -1.090 | 0.877 | -0.106951825 | -0.23184268 |
| hsa-miR-320e (miRBase21_mature_hsa) | -1.090 | 0.877 | 1.44E-04 | -0.12474734 |
| hsa-miR-32-3p (miRBase21_mature_hsa) | -1.090 | 0.877 | -0.6241795 | -0.7490704 |
| hsa-miR-329-5p (miRBase21_mature_hsa) | -1.090 | 0.877 | -0.7583041 | -0.883195 |
| hsa-miR-33a-5p (miRBase21_mature_hsa) | -1.090 | 0.877 | -0.10704759 | -0.23193845 |
| hsa-miR-3591-5p (miRBase21_mature_hsa) | -1.090 | 0.877 | -0.19096088 | -0.31585175 |
| hsa-miR-3614-5p (miRBase21_mature_hsa) | -1.090 | 0.877 | -0.18768331 | -0.3125742 |
| hsa-miR-365a-3p (miRBase21_mature_hsa) | -1.090 | 0.877 | -0.13021243 | -0.25510332 |
| hsa-miR-3675-3p (miRBase21_mature_hsa) | -1.090 | 0.877 | 4.94E-04 | -0.12439718 |
| hsa-miR-3678-5p (miRBase21_mature_hsa) | -1.090 | 0.877 | 0.001749134 | -0.12314176 |
| hsa-miR-3682-3p (miRBase21_mature_hsa) | -1.090 | 0.877 | -0.110423535 | -0.23531441 |
| hsa-miR-3687 (miRBase21_mature_hsa) | -1.090 | 0.877 | 1.44E-04 | -0.12474734 |
| hsa-miR-372-3p (miRBase21_mature_hsa) | -1.090 | 0.877 | -0.007949794 | -0.13284068 |
| hsa-miR-378a-5p (miRBase21_mature_hsa) | -1.090 | 0.877 | -0.021657933 | -0.14654882 |
| hsa-miR-378e (miRBase21_mature_hsa) | -1.090 | 0.877 | 0.09232725 | -0.032563616 |
| hsa-miR-378g (miRBase21_mature_hsa) | -1.090 | 0.877 | 0.01813544 | -0.10675543 |
| hsa-miR-380-5p (miRBase21_mature_hsa) | -1.090 | 0.877 | 0.001749134 | -0.12314176 |
| hsa-miR-383-5p (miRBase21_mature_hsa) | -1.090 | 0.877 | -0.035508513 | -0.16039936 |
| hsa-miR-3912-3p (miRBase21_mature_hsa) | -1.090 | 0.877 | 0.01813544 | -0.10675543 |
| hsa-miR-3918 (miRBase21_mature_hsa) | -1.090 | 0.877 | 0.01813544 | -0.10675543 |
| hsa-miR-3939 (miRBase21_mature_hsa) | -1.090 | 0.877 | -0.00695957 | -0.13185045 |
| hsa-miR-3940-3p (miRBase21_mature_hsa) | -1.090 | 0.877 | 0.01813544 | -0.10675543 |
| hsa-miR-410-5p (miRBase21_mature_hsa) | -1.090 | 0.877 | 0.003950024 | -0.12094083 |
| hsa-miR-422a (miRBase21_mature_hsa) | -1.090 | 0.877 | 0.01813544 | -0.10675543 |

| | | | | |
|---|---|---|---|---|
| hsa-miR-4440 (miRBase21_mature_hsa) | -1.090 | 0.877 | -0.029935395 | -0.15482625 |
| hsa-miR-4446-5p (miRBase21_mature_hsa) | -1.090 | 0.877 | 0.01412859 | -0.11076226 |
| hsa-miR-4474-3p (miRBase21_mature_hsa) | -1.090 | 0.877 | 0.01412859 | -0.11076226 |
| hsa-miR-4485-3p (miRBase21_mature_hsa) | -1.090 | 0.877 | -0.027923971 | -0.15281485 |
| hsa-miR-4488 (miRBase21_mature_hsa) | -1.090 | 0.877 | -0.12609425 | -0.25098515 |
| hsa-miR-4489 (miRBase21_mature_hsa) | -1.090 | 0.877 | -0.23919305 | -0.36408392 |
| hsa-miR-449c-5p (miRBase21_mature_hsa) | -1.090 | 0.877 | 1.44E-04 | -0.12474734 |
| hsa-miR-450a-2-3p (miRBase21_mature_hsa) | -1.090 | 0.877 | -0.033118732 | -0.1580096 |
| hsa-miR-4511 (miRBase21_mature_hsa) | -1.090 | 0.877 | -0.09008444 | -0.21497533 |
| hsa-miR-4533 (miRBase21_mature_hsa) | -1.090 | 0.877 | 0.00243547 | -0.1224554 |
| hsa-miR-4632-5p (miRBase21_mature_hsa) | -1.090 | 0.877 | -0.007853997 | -0.13274486 |
| hsa-miR-4635 (miRBase21_mature_hsa) | -1.090 | 0.877 | -0.007408142 | -0.13229902 |
| hsa-miR-4646-5p (miRBase21_mature_hsa) | -1.090 | 0.877 | -0.09753772 | -0.2224286 |
| hsa-miR-4648 (miRBase21_mature_hsa) | -1.090 | 0.877 | -0.007408142 | -0.13229902 |
| hsa-miR-4657 (miRBase21_mature_hsa) | -1.090 | 0.877 | -0.10264758 | -0.22753844 |
| hsa-miR-4658 (miRBase21_mature_hsa) | -1.090 | 0.877 | -0.021657933 | -0.14654882 |
| hsa-miR-4662a-5p (miRBase21_mature_hsa) | -1.090 | 0.877 | -0.22361293 | -0.3485038 |
| hsa-miR-4677-5p (miRBase21_mature_hsa) | -1.090 | 0.877 | 0.01412859 | -0.11076226 |
| hsa-miR-4697-3p (miRBase21_mature_hsa) | -1.090 | 0.877 | 0.001749134 | -0.12314176 |
| hsa-miR-4708-3p (miRBase21_mature_hsa) | -1.090 | 0.877 | 0.01813544 | -0.10675543 |
| hsa-miR-4709-3p (miRBase21_mature_hsa) | -1.090 | 0.877 | -0.16988873 | -0.2947796 |
| hsa-miR-4717-3p (miRBase21_mature_hsa) | -1.090 | 0.877 | 0.003950024 | -0.12094083 |
| hsa-miR-4721 (miRBase21_mature_hsa) | -1.090 | 0.877 | -0.021657933 | -0.14654882 |
| hsa-miR-4722-5p (miRBase21_mature_hsa) | -1.090 | 0.877 | 0.00491786 | -0.11997301 |
| hsa-miR-4723-3p (miRBase21_mature_hsa) | -1.090 | 0.877 | -0.11348334 | -0.23837422 |
| hsa-miR-4738-3p (miRBase21_mature_hsa) | -1.090 | 0.877 | -0.08726588 | -0.21215676 |
| hsa-miR-4754 (miRBase21_mature_hsa) | -1.090 | 0.877 | 0.01412859 | -0.11076226 |
| hsa-miR-4765 (miRBase21_mature_hsa) | -1.090 | 0.877 | -0.09526339 | -0.22015427 |
| hsa-miR-4775 (miRBase21_mature_hsa) | -1.090 | 0.877 | 0.01813544 | -0.10675543 |
| hsa-miR-4779 (miRBase21_mature_hsa) | -1.090 | 0.877 | 0.01412859 | -0.11076226 |
| hsa-miR-4796-3p (miRBase21_mature_hsa) | -1.090 | 0.877 | -0.016821265 | -0.14171216 |
| hsa-miR-4800-5p (miRBase21_mature_hsa) | -1.090 | 0.877 | -0.007408142 | -0.13229902 |
| hsa-miR-483-3p (miRBase21_mature_hsa) | -1.090 | 0.877 | -0.40534854 | -0.5302394 |
| hsa-miR-490-5p (miRBase21_mature_hsa) | -1.090 | 0.877 | -0.007949794 | -0.13284068 |
| hsa-miR-491-5p (miRBase21_mature_hsa) | -1.090 | 0.877 | -0.029935395 | -0.15482625 |
| hsa-miR-5001-3p (miRBase21_mature_hsa) | -1.090 | 0.877 | -0.012497556 | -0.13738844 |
| hsa-miR-5001-5p (miRBase21_mature_hsa) | -1.090 | 0.877 | -0.09349546 | -0.21838632 |
| hsa-miR-5004-5p (miRBase21_mature_hsa) | -1.090 | 0.877 | -0.021657933 | -0.14654882 |
| hsa-miR-5006-3p (miRBase21_mature_hsa) | -1.090 | 0.877 | -0.023064721 | -0.14795558 |
| hsa-miR-5009-5p (miRBase21_mature_hsa) | -1.090 | 0.877 | -0.4590366 | -0.58392745 |
| hsa-miR-500a-5p (miRBase21_mature_hsa) | -1.090 | 0.877 | 0.01813544 | -0.10675543 |
| hsa-miR-500b-3p (miRBase21_mature_hsa) | -1.090 | 0.877 | 1.44E-04 | -0.12474734 |
| hsa-miR-501-5p (miRBase21_mature_hsa) | -1.090 | 0.877 | -0.029935395 | -0.15482625 |
| hsa-miR-504-3p (miRBase21_mature_hsa) | -1.090 | 0.877 | -0.04480385 | -0.16969474 |
| hsa-miR-505-3p (miRBase21_mature_hsa) | -1.090 | 0.877 | -0.23130417 | -0.35619503 |
| hsa-miR-506-3p (miRBase21_mature_hsa) | -1.090 | 0.877 | 4.94E-04 | -0.12439718 |
| hsa-miR-508-3p (miRBase21_mature_hsa) | -1.090 | 0.877 | -0.0113796 | -0.1362705 |
| hsa-miR-509-3-5p (miRBase21_mature_hsa) | -1.090 | 0.877 | -0.2226286 | -0.34751946 |
| hsa-miR-514a-3p (miRBase21_mature_hsa) | -1.090 | 0.877 | -2.95E-05 | -0.12492036 |
| hsa-miR-517a-3p (miRBase21_mature_hsa) | -1.090 | 0.877 | -0.021657933 | -0.14654882 |
| hsa-miR-517b-3p (miRBase21_mature_hsa) | -1.090 | 0.877 | 0.09232725 | -0.032563616 |
| hsa-miR-518e-5p (miRBase21_mature_hsa) | -1.090 | 0.877 | 0.09232725 | -0.032563616 |
| hsa-miR-5190 (miRBase21_mature_hsa) | -1.090 | 0.877 | -0.0113796 | -0.1362705 |
| hsa-miR-519a-5p (miRBase21_mature_hsa) | -1.090 | 0.877 | 0.09232725 | -0.032563616 |
| hsa-miR-519b-5p (miRBase21_mature_hsa) | -1.090 | 0.877 | 0.09232725 | -0.032563616 |
| hsa-miR-519c-5p (miRBase21_mature_hsa) | -1.090 | 0.877 | 0.09232725 | -0.032563616 |
| hsa-miR-523-5p (miRBase21_mature_hsa) | -1.090 | 0.877 | 0.09232725 | -0.032563616 |
| hsa-miR-539-3p (miRBase21_mature_hsa) | -1.090 | 0.877 | -0.035508513 | -0.16039936 |
| hsa-miR-542-3p (miRBase21_mature_hsa) | -1.090 | 0.877 | -0.091980904 | -0.21687178 |
| hsa-miR-548aa (miRBase21_mature_hsa) | -1.090 | 0.877 | 0.09232725 | -0.032563616 |
| hsa-miR-548ad-5p (miRBase21_mature_hsa) | -1.090 | 0.877 | 0.09232725 | -0.032563616 |
| hsa-miR-548ae-5p (miRBase21_mature_hsa) | -1.090 | 0.877 | -0.0113796 | -0.1362705 |
| hsa-miR-548ag (miRBase21_mature_hsa) | -1.090 | 0.877 | 0.00243547 | -0.1224554 |
| hsa-miR-548ay-5p (miRBase21_mature_hsa) | -1.090 | 0.877 | 0.09232725 | -0.032563616 |
| hsa-miR-548g-3p (miRBase21_mature_hsa) | -1.090 | 0.877 | -0.080514394 | -0.20540528 |
| hsa-miR-548k (miRBase21_mature_hsa) | -1.090 | 0.877 | -0.101263374 | -0.22615424 |
| hsa-miR-548n (miRBase21_mature_hsa) | -1.090 | 0.877 | -2.95E-05 | -0.12492036 |
| hsa-miR-548t-3p (miRBase21_mature_hsa) | -1.090 | 0.877 | 1.44E-04 | -0.12474734 |
| hsa-miR-550a-3p (miRBase21_mature_hsa) | -1.090 | 0.877 | 4.94E-04 | -0.12439718 |
| hsa-miR-550b-2-5p (miRBase21_mature_hsa) | -1.090 | 0.877 | -2.95E-05 | -0.12492036 |
| hsa-miR-551b-3p (miRBase21_mature_hsa) | -1.090 | 0.877 | 4.94E-04 | -0.12439718 |

| | | | | |
|---|---|---|---|---|
| hsa-miR-551b-5p (miRBase21_mature_hsa) | -1.090 | 0.877 | -0.023064721 | -0.14795558 |
| hsa-miR-5589-5p (miRBase21_mature_hsa) | -1.090 | 0.877 | -0.012170196 | -0.13706104 |
| hsa-miR-581 (miRBase21_mature_hsa) | -1.090 | 0.877 | -0.1970326 | -0.32192346 |
| hsa-miR-605-5p (miRBase21_mature_hsa) | -1.090 | 0.877 | 1.44E-04 | -0.12474734 |
| hsa-miR-6087 (miRBase21_mature_hsa) | -1.090 | 0.877 | -0.14104208 | -0.26593295 |
| hsa-miR-6130 (miRBase21_mature_hsa) | -1.090 | 0.877 | 0.09232725 | -0.032563616 |
| hsa-miR-616-3p (miRBase21_mature_hsa) | -1.090 | 0.877 | -0.17269814 | -0.297589 |
| hsa-miR-619-5p (miRBase21_mature_hsa) | -1.090 | 0.877 | -0.023064721 | -0.14795558 |
| hsa-miR-629-3p (miRBase21_mature_hsa) | -1.090 | 0.877 | -0.1347934 | -0.2596843 |
| hsa-miR-636 (miRBase21_mature_hsa) | -1.090 | 0.877 | -0.33175364 | -0.4566445 |
| hsa-miR-6502-5p (miRBase21_mature_hsa) | -1.090 | 0.877 | -0.023064721 | -0.14795558 |
| hsa-miR-6510-3p (miRBase21_mature_hsa) | -1.090 | 0.877 | -0.006716645 | -0.13160752 |
| hsa-miR-6511b-3p (miRBase21_mature_hsa) | -1.090 | 0.877 | -0.34311855 | -0.4680094 |
| hsa-miR-6516-3p (miRBase21_mature_hsa) | -1.090 | 0.877 | -0.11299777 | -0.23788865 |
| hsa-miR-656-3p (miRBase21_mature_hsa) | -1.090 | 0.877 | -0.0821416 | -0.20703249 |
| hsa-miR-6716-3p (miRBase21_mature_hsa) | -1.090 | 0.877 | 0.00491786 | -0.11997301 |
| hsa-miR-6726-5p (miRBase21_mature_hsa) | -1.090 | 0.877 | -0.023064721 | -0.14795558 |
| hsa-miR-6727-5p (miRBase21_mature_hsa) | -1.090 | 0.877 | -0.22059038 | -0.34548125 |
| hsa-miR-6737-3p (miRBase21_mature_hsa) | -1.090 | 0.877 | -0.110423535 | -0.23531441 |
| hsa-miR-6747-5p (miRBase21_mature_hsa) | -1.090 | 0.877 | -0.038684875 | -0.16357574 |
| hsa-miR-6754-3p (miRBase21_mature_hsa) | -1.090 | 0.877 | -0.029935395 | -0.15482625 |
| hsa-miR-6755-5p (miRBase21_mature_hsa) | -1.090 | 0.877 | -0.012009049 | -0.13689993 |
| hsa-miR-6757-5p (miRBase21_mature_hsa) | -1.090 | 0.877 | -0.12341657 | -0.24830745 |
| hsa-miR-676-3p (miRBase21_mature_hsa) | -1.090 | 0.877 | -2.95E-05 | -0.12492036 |
| hsa-miR-6770-3p (miRBase21_mature_hsa) | -1.090 | 0.877 | -0.19449985 | -0.3193907 |
| hsa-miR-6772-5p (miRBase21_mature_hsa) | -1.090 | 0.877 | -0.20460698 | -0.32949787 |
| hsa-miR-6777-5p (miRBase21_mature_hsa) | -1.090 | 0.877 | -0.049663097 | -0.17455396 |
| hsa-miR-6783-3p (miRBase21_mature_hsa) | -1.090 | 0.877 | -0.029147267 | -0.15403812 |
| hsa-miR-6784-3p (miRBase21_mature_hsa) | -1.090 | 0.877 | -0.021657933 | -0.14654882 |
| hsa-miR-6791-5p (miRBase21_mature_hsa) | -1.090 | 0.877 | -0.20525053 | -0.3301414 |
| hsa-miR-6793-3p (miRBase21_mature_hsa) | -1.090 | 0.877 | -0.006507122 | -0.131398 |
| hsa-miR-6796-5p (miRBase21_mature_hsa) | -1.090 | 0.877 | -0.006716645 | -0.13160752 |
| hsa-miR-6802-5p (miRBase21_mature_hsa) | -1.090 | 0.877 | -0.1028905 | -0.22778137 |
| hsa-miR-6804-3p (miRBase21_mature_hsa) | -1.090 | 0.877 | -2.95E-05 | -0.12492036 |
| hsa-miR-6808-3p (miRBase21_mature_hsa) | -1.090 | 0.877 | -0.20893869 | -0.33382955 |
| hsa-miR-6809-3p (miRBase21_mature_hsa) | -1.090 | 0.877 | -0.11299201 | -0.2378829 |
| hsa-miR-6810-3p (miRBase21_mature_hsa) | -1.090 | 0.877 | -0.18466926 | -0.30956012 |
| hsa-miR-6810-5p (miRBase21_mature_hsa) | -1.090 | 0.877 | -0.09957957 | -0.22447045 |
| hsa-miR-6812-3p (miRBase21_mature_hsa) | -1.090 | 0.877 | -0.2625166 | -0.38740745 |
| hsa-miR-6813-5p (miRBase21_mature_hsa) | -1.090 | 0.877 | -0.44814503 | -0.5730359 |
| hsa-miR-6815-5p (miRBase21_mature_hsa) | -1.090 | 0.877 | 0.001749134 | -0.12314176 |
| hsa-miR-6825-5p (miRBase21_mature_hsa) | -1.090 | 0.877 | -0.006716645 | -0.13160752 |
| hsa-miR-6853-3p (miRBase21_mature_hsa) | -1.090 | 0.877 | -0.10127139 | -0.22616227 |
| hsa-miR-6855-3p (miRBase21_mature_hsa) | -1.090 | 0.877 | -0.4244137 | -0.54930454 |
| hsa-miR-6861-5p (miRBase21_mature_hsa) | -1.090 | 0.877 | -0.19940433 | -0.3242952 |
| hsa-miR-6867-5p (miRBase21_mature_hsa) | -1.090 | 0.877 | 0.01813544 | -0.10675543 |
| hsa-miR-6869-5p (miRBase21_mature_hsa) | -1.090 | 0.877 | -0.03922514 | -0.164116 |
| hsa-miR-6876-5p (miRBase21_mature_hsa) | -1.090 | 0.877 | 0.00243547 | -0.1224554 |
| hsa-miR-6878-5p (miRBase21_mature_hsa) | -1.090 | 0.877 | -2.95E-05 | -0.12492036 |
| hsa-miR-6882-3p (miRBase21_mature_hsa) | -1.090 | 0.877 | -0.027923971 | -0.15281485 |
| hsa-miR-6882-5p (miRBase21_mature_hsa) | -1.090 | 0.877 | -0.08957827 | -0.21446912 |
| hsa-miR-6884-5p (miRBase21_mature_hsa) | -1.090 | 0.877 | -0.14453924 | -0.2694301 |
| hsa-miR-6891-5p (miRBase21_mature_hsa) | -1.090 | 0.877 | -0.035508513 | -0.16039936 |
| hsa-miR-7110-5p (miRBase21_mature_hsa) | -1.090 | 0.877 | 0.00243547 | -0.1224554 |
| hsa-miR-7111-3p (miRBase21_mature_hsa) | -1.090 | 0.877 | -0.21498382 | -0.3398747 |
| hsa-miR-7-1-3p (miRBase21_mature_hsa) | -1.090 | 0.877 | -0.37396237 | -0.49885327 |
| hsa-miR-7151-5p (miRBase21_mature_hsa) | -1.090 | 0.877 | -0.006716645 | -0.13160752 |
| hsa-miR-7641 (miRBase21_mature_hsa) | -1.090 | 0.877 | -0.11401463 | -0.2389055 |
| hsa-miR-7849-3p (miRBase21_mature_hsa) | -1.090 | 0.877 | -0.093916506 | -0.21880737 |
| hsa-miR-7851-3p (miRBase21_mature_hsa) | -1.090 | 0.877 | -0.007949794 | -0.13284068 |
| hsa-miR-7855-5p (miRBase21_mature_hsa) | -1.090 | 0.877 | 0.001749134 | -0.12314176 |
| hsa-miR-7856-5p (miRBase21_mature_hsa) | -1.090 | 0.877 | -0.03922514 | -0.164116 |
| hsa-miR-7976 (miRBase21_mature_hsa) | -1.090 | 0.877 | -0.17299631 | -0.29788718 |
| hsa-miR-7978 (miRBase21_mature_hsa) | -1.090 | 0.877 | -0.021657933 | -0.14654882 |
| hsa-miR-576-5p (miRBase21_mature_hsa) | 1.148 | 0.882 | 0.09758296 | 0.29714504 |
| hsa-miR-3115 (miRBase21_mature_hsa) | -1.177 | 0.885 | 0.60897917 | 0.3738956 |
| hsa-miR-432-5p (miRBase21_mature_hsa) | 1.117 | 0.888 | -0.19850452 | -0.03849449 |
| hsa-miR-885-3p (miRBase21_mature_hsa) | -1.119 | 0.889 | 0.027024776 | -0.13556634 |
| hsa-miR-942-5p (miRBase21_mature_hsa) | -1.202 | 0.890 | 0.026893497 | -0.23892786 |
| hsa-miR-99b-5p (miRBase21_mature_hsa) | 1.083 | 0.891 | -0.07472969 | 0.040837664 |
| hsa-miR-21-5p (miRBase21_mature_hsa) | -1.107 | 0.891 | 0.21605057 | 0.0690254 |

| | | | | |
|---|---|---|---|---|
| hsa-miR-766-3p (miRBase21_mature_hsa) | 1.165 | 0.894 | -0.73599106 | -0.5161488 |
| hsa-let-7c-5p (miRBase21_mature_hsa) | -1.094 | 0.896 | 0.1404243 | 0.011336769 |
| hsa-miR-3615 (miRBase21_mature_hsa) | 1.072 | 0.897 | 0.12305288 | 0.22336258 |
| hsa-miR-411-5p (miRBase21_mature_hsa) | 1.181 | 0.898 | 0.28413165 | 0.52364844 |
| hsa-miR-378a-3p (miRBase21_mature_hsa) | -1.068 | 0.902 | 0.04730879 | -0.047203213 |
| hsa-miR-181a-3p (miRBase21_mature_hsa) | 1.178 | 0.903 | -0.26224652 | -0.02596423 |
| hsa-miR-224-5p (miRBase21_mature_hsa) | 1.169 | 0.908 | 0.07182088 | 0.29709598 |
| hsa-miR-628-3p (miRBase21_mature_hsa) | -1.138 | 0.911 | 0.7413253 | 0.555277 |
| hsa-miR-362-5p (miRBase21_mature_hsa) | -1.155 | 0.911 | 0.41842443 | 0.21064371 |
| hsa-let-7b-3p (miRBase21_mature_hsa) | 1.147 | 0.912 | -0.1880875 | 0.009644821 |
| hsa-miR-223-3p (miRBase21_mature_hsa) | 1.067 | 0.915 | -0.40854526 | -0.3144258 |
| hsa-miR-19b-3p (miRBase21_mature_hsa) | -1.077 | 0.921 | 0.12738676 | 0.020498278 |
| hsa-miR-424-3p (miRBase21_mature_hsa) | -1.070 | 0.922 | -0.092401326 | -0.18982744 |
| hsa-miR-199a-5p (miRBase21_mature_hsa) | 1.058 | 0.923 | -0.060507666 | 0.020924535 |
| hsa-miR-324-5p (miRBase21_mature_hsa) | -1.094 | 0.923 | 0.5592883 | 0.42908812 |
| hsa-miR-215-5p (miRBase21_mature_hsa) | -1.113 | 0.923 | -0.22463167 | -0.3789635 |
| hsa-miR-3605-3p (miRBase21_mature_hsa) | 1.107 | 0.926 | -0.81979454 | -0.67338157 |
| hsa-miR-155-5p (miRBase21_mature_hsa) | -1.068 | 0.926 | 0.09035513 | -0.004939505 |
| hsa-miR-150-5p (miRBase21_mature_hsa) | 1.036 | 0.927 | 0.6159464 | 0.66758186 |
| hsa-miR-574-5p (miRBase21_mature_hsa) | -1.218 | 0.928 | 0.13134727 | -0.15301795 |
| hsa-miR-30d-5p (miRBase21_mature_hsa) | -1.046 | 0.930 | 0.030736566 | -0.034222357 |
| hsa-miR-92b-5p (miRBase21_mature_hsa) | 1.120 | 0.931 | -1.4902134 | -1.3261361 |
| hsa-miR-451a (miRBase21_mature_hsa) | -1.049 | 0.934 | 0.32903472 | 0.25967026 |
| hsa-miR-25-3p (miRBase21_mature_hsa) | -1.032 | 0.936 | -0.02820509 | -0.0732813 |
| hsa-miR-27a-3p (miRBase21_mature_hsa) | -1.036 | 0.936 | 0.1832702 | 0.13275526 |
| hsa-let-7e-5p (miRBase21_mature_hsa) | 1.059 | 0.941 | 0.021408016 | 0.10470382 |
| hsa-miR-548az-5p (miRBase21_mature_hsa) | 1.078 | 0.941 | 0.22212835 | 0.33020678 |
| hsa-miR-154-5p (miRBase21_mature_hsa) | -1.080 | 0.942 | 0.5563561 | 0.44542512 |
| hsa-miR-212-5p (miRBase21_mature_hsa) | -1.080 | 0.942 | 0.2630271 | 0.15209618 |
| hsa-miR-6866-5p (miRBase21_mature_hsa) | -1.080 | 0.942 | -0.6974816 | -0.80841255 |
| hsa-miR-1228-5p (miRBase21_mature_hsa) | 1.096 | 0.943 | -0.48640007 | -0.35423455 |
| hsa-miR-1296-5p (miRBase21_mature_hsa) | 1.058 | 0.944 | 0.3948298 | 0.47649744 |
| hsa-miR-6894-3p (miRBase21_mature_hsa) | -1.062 | 0.944 | 0.24012117 | 0.15394211 |
| hsa-miR-323a-5p (miRBase21_mature_hsa) | -1.082 | 0.950 | 0.025967875 | -0.08738512 |
| hsa-miR-127-5p (miRBase21_mature_hsa) | -1.061 | 0.951 | 0.5525533 | 0.46661183 |
| hsa-miR-1226-3p (miRBase21_mature_hsa) | -1.043 | 0.955 | -0.22834498 | -0.28953442 |
| hsa-miR-6789-3p (miRBase21_mature_hsa) | -1.043 | 0.955 | 0.41509983 | 0.35391036 |
| hsa-miR-30a-3p (miRBase21_mature_hsa) | -1.088 | 0.955 | 0.37927276 | 0.25789958 |
| hsa-miR-2110 (miRBase21_mature_hsa) | -1.036 | 0.957 | 0.07362084 | 0.02306566 |
| hsa-miR-769-5p (miRBase21_mature_hsa) | 1.087 | 0.957 | -0.5176777 | -0.39685282 |
| hsa-miR-101-3p (miRBase21_mature_hsa) | 1.032 | 0.957 | 0.18341903 | 0.2291643 |
| hsa-miR-1301-3p (miRBase21_mature_hsa) | -1.035 | 0.958 | 0.25628468 | 0.20656978 |
| hsa-miR-1246 (miRBase21_mature_hsa) | -1.067 | 0.958 | -0.457269 | -0.5507719 |
| hsa-miR-625-3p (miRBase21_mature_hsa) | 1.027 | 0.958 | -0.26455757 | -0.22674015 |
| hsa-miR-365b-5p (miRBase21_mature_hsa) | 1.060 | 0.961 | 0.3569128 | 0.440504 |
| hsa-miR-218-5p (miRBase21_mature_hsa) | -1.060 | 0.963 | 1.1662205 | 1.0822744 |
| hsa-miR-3158-3p (miRBase21_mature_hsa) | -1.060 | 0.963 | -0.76484394 | -0.8493021 |
| hsa-miR-885-5p (miRBase21_mature_hsa) | 1.051 | 0.965 | 0.767383 | 0.83919054 |
| hsa-miR-130a-3p (miRBase21_mature_hsa) | -1.062 | 0.965 | 0.8563927 | 0.7697432 |
| hsa-miR-1304-3p (miRBase21_mature_hsa) | -1.042 | 0.971 | -1.0320692 | -1.0915997 |
| hsa-miR-6809-5p (miRBase21_mature_hsa) | -1.042 | 0.971 | 0.22381248 | 0.16428186 |
| hsa-miR-3613-5p (miRBase21_mature_hsa) | 1.029 | 0.972 | 0.9152899 | 0.95656 |
| hsa-miR-140-5p (miRBase21_mature_hsa) | 1.041 | 0.972 | 0.21994594 | 0.27825698 |
| hsa-miR-93-3p (miRBase21_mature_hsa) | 1.019 | 0.977 | -0.23255832 | -0.20570467 |
| hsa-miR-664a-5p (miRBase21_mature_hsa) | 1.035 | 0.977 | 0.1565216 | 0.2058938 |
| hsa-miR-7704 (miRBase21_mature_hsa) | -1.025 | 0.981 | -0.0351879 | -0.07027135 |
| hsa-miR-483-5p (miRBase21_mature_hsa) | -1.016 | 0.981 | 0.052064452 | 0.028605845 |
| hsa-miR-206 (miRBase21_mature_hsa) | -1.026 | 0.983 | -1.0332868 | -1.0709596 |
| hsa-miR-195-3p (miRBase21_mature_hsa) | 1.025 | 0.986 | -0.36721826 | -0.33102486 |
| hsa-miR-4446-3p (miRBase21_mature_hsa) | 1.008 | 0.992 | -0.052039206 | -0.041026182 |
| hsa-miR-1304-5p (miRBase21_mature_hsa) | -1.007 | 0.993 | -0.48184454 | -0.49217612 |
| hsa-miR-214-5p (miRBase21_mature_hsa) | -1.007 | 0.993 | -0.20510669 | -0.21543823 |
| hsa-miR-532-3p (miRBase21_mature_hsa) | -1.007 | 0.993 | 0.29009196 | 0.27976042 |
| hsa-miR-193a-5p (miRBase21_mature_hsa) | 1.010 | 0.993 | -0.2533798 | -0.23959973 |
| hsa-miR-1290 (miRBase21_mature_hsa) | 1.011 | 0.993 | -0.34105098 | -0.3259043 |
| hsa-let-7d-3p (miRBase21_mature_hsa) | 1.002 | 0.997 | -0.09339006 | -0.09067483 |
| hsa-miR-429 (miRBase21_mature_hsa) | 1.001 | 0.999 | 0.3775542 | 0.379656 |
| hsa-miR-4435 (miRBase21_mature_hsa) | 1.001 | 0.999 | -0.46073622 | -0.45863444 |
| hsa-miR-331-5p (miRBase21_mature_hsa) | -1.000 | 1.000 | 0.91789013 | 0.91756994 |

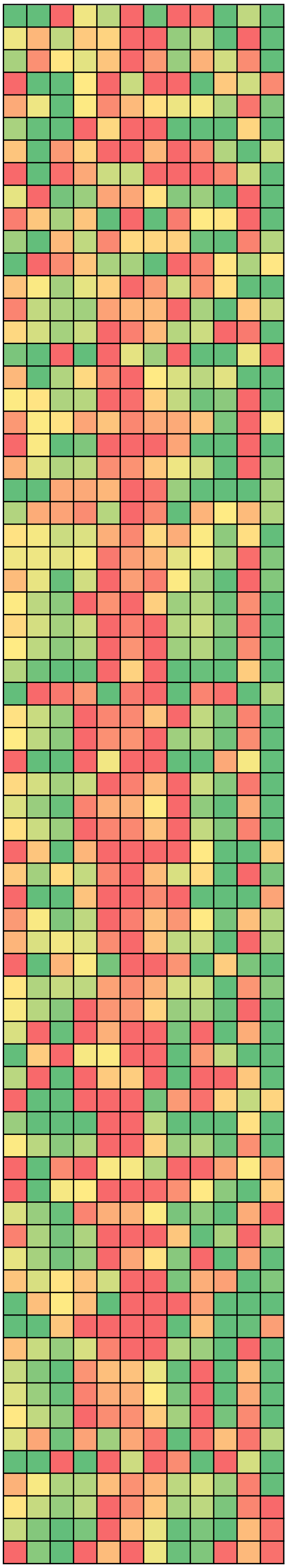

**Data Set for Table 3.**

| | | | Control | | | | | Persistent | | | | | | | Control | Persistent |
|---|---|---|---|---|---|---|---|---|---|---|---|---|---|---|---|---|
| | FV: Persitent vs Control | | FV #1 PS | FV #2 PS | FV #2 PS | FV #4 PS | FV #5 PS | FV #1 PS | FV #2 PS | FV #3 PS | FV #4 PS | FV #5 PS | FV #6 PS | FV #7 PS | FV #1-#5 Average | FV #1-#7 Average |
| ID | fold change | p-value | | | | | | | | | | | | | | |
| hsa-miR-1299 (miRBase21_mature_hsa) | -17.837 | 0.001 | | | | | | | | | | | | | 1.7785083 | -2.3783264 |
| hsa-miR-664b-5p (miRBase21_mature_hsa) | -14.650 | 0.003 | | | | | | | | | | | | | 2.9348772 | -0.9379074 |
| hsa-miR-20b-5p (miRBase21_mature_hsa) | -12.323 | 0.015 | | | | | | | | | | | | | 2.573876 | -1.0494258 |
| hsa-miR-589-5p (miRBase21_mature_hsa) | -16.461 | 0.015 | | | | | | | | | | | | | 2.7333224 | -1.3076657 |
| hsa-miR-4665-5p (miRBase21_mature_hsa) | -9.877 | 0.019 | | | | | | | | | | | | | 1.8729851 | -1.4310473 |
| hsa-miR-374a-5p (miRBase21_mature_hsa) | -20.900 | 0.025 | | | | | | | | | | | | | 1.6639929 | -2.721438 |
| hsa-miR-330-3p (miRBase21_mature_hsa) | -7.661 | 0.028 | | | | | | | | | | | | | 1.4837657 | -1.4537457 |
| hsa-miR-200c-3p (miRBase21_mature_hsa) | -24.759 | 0.030 | | | | | | | | | | | | | 1.1480632 | -3.4818463 |
| hsa-miR-204-5p (miRBase21_mature_hsa) | -11.020 | 0.031 | | | | | | | | | | | | | 2.294148 | -1.1679634 |
| hsa-miR-331-3p (miRBase21_mature_hsa) | -16.777 | 0.033 | | | | | | | | | | | | | 2.5399728 | -1.5284655 |
| hsa-miR-548j-5p (miRBase21_mature_hsa) | -13.170 | 0.034 | | | | | | | | | | | | | 2.3533776 | -1.3658426 |
| hsa-miR-431-5p (miRBase21_mature_hsa) | -18.582 | 0.034 | | | | | | | | | | | | | 2.9344094 | -1.2814221 |
| hsa-miR-151a-5p (miRBase21_mature_hsa) | -6.104 | 0.038 | | | | | | | | | | | | | 2.4128437 | -0.19684131 |
| hsa-miR-652-3p (miRBase21_mature_hsa) | -9.540 | 0.041 | | | | | | | | | | | | | 2.3451388 | -0.9088715 |
| hsa-miR-3120-3p (miRBase21_mature_hsa) | -10.712 | 0.047 | | | | | | | | | | | | | 2.42403 | -0.99719334 |
| hsa-miR-654-3p (miRBase21_mature_hsa) | -10.832 | 0.049 | | | | | | | | | | | | | 1.4805164 | -1.9566454 |
| hsa-miR-339-3p (miRBase21_mature_hsa) | -13.263 | 0.050 | | | | | | | | | | | | | 0.7835297 | -2.9458165 |
| hsa-miR-374b-5p (miRBase21_mature_hsa) | -10.630 | 0.054 | | | | | | | | | | | | | 0.81123316 | -2.5988712 |
| hsa-miR-576-3p (miRBase21_mature_hsa) | -5.149 | 0.057 | | | | | | | | | | | | | 1.7367287 | -0.62752515 |
| hsa-miR-5010-3p (miRBase21_mature_hsa) | -13.377 | 0.060 | | | | | | | | | | | | | 2.0334573 | -1.7082295 |
| hsa-miR-342-3p (miRBase21_mature_hsa) | -6.400 | 0.061 | | | | | | | | | | | | | 0.5439384 | -2.1342425 |
| hsa-let-7a-3p (miRBase21_mature_hsa) | -7.358 | 0.065 | | | | | | | | | | | | | 1.5415467 | -1.3377068 |
| hsa-miR-26b-3p (miRBase21_mature_hsa) | -9.613 | 0.066 | | | | | | | | | | | | | 2.7616732 | -0.50328743 |
| hsa-miR-10b-5p (miRBase21_mature_hsa) | 2.639 | 0.068 | | | | | | | | | | | | | -0.7737588 | 0.6263126 |
| hsa-miR-140-3p (miRBase21_mature_hsa) | -7.023 | 0.068 | | | | | | | | | | | | | 1.2315252 | -1.5806379 |
| hsa-miR-500a-3p (miRBase21_mature_hsa) | -6.684 | 0.069 | | | | | | | | | | | | | 2.3584363 | -0.38220683 |
| hsa-miR-766-5p (miRBase21_mature_hsa) | -4.597 | 0.072 | | | | | | | | | | | | | 1.5070708 | -0.69354135 |
| hsa-miR-145-5p (miRBase21_mature_hsa) | -6.673 | 0.073 | | | | | | | | | | | | | 1.564942 | -1.1734335 |
| hsa-miR-345-5p (miRBase21_mature_hsa) | -5.761 | 0.073 | | | | | | | | | | | | | 1.7503133 | -0.7758988 |
| hsa-miR-183-5p (miRBase21_mature_hsa) | 3.015 | 0.075 | | | | | | | | | | | | | -0.58115536 | 1.0110428 |
| hsa-miR-19b-3p (miRBase21_mature_hsa) | -8.375 | 0.075 | | | | | | | | | | | | | 2.0713534 | -0.99473137 |
| hsa-miR-24-2-5p (miRBase21_mature_hsa) | -7.103 | 0.081 | | | | | | | | | | | | | 2.3894267 | -0.43899372 |
| hsa-miR-4755-5p (miRBase21_mature_hsa) | -5.809 | 0.081 | | | | | | | | | | | | | 1.7456398 | -0.79273576 |
| hsa-miR-6772-3p (miRBase21_mature_hsa) | -9.066 | 0.082 | | | | | | | | | | | | | 2.7229939 | -0.45753688 |
| hsa-miR-411-5p (miRBase21_mature_hsa) | -11.059 | 0.083 | | | | | | | | | | | | | 1.2774637 | -2.189683 |
| hsa-miR-122-5p (miRBase21_mature_hsa) | 5.829 | 0.085 | | | | | | | | | | | | | -1.3032542 | 1.2399077 |
| hsa-miR-152-3p (miRBase21_mature_hsa) | -10.215 | 0.088 | | | | | | | | | | | | | 1.7046506 | -1.6479857 |
| hsa-miR-191-3p (miRBase21_mature_hsa) | -6.630 | 0.089 | | | | | | | | | | | | | 1.9858222 | -0.7431297 |
| hsa-miR-760 (miRBase21_mature_hsa) | -9.547 | 0.090 | | | | | | | | | | | | | 1.8096759 | -1.4453149 |
| hsa-miR-301a-5p (miRBase21_mature_hsa) | -4.663 | 0.092 | | | | | | | | | | | | | 1.9752942 | -0.24592152 |
| hsa-miR-374b-3p (miRBase21_mature_hsa) | -4.663 | 0.092 | | | | | | | | | | | | | 2.215428 | -0.005787532 |
| hsa-miR-4449 (miRBase21_mature_hsa) | -4.059 | 0.096 | | | | | | | | | | | | | 1.9057491 | -0.1154666 |
| hsa-miR-423-3p (miRBase21_mature_hsa) | -1.812 | 0.097 | | | | | | | | | | | | | 0.7175871 | -0.13988203 |
| hsa-miR-885-3p (miRBase21_mature_hsa) | 3.879 | 0.103 | | | | | | | | | | | | | -0.5022304 | 1.4536356 |
| hsa-miR-1538 (miRBase21_mature_hsa) | -3.528 | 0.105 | | | | | | | | | | | | | 1.3247993 | -0.4941485 |
| hsa-miR-193b-5p (miRBase21_mature_hsa) | 8.134 | 0.112 | | | | | | | | | | | | | -1.8629054 | 1.1611311 |
| hsa-miR-3074-5p (miRBase21_mature_hsa) | -5.012 | 0.114 | | | | | | | | | | | | | 2.4181838 | 0.09280013 |
| hsa-miR-548u (miRBase21_mature_hsa) | -4.053 | 0.117 | | | | | | | | | | | | | 1.2386299 | -0.78031796 |
| hsa-miR-375 (miRBase21_mature_hsa) | 6.379 | 0.117 | | | | | | | | | | | | | -1.9217789 | 0.75160754 |
| hsa-miR-200a-3p (miRBase21_mature_hsa) | 2.868 | 0.119 | | | | | | | | | | | | | -1.3429141 | 0.17703524 |
| hsa-miR-30d-3p (miRBase21_mature_hsa) | -8.162 | 0.119 | | | | | | | | | | | | | 2.182163 | -0.8467341 |
| hsa-miR-93-5p (miRBase21_mature_hsa) | -5.020 | 0.120 | | | | | | | | | | | | | 1.5265626 | -0.801081 |
| hsa-miR-370-3p (miRBase21_mature_hsa) | -2.932 | 0.121 | | | | | | | | | | | | | 1.5422411 | -0.009850128 |
| hsa-miR-1273h-3p (miRBase21_mature_hsa) | -4.484 | 0.121 | | | | | | | | | | | | | 1.7144761 | -0.45035547 |
| hsa-miR-3613-5p (miRBase21_mature_hsa) | -6.186 | 0.122 | | | | | | | | | | | | | 1.0227014 | -1.6061957 |
| hsa-miR-6741-3p (miRBase21_mature_hsa) | -4.477 | 0.122 | | | | | | | | | | | | | 2.9454281 | 0.7828514 |
| hsa-miR-1292-5p (miRBase21_mature_hsa) | -7.102 | 0.125 | | | | | | | | | | | | | 2.142367 | -0.68584377 |
| hsa-miR-23b-3p (miRBase21_mature_hsa) | -9.941 | 0.128 | | | | | | | | | | | | | 2.889365 | -0.4240509 |
| hsa-miR-323a-3p (miRBase21_mature_hsa) | -6.040 | 0.129 | | | | | | | | | | | | | 1.7010113 | -0.893434 |
| hsa-miR-365a-3p (miRBase21_mature_hsa) | -4.274 | 0.132 | | | | | | | | | | | | | 2.1938233 | 0.098149374 |
| hsa-miR-6855-3p (miRBase21_mature_hsa) | -4.274 | 0.132 | | | | | | | | | | | | | 1.8996221 | -0.1960519 |
| hsa-miR-1248 (miRBase21_mature_hsa) | -5.325 | 0.133 | | | | | | | | | | | | | 2.094809 | -0.3178573 |
| hsa-miR-23a-3p (miRBase21_mature_hsa) | -5.106 | 0.133 | | | | | | | | | | | | | 1.8140935 | -0.53816456 |
| hsa-miR-6516-5p (miRBase21_mature_hsa) | -4.925 | 0.135 | | | | | | | | | | | | | 2.7563877 | 0.45620728 |
| hsa-miR-25-5p (miRBase21_mature_hsa) | -5.385 | 0.136 | | | | | | | | | | | | | 0.90582883 | -1.5230683 |
| hsa-miR-181a-5p (miRBase21_mature_hsa) | -3.843 | 0.139 | | | | | | | | | | | | | 1.1948688 | -0.7474746 |

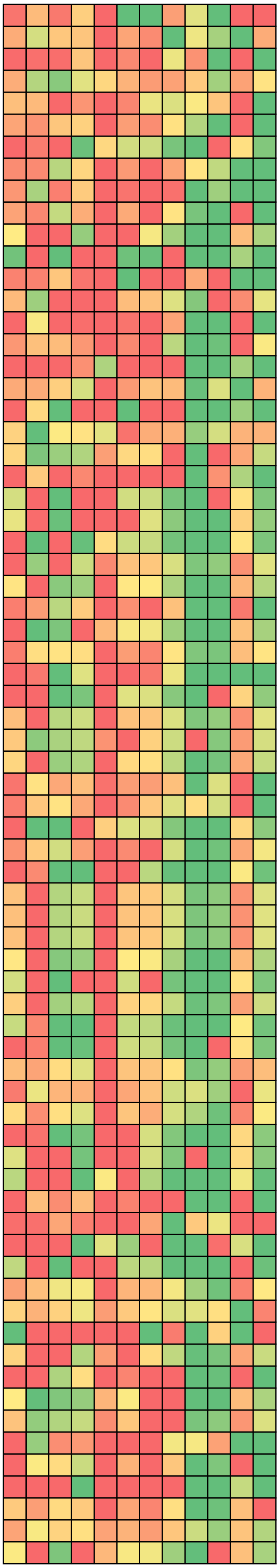

| | | | | |
|---|---|---|---|---|
| hsa-miR-22-5p (miRBase21_mature_hsa) | -5.313 | 0.149 | 1.5384678 | -0.8711605 |
| hsa-miR-222-3p (miRBase21_mature_hsa) | -3.305 | 0.150 | 0.84228444 | -0.8822143 |
| hsa-miR-181b-5p (miRBase21_mature_hsa) | -4.956 | 0.152 | 2.035655 | -0.2733767 |
| hsa-miR-100-5p (miRBase21_mature_hsa) | 1.815 | 0.154 | -0.31223547 | 0.5479476 |
| hsa-miR-363-3p (miRBase21_mature_hsa) | -4.187 | 0.155 | 1.4558839 | -0.60995245 |
| hsa-miR-199a-3p (miRBase21_mature_hsa) | -4.824 | 0.156 | 1.5300586 | -0.7400891 |
| hsa-miR-15b-3p (miRBase21_mature_hsa) | -4.435 | 0.160 | 1.6321158 | -0.51695 |
| hsa-miR-181a-2-3p (miRBase21_mature_hsa) | -4.052 | 0.162 | 1.100959 | -0.9175586 |
| hsa-miR-574-3p (miRBase21_mature_hsa) | -5.928 | 0.162 | 1.1854832 | -1.3820232 |
| hsa-miR-381-3p (miRBase21_mature_hsa) | -5.039 | 0.163 | 1.4272542 | -0.90588063 |
| hsa-miR-4435 (miRBase21_mature_hsa) | -4.399 | 0.165 | 1.8775911 | -0.2595744 |
| hsa-miR-19a-3p (miRBase21_mature_hsa) | -5.480 | 0.169 | 0.5853634 | -1.8688136 |
| hsa-miR-485-3p (miRBase21_mature_hsa) | -3.948 | 0.173 | 1.7881281 | -0.19302368 |
| hsa-miR-6511b-3p (miRBase21_mature_hsa) | -4.832 | 0.174 | 2.900345 | 0.6276063 |
| hsa-miR-3120-5p (miRBase21_mature_hsa) | -5.541 | 0.175 | 2.3336356 | -0.13658667 |
| hsa-miR-493-3p (miRBase21_mature_hsa) | -2.904 | 0.179 | 1.5395502 | 0.001716345 |
| hsa-miR-425-3p (miRBase21_mature_hsa) | -4.410 | 0.181 | 1.686115 | -0.45453027 |
| hsa-miR-340-5p (miRBase21_mature_hsa) | -2.815 | 0.186 | 0.8402881 | -0.6526553 |
| hsa-miR-3150a-5p (miRBase21_mature_hsa) | -6.923 | 0.186 | 1.6332824 | -1.1580588 |
| hsa-miR-486-3p (miRBase21_mature_hsa) | 2.155 | 0.188 | -0.5949272 | 0.5124308 |
| hsa-miR-1278 (miRBase21_mature_hsa) | 2.553 | 0.189 | -0.4825684 | 0.8698198 |
| hsa-miR-15b-5p (miRBase21_mature_hsa) | -3.333 | 0.195 | 2.064442 | 0.32755142 |
| hsa-miR-18a-5p (miRBase21_mature_hsa) | -4.150 | 0.197 | 1.5256407 | -0.52751464 |
| hsa-miR-210-3p (miRBase21_mature_hsa) | -3.824 | 0.198 | 1.3787408 | -0.5561569 |
| hsa-miR-877-3p (miRBase21_mature_hsa) | -3.962 | 0.203 | 0.5160611 | -1.4701345 |
| hsa-miR-4723-5p (miRBase21_mature_hsa) | -3.962 | 0.203 | 1.7965035 | -0.1896922 |
| hsa-miR-487b-3p (miRBase21_mature_hsa) | -4.645 | 0.210 | 1.4575237 | -0.75830305 |
| hsa-miR-382-5p (miRBase21_mature_hsa) | -3.985 | 0.213 | 1.0431708 | -0.95159 |
| hsa-miR-3605-5p (miRBase21_mature_hsa) | -4.290 | 0.216 | 1.175302 | -0.9256182 |
| hsa-miR-543 (miRBase21_mature_hsa) | -1.950 | 0.217 | 1.0228812 | 0.059171382 |
| hsa-miR-185-3p (miRBase21_mature_hsa) | -7.687 | 0.218 | 0.7915343 | -2.1508899 |
| hsa-miR-548ah-3p (miRBase21_mature_hsa) | -5.291 | 0.219 | 1.9345555 | -0.46897104 |
| hsa-miR-130b-3p (miRBase21_mature_hsa) | -3.521 | 0.221 | 1.6511979 | -0.16462879 |
| hsa-miR-27a-5p (miRBase21_mature_hsa) | 2.605 | 0.221 | -0.2939048 | 1.0871593 |
| hsa-miR-5584-5p (miRBase21_mature_hsa) | -4.386 | 0.226 | 1.5861213 | -0.54669803 |
| hsa-miR-16-5p (miRBase21_mature_hsa) | -3.045 | 0.228 | 1.1126639 | -0.49399596 |
| hsa-miR-340-3p (miRBase21_mature_hsa) | -3.390 | 0.228 | 1.2008961 | -0.5602992 |
| hsa-miR-455-5p (miRBase21_mature_hsa) | -3.444 | 0.229 | 0.52091277 | -1.263015 |
| hsa-miR-127-3p (miRBase21_mature_hsa) | -2.041 | 0.230 | 1.0348382 | 0.00562419 |
| hsa-miR-6515-5p (miRBase21_mature_hsa) | -5.071 | 0.236 | 1.320057 | -1.0223548 |
| hsa-miR-378d (miRBase21_mature_hsa) | -3.065 | 0.242 | 1.3823812 | -0.23344555 |
| hsa-miR-412-5p (miRBase21_mature_hsa) | -3.065 | 0.242 | 1.4181304 | -0.19769645 |
| hsa-miR-6813-5p (miRBase21_mature_hsa) | -3.065 | 0.242 | 1.3960435 | -0.21978322 |
| hsa-miR-331-5p (miRBase21_mature_hsa) | -3.065 | 0.242 | 0.80757254 | -0.80825424 |
| hsa-miR-758-3p (miRBase21_mature_hsa) | -4.177 | 0.246 | 1.503228 | -0.5592415 |
| hsa-miR-4661-5p (miRBase21_mature_hsa) | -3.521 | 0.247 | 1.3870643 | -0.4287625 |
| hsa-miR-125a-3p (miRBase21_mature_hsa) | -3.521 | 0.247 | 0.23761004 | -1.5782168 |
| hsa-miR-1304-3p (miRBase21_mature_hsa) | -4.606 | 0.250 | 1.5115047 | -0.6920221 |
| hsa-miR-26a-5p (miRBase21_mature_hsa) | -1.699 | 0.250 | 0.7982368 | 0.033544708 |
| hsa-miR-155-5p (miRBase21_mature_hsa) | -1.796 | 0.253 | 1.1426926 | 0.29771924 |
| hsa-miR-199a-5p (miRBase21_mature_hsa) | -1.830 | 0.254 | 0.826999 | -0.044962782 |
| hsa-miR-146b-3p (miRBase21_mature_hsa) | -4.071 | 0.256 | 1.35799 | -0.6674293 |
| hsa-miR-454-5p (miRBase21_mature_hsa) | -3.933 | 0.261 | 2.117896 | 0.14224003 |
| hsa-miR-107 (miRBase21_mature_hsa) | -3.755 | 0.265 | 0.90456796 | -1.0042977 |
| hsa-miR-3177-3p (miRBase21_mature_hsa) | -4.290 | 0.271 | 2.1132262 | 0.012284364 |
| hsa-miR-142-3p (miRBase21_mature_hsa) | -2.753 | 0.272 | 1.9021049 | 0.44105333 |
| hsa-miR-133a-3p (miRBase21_mature_hsa) | -3.122 | 0.272 | 0.96167326 | -0.68076694 |
| hsa-miR-361-5p (miRBase21_mature_hsa) | -4.016 | 0.276 | 1.2659407 | -0.73972696 |
| hsa-miR-126-3p (miRBase21_mature_hsa) | -1.670 | 0.280 | 0.7708877 | 0.03105402 |
| hsa-miR-22-3p (miRBase21_mature_hsa) | -2.717 | 0.280 | 0.53197235 | -0.91015905 |
| hsa-miR-181d-5p (miRBase21_mature_hsa) | -4.006 | 0.283 | 1.976293 | -0.025784357 |
| hsa-miR-605-3p (miRBase21_mature_hsa) | -2.672 | 0.285 | 1.5258845 | 0.10814659 |
| hsa-miR-379-5p (miRBase21_mature_hsa) | -3.644 | 0.290 | 1.5274373 | -0.33824468 |
| hsa-miR-125b-2-3p (miRBase21_mature_hsa) | 2.394 | 0.293 | -0.81494427 | 0.4445638 |
| hsa-miR-3690 (miRBase21_mature_hsa) | 2.394 | 0.293 | -0.20711909 | 1.052389 |
| hsa-miR-6747-3p (miRBase21_mature_hsa) | -2.835 | 0.294 | 1.4620016 | -0.041285396 |
| hsa-miR-671-3p (miRBase21_mature_hsa) | -3.391 | 0.296 | 1.0563236 | -0.70540845 |
| hsa-miR-4746-5p (miRBase21_mature_hsa) | -4.322 | 0.296 | 2.2189379 | 0.107278146 |
| hsa-miR-199b-3p (miRBase21_mature_hsa) | -2.084 | 0.297 | 1.3224831 | 0.2629449 |
| hsa-miR-27a-3p (miRBase21_mature_hsa) | -1.408 | 0.298 | 0.539206 | 0.045782346 |
| hsa-let-7f-1-3p (miRBase21_mature_hsa) | -2.423 | 0.302 | 1.105445 | -0.17113988 |

| | | | | |
|---|---|---|---|---|
| hsa-miR-548z (miRBase21_mature_hsa) | -2.138 | 0.304 | 0.68355495 | -0.41284418 |
| hsa-miR-1296-5p (miRBase21_mature_hsa) | -2.138 | 0.304 | 1.1136649 | 0.017265763 |
| hsa-miR-335-5p (miRBase21_mature_hsa) | -3.895 | 0.304 | 2.035668 | 0.073988155 |
| hsa-miR-130a-3p (miRBase21_mature_hsa) | -3.274 | 0.306 | 0.79445 | -0.91682047 |
| hsa-miR-1180-3p (miRBase21_mature_hsa) | 2.698 | 0.307 | -0.71550643 | 0.7164844 |
| hsa-miR-584-5p (miRBase21_mature_hsa) | -3.647 | 0.308 | 0.81145686 | -1.0552652 |
| hsa-let-7b-5p (miRBase21_mature_hsa) | 1.827 | 0.309 | -0.3601821 | 0.5092715 |
| hsa-miR-382-3p (miRBase21_mature_hsa) | -3.947 | 0.313 | 0.68144816 | -1.2993586 |
| hsa-miR-191-5p (miRBase21_mature_hsa) | -1.585 | 0.315 | 0.77108353 | 0.10621789 |
| hsa-miR-181c-3p (miRBase21_mature_hsa) | -1.861 | 0.316 | 0.08685382 | -0.80954534 |
| hsa-miR-3161 (miRBase21_mature_hsa) | -1.861 | 0.316 | 0.805824 | -0.09057514 |
| hsa-miR-1185-1-3p (miRBase21_mature_hsa) | -1.861 | 0.316 | 0.43594655 | -0.4604526 |
| hsa-miR-410-3p (miRBase21_mature_hsa) | -1.861 | 0.316 | 0.8372618 | -0.05913737 |
| hsa-miR-874-3p (miRBase21_mature_hsa) | -1.861 | 0.316 | 0.4240444 | -0.47235477 |
| hsa-miR-377-3p (miRBase21_mature_hsa) | -1.861 | 0.316 | 0.9657744 | 0.069375254 |
| hsa-miR-4762-3p (miRBase21_mature_hsa) | -1.861 | 0.316 | 0.86591226 | -0.030486884 |
| hsa-miR-6876-3p (miRBase21_mature_hsa) | -1.861 | 0.316 | 1.0152655 | 0.11886629 |
| hsa-miR-1249-3p (miRBase21_mature_hsa) | -1.861 | 0.316 | 0.38895267 | -0.50744647 |
| hsa-miR-6819-3p (miRBase21_mature_hsa) | -1.861 | 0.316 | 0.93064797 | 0.034248795 |
| hsa-miR-6777-3p (miRBase21_mature_hsa) | -1.861 | 0.316 | 0.6772944 | -0.2191048 |
| hsa-miR-1291 (miRBase21_mature_hsa) | -1.861 | 0.316 | 0.7721808 | -0.12421838 |
| hsa-miR-3127-3p (miRBase21_mature_hsa) | -1.861 | 0.316 | 0.1630996 | -0.73329955 |
| hsa-miR-4742-3p (miRBase21_mature_hsa) | -1.861 | 0.316 | 0.43223605 | -0.4641631 |
| hsa-let-7f-2-3p (miRBase21_mature_hsa) | -1.861 | 0.316 | 1.0507127 | 0.15431352 |
| hsa-miR-15a-5p (miRBase21_mature_hsa) | -1.861 | 0.316 | 1.1249045 | 0.22850533 |
| hsa-miR-17-3p (miRBase21_mature_hsa) | -1.861 | 0.316 | 1.1249045 | 0.22850533 |
| hsa-miR-181c-5p (miRBase21_mature_hsa) | -1.861 | 0.316 | 1.1249045 | 0.22850533 |
| hsa-miR-3184-5p (miRBase21_mature_hsa) | -1.861 | 0.316 | 1.1249045 | 0.22850533 |
| hsa-miR-320e (miRBase21_mature_hsa) | -1.861 | 0.316 | 1.1249045 | 0.22850533 |
| hsa-miR-3687 (miRBase21_mature_hsa) | -1.861 | 0.316 | 1.1249045 | 0.22850533 |
| hsa-miR-449c-5p (miRBase21_mature_hsa) | -1.861 | 0.316 | 1.1249045 | 0.22850533 |
| hsa-miR-4738-3p (miRBase21_mature_hsa) | -1.861 | 0.316 | 1.037495 | 0.14109594 |
| hsa-miR-500b-3p (miRBase21_mature_hsa) | -1.861 | 0.316 | 1.1249045 | 0.22850533 |
| hsa-miR-548t-3p (miRBase21_mature_hsa) | -1.861 | 0.316 | 1.1249045 | 0.22850533 |
| hsa-miR-605-5p (miRBase21_mature_hsa) | -1.861 | 0.316 | 1.1249045 | 0.22850533 |
| hsa-miR-616-3p (miRBase21_mature_hsa) | -1.861 | 0.316 | 0.95206285 | 0.05566368 |
| hsa-miR-6809-3p (miRBase21_mature_hsa) | -1.861 | 0.316 | 1.0117689 | 0.1153698 |
| hsa-miR-7111-3p (miRBase21_mature_hsa) | -1.861 | 0.316 | 0.9097771 | 0.013377984 |
| hsa-miR-320b (miRBase21_mature_hsa) | -2.924 | 0.316 | 0.42071977 | -1.1274269 |
| hsa-miR-136-5p (miRBase21_mature_hsa) | 2.696 | 0.317 | -1.0385118 | 0.39256343 |
| hsa-miR-4664-3p (miRBase21_mature_hsa) | -3.947 | 0.318 | 0.41049513 | -1.5703117 |
| hsa-miR-125b-1-3p (miRBase21_mature_hsa) | 2.545 | 0.325 | -1.6085212 | -0.2610121 |
| hsa-miR-1-3p (miRBase21_mature_hsa) | -4.307 | 0.326 | 1.7839562 | -0.32271916 |
| hsa-miR-200b-3p (miRBase21_mature_hsa) | -3.436 | 0.331 | -0.32801375 | -2.1088204 |
| hsa-miR-369-3p (miRBase21_mature_hsa) | -3.358 | 0.335 | 2.504922 | 0.75737077 |
| hsa-miR-425-5p (miRBase21_mature_hsa) | -1.489 | 0.335 | 0.8263727 | 0.2523606 |
| hsa-miR-2355-3p (miRBase21_mature_hsa) | 2.284 | 0.335 | 0.4143823 | 1.6056899 |
| hsa-miR-192-5p (miRBase21_mature_hsa) | -2.551 | 0.336 | -0.010948834 | -1.3622807 |
| hsa-miR-204-3p (miRBase21_mature_hsa) | -3.783 | 0.336 | 0.71639895 | -1.2030091 |
| hsa-miR-6786-3p (miRBase21_mature_hsa) | -2.590 | 0.340 | 0.70667726 | -0.666448 |
| hsa-miR-30c-1-3p (miRBase21_mature_hsa) | -3.851 | 0.341 | 1.8975046 | -0.04768812 |
| hsa-miR-636 (miRBase21_mature_hsa) | 2.805 | 0.342 | -0.055083096 | 1.4327939 |
| hsa-miR-9-5p (miRBase21_mature_hsa) | 2.305 | 0.344 | -0.5487114 | 0.6559405 |
| hsa-miR-4498 (miRBase21_mature_hsa) | 2.305 | 0.344 | -0.10555985 | 1.099092 |
| hsa-miR-628-5p (miRBase21_mature_hsa) | 2.305 | 0.344 | 0.035586126 | 1.2402381 |
| hsa-miR-203a-3p (miRBase21_mature_hsa) | -2.255 | 0.345 | 0.8712405 | -0.3018848 |
| hsa-miR-6734-5p (miRBase21_mature_hsa) | -2.255 | 0.345 | 0.3976528 | -0.77547246 |
| hsa-miR-4700-5p (miRBase21_mature_hsa) | -2.255 | 0.345 | 1.3056902 | 0.13256487 |
| hsa-miR-4685-3p (miRBase21_mature_hsa) | -2.255 | 0.345 | 0.5675122 | -0.60561305 |
| hsa-miR-6803-3p (miRBase21_mature_hsa) | -2.255 | 0.345 | 0.96288556 | -0.21023968 |
| hsa-miR-1273d (miRBase21_mature_hsa) | -2.255 | 0.345 | 1.2670542 | 0.09392893 |
| hsa-miR-4440 (miRBase21_mature_hsa) | -2.255 | 0.345 | 1.3715516 | 0.19842643 |
| hsa-miR-491-5p (miRBase21_mature_hsa) | -2.255 | 0.345 | 1.3715516 | 0.19842643 |
| hsa-miR-501-5p (miRBase21_mature_hsa) | -2.255 | 0.345 | 1.3715516 | 0.19842643 |
| hsa-miR-6754-3p (miRBase21_mature_hsa) | -2.255 | 0.345 | 1.3715516 | 0.19842643 |
| hsa-miR-6770-3p (miRBase21_mature_hsa) | -2.991 | 0.346 | 1.6146687 | 0.03386196 |
| hsa-miR-1468-5p (miRBase21_mature_hsa) | -2.545 | 0.349 | 0.41134122 | -0.93644804 |
| hsa-miR-494-3p (miRBase21_mature_hsa) | -3.394 | 0.352 | 1.6625203 | -0.100639164 |
| hsa-miR-495-3p (miRBase21_mature_hsa) | -2.366 | 0.355 | 1.2264861 | -0.015805628 |
| hsa-miR-145-3p (miRBase21_mature_hsa) | -2.045 | 0.362 | 1.0136256 | -0.018658461 |
| hsa-miR-548am-3p (miRBase21_mature_hsa) | -2.804 | 0.362 | 1.5849171 | 0.09723441 |

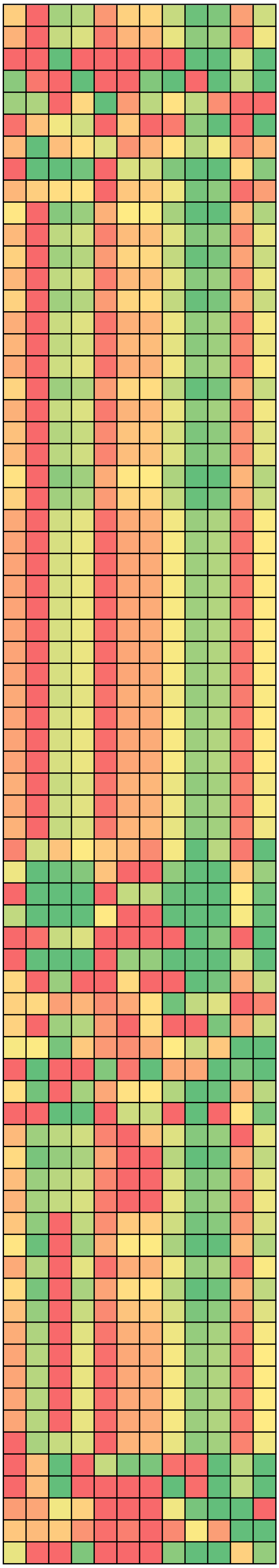

| | | | | |
|---|---|---|---|---|
| hsa-miR-224-5p (miRBase21_mature_hsa) | -4.682 | 0.366 | 0.29554847 | -1.9316226 |
| hsa-miR-146a-5p (miRBase21_mature_hsa) | -1.822 | 0.367 | 0.8675855 | 0.002391347 |
| hsa-miR-539-5p (miRBase21_mature_hsa) | -3.955 | 0.367 | 1.6312513 | -0.35224712 |
| hsa-miR-339-5p (miRBase21_mature_hsa) | -2.313 | 0.369 | 1.2346241 | 0.02496534 |
| hsa-miR-30a-3p (miRBase21_mature_hsa) | -3.556 | 0.369 | 0.89228934 | -0.93796855 |
| hsa-miR-6862-5p (miRBase21_mature_hsa) | -2.251 | 0.371 | 0.84075177 | -0.3301057 |
| hsa-miR-10b-3p (miRBase21_mature_hsa) | -2.251 | 0.371 | 1.3695303 | 0.19867288 |
| hsa-miR-29a-3p (miRBase21_mature_hsa) | -2.153 | 0.371 | 0.59048647 | -0.51584065 |
| hsa-let-7g-5p (miRBase21_mature_hsa) | -2.236 | 0.373 | 1.202692 | 0.042057797 |
| hsa-miR-125a-5p (miRBase21_mature_hsa) | 1.268 | 0.373 | -0.20491035 | 0.13805561 |
| hsa-miR-103a-3p (miRBase21_mature_hsa) | -2.068 | 0.374 | 1.1775957 | 0.12916298 |
| hsa-miR-5193 (miRBase21_mature_hsa) | -2.780 | 0.375 | 2.0236802 | 0.5488035 |
| hsa-miR-641 (miRBase21_mature_hsa) | -3.173 | 0.376 | 2.727872 | 1.0622157 |
| hsa-miR-4669 (miRBase21_mature_hsa) | -1.960 | 0.387 | 0.9993326 | 0.028475104 |
| hsa-miR-487a-3p (miRBase21_mature_hsa) | -1.960 | 0.387 | 1.1227176 | 0.15186009 |
| hsa-miR-33a-5p (miRBase21_mature_hsa) | -1.960 | 0.387 | 1.0921717 | 0.12131422 |
| hsa-miR-372-3p (miRBase21_mature_hsa) | -1.960 | 0.387 | 1.1912695 | 0.22041202 |
| hsa-miR-4723-3p (miRBase21_mature_hsa) | -1.960 | 0.387 | 1.0857359 | 0.114878476 |
| hsa-miR-490-5p (miRBase21_mature_hsa) | -1.960 | 0.387 | 1.1912695 | 0.22041202 |
| hsa-miR-509-3-5p (miRBase21_mature_hsa) | -1.960 | 0.387 | 0.97659075 | 0.005733224 |
| hsa-miR-656-3p (miRBase21_mature_hsa) | -1.960 | 0.387 | 1.1170777 | 0.1462202 |
| hsa-miR-6861-5p (miRBase21_mature_hsa) | -1.960 | 0.387 | 0.9998149 | 0.028957477 |
| hsa-miR-7851-3p (miRBase21_mature_hsa) | -1.960 | 0.387 | 1.1912695 | 0.22041202 |
| hsa-miR-98-5p (miRBase21_mature_hsa) | -1.899 | 0.387 | 1.2142161 | 0.28906408 |
| hsa-miR-451a (miRBase21_mature_hsa) | 1.539 | 0.388 | -0.1894469 | 0.43299606 |
| hsa-miR-6850-5p (miRBase21_mature_hsa) | 1.714 | 0.389 | 0.036637247 | 0.8142236 |
| hsa-miR-1303 (miRBase21_mature_hsa) | 1.714 | 0.389 | 0.077729926 | 0.8553163 |
| hsa-miR-196a-5p (miRBase21_mature_hsa) | 1.714 | 0.389 | 0.11650569 | 0.89409196 |
| hsa-miR-214-3p (miRBase21_mature_hsa) | 1.714 | 0.389 | 0.15192173 | 0.92950803 |
| hsa-miR-4732-3p (miRBase21_mature_hsa) | -2.680 | 0.399 | 0.49546 | -0.92687315 |
| hsa-miR-1284 (miRBase21_mature_hsa) | -2.510 | 0.401 | 1.3886652 | 0.06080352 |
| hsa-miR-766-3p (miRBase21_mature_hsa) | -2.597 | 0.403 | 0.55710703 | -0.81967366 |
| hsa-miR-6843-3p (miRBase21_mature_hsa) | -2.993 | 0.403 | 1.2779478 | -0.30364186 |
| hsa-miR-320a (miRBase21_mature_hsa) | -1.445 | 0.406 | 0.5045809 | -0.026924053 |
| hsa-miR-342-5p (miRBase21_mature_hsa) | 1.712 | 0.406 | 0.09935177 | 0.87473446 |
| hsa-miR-148a-5p (miRBase21_mature_hsa) | -2.962 | 0.407 | 0.65492386 | -0.91142005 |
| hsa-miR-744-5p (miRBase21_mature_hsa) | -1.609 | 0.413 | 0.6675927 | -0.018198898 |
| hsa-miR-1183 (miRBase21_mature_hsa) | -2.564 | 0.417 | 1.2373996 | -0.12115763 |
| hsa-miR-6511a-5p (miRBase21_mature_hsa) | 2.200 | 0.420 | 0.06867875 | 1.2064003 |
| hsa-miR-6808-3p (miRBase21_mature_hsa) | 2.200 | 0.420 | 0.06773187 | 1.2054534 |
| hsa-let-7d-5p (miRBase21_mature_hsa) | -1.779 | 0.420 | 1.1296915 | 0.29891753 |
| hsa-miR-1228-5p (miRBase21_mature_hsa) | 2.920 | 0.422 | -1.3353399 | 0.21068844 |
| hsa-miR-548o-3p (miRBase21_mature_hsa) | -4.062 | 0.424 | 1.0968194 | -0.9253159 |
| hsa-miR-21-5p (miRBase21_mature_hsa) | -1.592 | 0.429 | 0.8885088 | 0.21773277 |
| hsa-miR-6805-5p (miRBase21_mature_hsa) | 1.618 | 0.430 | 0.06539211 | 0.7594463 |
| hsa-miR-6821-5p (miRBase21_mature_hsa) | 1.618 | 0.430 | 0.04072068 | 0.73477495 |
| hsa-miR-1234-3p (miRBase21_mature_hsa) | 1.618 | 0.430 | 0.16862012 | 0.86267436 |
| hsa-miR-6516-3p (miRBase21_mature_hsa) | 1.618 | 0.430 | 0.16367278 | 0.85772705 |
| hsa-miR-1908-5p (miRBase21_mature_hsa) | -2.138 | 0.431 | 0.3826393 | -0.713439 |
| hsa-miR-23a-5p (miRBase21_mature_hsa) | 2.479 | 0.431 | -1.4217713 | -0.11205367 |
| hsa-miR-18a-3p (miRBase21_mature_hsa) | -2.783 | 0.432 | -0.22124843 | -1.697983 |
| hsa-miR-150-5p (miRBase21_mature_hsa) | 2.597 | 0.432 | -1.1496899 | 0.22710037 |
| hsa-miR-23b-5p (miRBase21_mature_hsa) | -3.350 | 0.433 | 0.38185838 | -1.362403 |
| hsa-miR-221-3p (miRBase21_mature_hsa) | -1.723 | 0.434 | 1.0969815 | 0.3124698 |
| hsa-miR-454-3p (miRBase21_mature_hsa) | -2.679 | 0.439 | 1.3700767 | -0.051801786 |
| hsa-miR-885-5p (miRBase21_mature_hsa) | 2.246 | 0.441 | -0.053613834 | 1.1137096 |
| hsa-miR-450a-5p (miRBase21_mature_hsa) | -2.535 | 0.447 | 1.3060246 | -0.035680342 |
| hsa-miR-369-5p (miRBase21_mature_hsa) | -2.474 | 0.449 | 1.9564259 | 0.64933264 |
| hsa-miR-411-3p (miRBase21_mature_hsa) | -2.646 | 0.451 | 0.50083226 | -0.90296763 |
| hsa-miR-378a-3p (miRBase21_mature_hsa) | -1.307 | 0.454 | 0.17637052 | -0.2094514 |
| hsa-miR-6855-5p (miRBase21_mature_hsa) | 2.208 | 0.458 | 0.20473757 | 1.3475332 |
| hsa-miR-30e-3p (miRBase21_mature_hsa) | -1.692 | 0.458 | 0.808172 | 0.049657714 |
| hsa-miR-7848-3p (miRBase21_mature_hsa) | -2.511 | 0.459 | 1.2181216 | -0.110360086 |
| hsa-miR-195-3p (miRBase21_mature_hsa) | 2.807 | 0.460 | -0.6905412 | 0.7982857 |
| hsa-miR-132-5p (miRBase21_mature_hsa) | -2.367 | 0.463 | 0.5672098 | -0.6758603 |
| hsa-miR-10a-5p (miRBase21_mature_hsa) | 1.447 | 0.465 | -0.27690437 | 0.2565366 |
| hsa-miR-20a-5p (miRBase21_mature_hsa) | -1.693 | 0.466 | 1.5381789 | 0.77877814 |
| hsa-miR-4647 (miRBase21_mature_hsa) | -2.823 | 0.466 | 1.2723207 | -0.2251442 |
| hsa-miR-493-5p (miRBase21_mature_hsa) | -2.458 | 0.467 | 1.921578 | 0.6240781 |
| hsa-miR-1307-3p (miRBase21_mature_hsa) | -1.417 | 0.472 | 0.5987765 | 0.09626965 |
| hsa-miR-193a-5p (miRBase21_mature_hsa) | -2.177 | 0.473 | -0.15406476 | -1.2761577 |

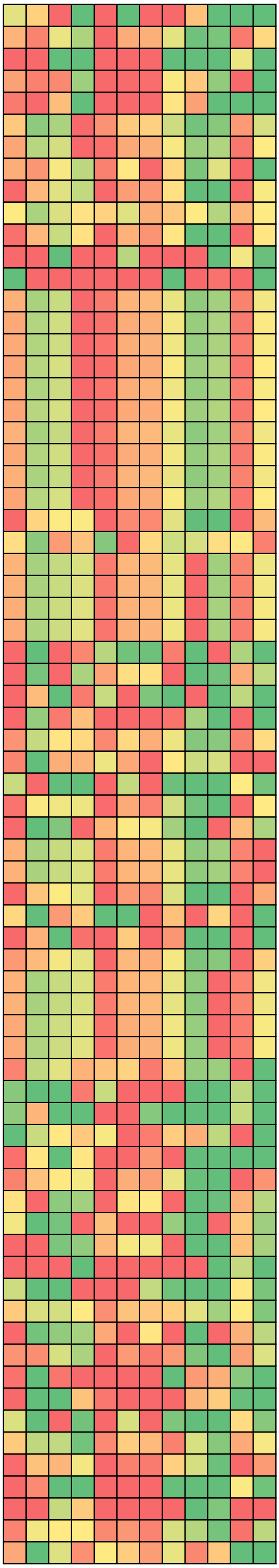

| | | | | |
|---|---|---|---|---|
| hsa-miR-3936 (miRBase21_mature_hsa) | 1.685 | 0.474 | 0.13017353 | 0.88305587 |
| hsa-miR-129-5p (miRBase21_mature_hsa) | 1.685 | 0.474 | -1.1877447 | -0.4348624 |
| hsa-miR-6809-5p (miRBase21_mature_hsa) | 1.685 | 0.474 | -0.1301947 | 0.62268764 |
| hsa-miR-106b-3p (miRBase21_mature_hsa) | -1.596 | 0.478 | 0.63579637 | -0.038875546 |
| hsa-miR-1301-3p (miRBase21_mature_hsa) | -1.477 | 0.484 | 0.765942 | 0.20367447 |
| hsa-miR-28-3p (miRBase21_mature_hsa) | -1.569 | 0.494 | 0.55866563 | -0.09114028 |
| hsa-miR-30b-3p (miRBase21_mature_hsa) | -2.495 | 0.496 | 1.1667964 | -0.15197308 |
| hsa-miR-4709-3p (miRBase21_mature_hsa) | -2.017 | 0.497 | 1.6742998 | 0.6619509 |
| hsa-miR-30d-5p (miRBase21_mature_hsa) | -1.336 | 0.498 | 0.52709794 | 0.10950102 |
| hsa-miR-185-5p (miRBase21_mature_hsa) | -1.571 | 0.498 | 0.6219112 | -0.029406684 |
| hsa-miR-4732-5p (miRBase21_mature_hsa) | 2.222 | 0.499 | -1.2171179 | -0.06530379 |
| hsa-miR-26b-5p (miRBase21_mature_hsa) | -1.490 | 0.501 | 0.85537374 | 0.2802526 |
| hsa-miR-148b-3p (miRBase21_mature_hsa) | -1.611 | 0.503 | 0.75651443 | 0.06817706 |
| hsa-miR-5698 (miRBase21_mature_hsa) | -1.817 | 0.507 | 0.8687542 | 0.007375119 |
| hsa-miR-6847-5p (miRBase21_mature_hsa) | -1.817 | 0.507 | 0.48897725 | -0.37240183 |
| hsa-miR-450b-5p (miRBase21_mature_hsa) | -1.817 | 0.507 | 0.17976932 | -0.68160975 |
| hsa-miR-6515-3p (miRBase21_mature_hsa) | -1.817 | 0.507 | 0.46092033 | -0.40045875 |
| hsa-miR-9-3p (miRBase21_mature_hsa) | -1.817 | 0.507 | 0.92333204 | 0.061952915 |
| hsa-miR-942-3p (miRBase21_mature_hsa) | -1.817 | 0.507 | 1.0278294 | 0.16645035 |
| hsa-miR-3675-5p (miRBase21_mature_hsa) | -1.817 | 0.507 | 0.8289832 | -0.032395907 |
| hsa-miR-4742-5p (miRBase21_mature_hsa) | -1.817 | 0.507 | 0.75321996 | -0.10815909 |
| hsa-miR-1914-5p (miRBase21_mature_hsa) | -1.817 | 0.507 | 0.46060887 | -0.40077022 |
| hsa-miR-6806-3p (miRBase21_mature_hsa) | -1.817 | 0.507 | 0.78635347 | -0.0750256 |
| hsa-miR-410-5p (miRBase21_mature_hsa) | -1.817 | 0.507 | 1.0936909 | 0.23231184 |
| hsa-miR-4717-3p (miRBase21_mature_hsa) | -1.817 | 0.507 | 1.0936909 | 0.23231184 |
| hsa-miR-542-3p (miRBase21_mature_hsa) | -1.817 | 0.507 | 0.99776006 | 0.13638091 |
| hsa-miR-548az-5p (miRBase21_mature_hsa) | -1.817 | 0.507 | 0.7059434 | -0.15543571 |
| hsa-miR-7704 (miRBase21_mature_hsa) | -1.817 | 0.507 | 0.42387524 | -0.4375038 |
| hsa-miR-181a-3p (miRBase21_mature_hsa) | -2.263 | 0.510 | 1.3890866 | 0.21086349 |
| hsa-miR-99a-5p (miRBase21_mature_hsa) | 1.275 | 0.515 | -0.094045684 | 0.25627303 |
| hsa-miR-671-5p (miRBase21_mature_hsa) | 1.526 | 0.517 | 0.070237294 | 0.6802625 |
| hsa-miR-6837-3p (miRBase21_mature_hsa) | 1.526 | 0.517 | -0.4848167 | 0.1252085 |
| hsa-miR-143-5p (miRBase21_mature_hsa) | 1.526 | 0.517 | -0.6528919 | -0.04286673 |
| hsa-miR-200b-5p (miRBase21_mature_hsa) | 1.526 | 0.517 | 0.076391086 | 0.68641627 |
| hsa-miR-365b-3p (miRBase21_mature_hsa) | 1.526 | 0.517 | 0.09813021 | 0.7081554 |
| hsa-miR-4676-3p (miRBase21_mature_hsa) | 1.526 | 0.517 | 0.20026463 | 0.81028986 |
| hsa-miR-147b (miRBase21_mature_hsa) | 1.526 | 0.517 | 0.14898036 | 0.7590056 |
| hsa-miR-5587-3p (miRBase21_mature_hsa) | 1.526 | 0.517 | -0.14027089 | 0.46975434 |
| hsa-miR-1273c (miRBase21_mature_hsa) | 1.526 | 0.517 | 0.06353281 | 0.673558 |
| hsa-miR-296-5p (miRBase21_mature_hsa) | 1.526 | 0.517 | -0.2134302 | 0.39659503 |
| hsa-miR-3199 (miRBase21_mature_hsa) | 1.526 | 0.517 | 0.16971873 | 0.7797439 |
| hsa-miR-32-3p (miRBase21_mature_hsa) | 1.526 | 0.517 | -0.34750894 | 0.26251623 |
| hsa-miR-4632-5p (miRBase21_mature_hsa) | 1.526 | 0.517 | 0.26881656 | 0.87884176 |
| hsa-miR-4765 (miRBase21_mature_hsa) | 1.526 | 0.517 | 0.18140715 | 0.7914324 |
| hsa-miR-581 (miRBase21_mature_hsa) | 1.526 | 0.517 | 0.07963795 | 0.6896632 |
| hsa-miR-6787-3p (miRBase21_mature_hsa) | -2.020 | 0.518 | 1.3981584 | 0.38352272 |
| hsa-miR-144-3p (miRBase21_mature_hsa) | -2.229 | 0.518 | 0.65601814 | -0.5003253 |
| hsa-miR-379-3p (miRBase21_mature_hsa) | -1.954 | 0.521 | 1.1933732 | 0.22702934 |
| hsa-miR-134-5p (miRBase21_mature_hsa) | -1.543 | 0.525 | 0.769671 | 0.14380145 |
| hsa-miR-511-5p (miRBase21_mature_hsa) | -2.255 | 0.526 | 1.1006159 | -0.07281343 |
| hsa-miR-6842-5p (miRBase21_mature_hsa) | -2.140 | 0.526 | 2.6426933 | 1.545171 |
| hsa-miR-330-5p (miRBase21_mature_hsa) | -2.408 | 0.527 | 0.46005344 | -0.8075051 |
| hsa-miR-6868-3p (miRBase21_mature_hsa) | 1.675 | 0.529 | -0.09304339 | 0.6509879 |
| hsa-miR-421 (miRBase21_mature_hsa) | -2.665 | 0.530 | 0.6354909 | -0.778749 |
| hsa-miR-6513-3p (miRBase21_mature_hsa) | 1.846 | 0.531 | -0.34085646 | 0.54354274 |
| hsa-miR-24-3p (miRBase21_mature_hsa) | -1.397 | 0.531 | 0.6259456 | 0.1438077 |
| hsa-miR-1306-5p (miRBase21_mature_hsa) | -1.956 | 0.532 | 0.55281323 | -0.4149231 |
| hsa-miR-29b-3p (miRBase21_mature_hsa) | -1.956 | 0.532 | 0.5346473 | -0.43308905 |
| hsa-miR-574-5p (miRBase21_mature_hsa) | -3.030 | 0.535 | 1.7681024 | 0.16859658 |
| hsa-miR-197-3p (miRBase21_mature_hsa) | -1.894 | 0.535 | 0.39467964 | -0.5263886 |
| hsa-miR-501-3p (miRBase21_mature_hsa) | -2.116 | 0.538 | -0.16368842 | -1.2448831 |
| hsa-miR-128-3p (miRBase21_mature_hsa) | -1.375 | 0.540 | 0.631931 | 0.17277433 |
| hsa-miR-6741-5p (miRBase21_mature_hsa) | -2.655 | 0.541 | 0.22704998 | -1.1814709 |
| hsa-miR-7706 (miRBase21_mature_hsa) | 1.458 | 0.542 | 0.6647384 | 1.209143 |
| hsa-miR-548h-3p (miRBase21_mature_hsa) | 1.622 | 0.544 | -0.2054638 | 0.49256244 |
| hsa-miR-365a-5p (miRBase21_mature_hsa) | 1.622 | 0.544 | -0.46113005 | 0.2368961 |
| hsa-miR-625-3p (miRBase21_mature_hsa) | 1.342 | 0.549 | 0.16315256 | 0.58776325 |
| hsa-miR-4433b-3p (miRBase21_mature_hsa) | -1.989 | 0.552 | 0.09962652 | -0.8922033 |
| hsa-miR-200a-5p (miRBase21_mature_hsa) | -2.191 | 0.553 | 1.396257 | 0.26493326 |
| hsa-miR-2110 (miRBase21_mature_hsa) | -1.893 | 0.555 | 0.11585583 | -0.80465037 |
| hsa-miR-942-5p (miRBase21_mature_hsa) | -2.146 | 0.555 | 0.17402656 | -0.9275035 |

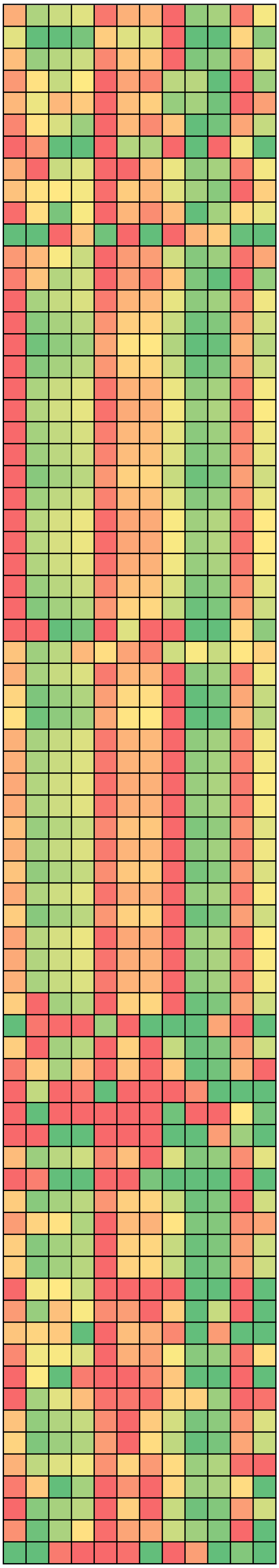

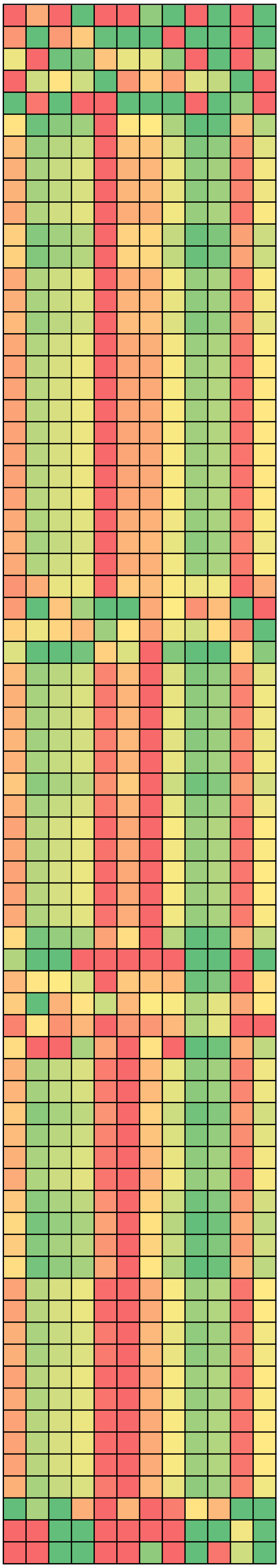

| | | | | |
|---|---|---|---|---|
| hsa-miR-1290 (miRBase21_mature_hsa) | -2.069 | 0.559 | 1.4150056 | 0.3662298 |
| hsa-miR-6721-5p (miRBase21_mature_hsa) | -2.131 | 0.562 | -0.73370683 | -1.824986 |
| hsa-miR-877-5p (miRBase21_mature_hsa) | 1.818 | 0.564 | -0.19861618 | 0.6635877 |
| hsa-miR-320c (miRBase21_mature_hsa) | 1.600 | 0.566 | -0.3961102 | 0.28191844 |
| hsa-miR-939-5p (miRBase21_mature_hsa) | -2.144 | 0.566 | 0.33366087 | -0.76692474 |
| hsa-miR-337-3p (miRBase21_mature_hsa) | -1.703 | 0.567 | 0.032121174 | -0.7356152 |
| hsa-miR-556-5p (miRBase21_mature_hsa) | -1.703 | 0.567 | 0.5879173 | -0.17981902 |
| hsa-miR-6514-3p (miRBase21_mature_hsa) | -1.703 | 0.567 | 0.8157102 | 0.04797383 |
| hsa-miR-548ar-3p (miRBase21_mature_hsa) | -1.703 | 0.567 | 0.7633219 | -0.004414446 |
| hsa-miR-544b (miRBase21_mature_hsa) | -1.703 | 0.567 | 0.9072192 | 0.1394828 |
| hsa-miR-6766-5p (miRBase21_mature_hsa) | -1.703 | 0.567 | 0.33866137 | -0.42907503 |
| hsa-miR-6877-5p (miRBase21_mature_hsa) | -1.703 | 0.567 | 0.3070427 | -0.46069363 |
| hsa-miR-6753-3p (miRBase21_mature_hsa) | -1.703 | 0.567 | 0.9043079 | 0.13657159 |
| hsa-miR-3124-5p (miRBase21_mature_hsa) | -1.703 | 0.567 | 0.8052204 | 0.037484042 |
| hsa-miR-651-5p (miRBase21_mature_hsa) | -1.703 | 0.567 | 0.65395236 | -0.11378402 |
| hsa-miR-1277-3p (miRBase21_mature_hsa) | -1.703 | 0.567 | 0.936035 | 0.16829859 |
| hsa-miR-3133 (miRBase21_mature_hsa) | -1.703 | 0.567 | 1.0102267 | 0.2424904 |
| hsa-miR-3180-5p (miRBase21_mature_hsa) | -1.703 | 0.567 | 1.0102267 | 0.2424904 |
| hsa-miR-4446-5p (miRBase21_mature_hsa) | -1.703 | 0.567 | 1.0102267 | 0.2424904 |
| hsa-miR-4474-3p (miRBase21_mature_hsa) | -1.703 | 0.567 | 1.0102267 | 0.2424904 |
| hsa-miR-4677-5p (miRBase21_mature_hsa) | -1.703 | 0.567 | 1.0102267 | 0.2424904 |
| hsa-miR-4754 (miRBase21_mature_hsa) | -1.703 | 0.567 | 1.0102267 | 0.2424904 |
| hsa-miR-4779 (miRBase21_mature_hsa) | -1.703 | 0.567 | 1.0102267 | 0.2424904 |
| hsa-miR-548k (miRBase21_mature_hsa) | -1.703 | 0.567 | 0.8948349 | 0.12709844 |
| hsa-miR-6727-5p (miRBase21_mature_hsa) | -1.703 | 0.567 | 0.7755078 | 0.007771426 |
| hsa-miR-6882-5p (miRBase21_mature_hsa) | -1.703 | 0.567 | 0.9065199 | 0.13878354 |
| hsa-miR-126-5p (miRBase21_mature_hsa) | -1.291 | 0.568 | 0.87755316 | 0.5095523 |
| hsa-miR-483-5p (miRBase21_mature_hsa) | 1.783 | 0.569 | -1.0110505 | -0.17678283 |
| hsa-miR-30a-5p (miRBase21_mature_hsa) | -1.581 | 0.569 | 0.014973735 | -0.6456824 |
| hsa-miR-1260b (miRBase21_mature_hsa) | 1.517 | 0.570 | -1.2340811 | -0.6329069 |
| hsa-miR-4433a-3p (miRBase21_mature_hsa) | 1.517 | 0.570 | -0.090809524 | 0.51036465 |
| hsa-miR-4510 (miRBase21_mature_hsa) | 1.517 | 0.570 | 0.06986595 | 0.6710401 |
| hsa-miR-4683 (miRBase21_mature_hsa) | 1.517 | 0.570 | 0.051848035 | 0.6530222 |
| hsa-miR-4726-5p (miRBase21_mature_hsa) | 1.517 | 0.570 | -0.004531801 | 0.5966423 |
| hsa-miR-4688 (miRBase21_mature_hsa) | 1.517 | 0.570 | 0.0658872 | 0.6670614 |
| hsa-miR-24-1-5p (miRBase21_mature_hsa) | 1.517 | 0.570 | -0.2892497 | 0.3119245 |
| hsa-miR-4677-3p (miRBase21_mature_hsa) | 1.517 | 0.570 | 0.05990833 | 0.6610825 |
| hsa-miR-1236-5p (miRBase21_mature_hsa) | 1.517 | 0.570 | 0.27016342 | 0.8713376 |
| hsa-miR-1258 (miRBase21_mature_hsa) | 1.517 | 0.570 | 0.19597162 | 0.7971458 |
| hsa-miR-3192-5p (miRBase21_mature_hsa) | 1.517 | 0.570 | 0.27016342 | 0.8713376 |
| hsa-miR-6793-3p (miRBase21_mature_hsa) | 1.517 | 0.570 | 0.27016342 | 0.8713376 |
| hsa-miR-7849-3p (miRBase21_mature_hsa) | 1.517 | 0.570 | 0.18275404 | 0.78392816 |
| hsa-miR-1226-3p (miRBase21_mature_hsa) | 1.517 | 0.570 | -0.55760026 | 0.043573912 |
| hsa-miR-374a-3p (miRBase21_mature_hsa) | 2.404 | 0.573 | 0.1497397 | 1.4150361 |
| hsa-miR-146b-5p (miRBase21_mature_hsa) | -1.335 | 0.577 | 0.54306424 | 0.12599574 |
| hsa-miR-486-5p (miRBase21_mature_hsa) | 1.280 | 0.581 | -0.2831221 | 0.0730882 |
| hsa-miR-17-5p (miRBase21_mature_hsa) | -1.393 | 0.584 | 1.5643898 | 1.0860431 |
| hsa-miR-3928-3p (miRBase21_mature_hsa) | -1.761 | 0.587 | 1.6093713 | 0.79282445 |
| hsa-miR-10a-3p (miRBase21_mature_hsa) | 1.469 | 0.590 | 0.052227505 | 0.60739654 |
| hsa-miR-1270 (miRBase21_mature_hsa) | 1.469 | 0.590 | 0.022168458 | 0.5773375 |
| hsa-miR-136-3p (miRBase21_mature_hsa) | 1.469 | 0.590 | -0.30798918 | 0.24717987 |
| hsa-miR-128-1-5p (miRBase21_mature_hsa) | 1.469 | 0.590 | -0.094751894 | 0.46041715 |
| hsa-miR-516b-5p (miRBase21_mature_hsa) | 1.469 | 0.590 | 0.08029057 | 0.6354596 |
| hsa-miR-490-3p (miRBase21_mature_hsa) | 1.469 | 0.590 | 0.15352532 | 0.70869434 |
| hsa-miR-6735-5p (miRBase21_mature_hsa) | 1.469 | 0.590 | -0.29537088 | 0.2597982 |
| hsa-miR-6859-5p (miRBase21_mature_hsa) | 1.469 | 0.590 | -0.5406952 | 0.014473832 |
| hsa-miR-29c-5p (miRBase21_mature_hsa) | 1.469 | 0.590 | -0.3351275 | 0.22004154 |
| hsa-miR-548e-3p (miRBase21_mature_hsa) | 1.469 | 0.590 | -0.59849405 | -0.043325033 |
| hsa-miR-26a-2-3p (miRBase21_mature_hsa) | 1.469 | 0.590 | 0.27716422 | 0.83233327 |
| hsa-miR-3168 (miRBase21_mature_hsa) | 1.469 | 0.590 | 0.27716422 | 0.83233327 |
| hsa-miR-3591-5p (miRBase21_mature_hsa) | 1.469 | 0.590 | 0.085709676 | 0.64087874 |
| hsa-miR-3614-5p (miRBase21_mature_hsa) | 1.469 | 0.590 | 0.08898724 | 0.6441563 |
| hsa-miR-3675-3p (miRBase21_mature_hsa) | 1.469 | 0.590 | 0.27716422 | 0.83233327 |
| hsa-miR-4511 (miRBase21_mature_hsa) | 1.469 | 0.590 | 0.18658611 | 0.7417552 |
| hsa-miR-506-3p (miRBase21_mature_hsa) | 1.469 | 0.590 | 0.27716422 | 0.83233327 |
| hsa-miR-550a-3p (miRBase21_mature_hsa) | 1.469 | 0.590 | 0.27716422 | 0.83233327 |
| hsa-miR-551b-3p (miRBase21_mature_hsa) | 1.469 | 0.590 | 0.27716422 | 0.83233327 |
| hsa-miR-6791-5p (miRBase21_mature_hsa) | 1.469 | 0.590 | 0.07142002 | 0.62658906 |
| hsa-let-7b-3p (miRBase21_mature_hsa) | 1.914 | 0.603 | -1.0684953 | -0.1316144 |
| hsa-miR-1273h-5p (miRBase21_mature_hsa) | -1.975 | 0.603 | 1.1368153 | 0.15482722 |
| hsa-miR-30b-5p (miRBase21_mature_hsa) | -1.921 | 0.603 | 0.76034874 | -0.18136625 |

| | | | | |
|---|---|---|---|---|
| hsa-miR-328-3p (miRBase21_mature_hsa) | -1.277 | 0.605 | 0.50144255 | 0.14845903 |
| hsa-miR-1229-3p (miRBase21_mature_hsa) | -1.701 | 0.614 | 0.93078226 | 0.16479509 |
| hsa-miR-484 (miRBase21_mature_hsa) | -1.223 | 0.623 | 0.33828497 | 0.04827588 |
| hsa-miR-199b-5p (miRBase21_mature_hsa) | -2.037 | 0.625 | 1.189773 | 0.16319266 |
| hsa-miR-889-3p (miRBase21_mature_hsa) | -2.040 | 0.627 | -0.0972569 | -1.1255862 |
| hsa-let-7e-5p (miRBase21_mature_hsa) | 1.379 | 0.634 | 0.22272292 | 0.6861961 |
| hsa-miR-5009-5p (miRBase21_mature_hsa) | 1.808 | 0.638 | 0.5370616 | 1.3915662 |
| hsa-let-7i-5p (miRBase21_mature_hsa) | -1.383 | 0.643 | 0.7979815 | 0.33063135 |
| hsa-miR-16-2-3p (miRBase21_mature_hsa) | 1.174 | 0.644 | 0.19322774 | 0.4250498 |
| hsa-miR-505-5p (miRBase21_mature_hsa) | -1.457 | 0.646 | 0.53339916 | -0.00912912 |
| hsa-miR-1254 (miRBase21_mature_hsa) | -1.783 | 0.646 | 1.5165224 | 0.6820506 |
| hsa-miR-504-5p (miRBase21_mature_hsa) | 1.623 | 0.649 | 0.24087235 | 0.9396386 |
| hsa-miR-423-5p (miRBase21_mature_hsa) | 1.255 | 0.650 | -0.049137004 | 0.27842015 |
| hsa-miR-329-5p (miRBase21_mature_hsa) | 1.633 | 0.650 | 0.23779401 | 0.9454356 |
| hsa-miR-576-5p (miRBase21_mature_hsa) | 1.447 | 0.654 | -0.4808796 | 0.05209407 |
| hsa-miR-223-5p (miRBase21_mature_hsa) | -1.288 | 0.655 | 0.8914507 | 0.52595913 |
| hsa-miR-629-5p (miRBase21_mature_hsa) | 1.258 | 0.661 | 0.46565843 | 0.7966505 |
| hsa-miR-323a-5p (miRBase21_mature_hsa) | 1.540 | 0.666 | -0.83879745 | -0.21594751 |
| hsa-miR-96-5p (miRBase21_mature_hsa) | -1.548 | 0.666 | 0.8898243 | 0.25906202 |
| hsa-miR-32-5p (miRBase21_mature_hsa) | -1.905 | 0.667 | 0.3280607 | -0.6016396 |
| hsa-miR-125b-5p (miRBase21_mature_hsa) | -1.544 | 0.667 | -0.62392956 | -1.2503347 |
| hsa-miR-27b-5p (miRBase21_mature_hsa) | -1.672 | 0.668 | 0.78390026 | 0.042185303 |
| hsa-miR-221-5p (miRBase21_mature_hsa) | 1.609 | 0.671 | -0.52809036 | 0.15770133 |
| hsa-miR-151a-3p (miRBase21_mature_hsa) | -1.411 | 0.676 | 0.69485927 | 0.19811809 |
| hsa-miR-1255b-5p (miRBase21_mature_hsa) | -1.678 | 0.678 | 0.20431781 | -0.54239434 |
| hsa-miR-30c-5p (miRBase21_mature_hsa) | -1.214 | 0.683 | 0.7998853 | 0.51973665 |
| hsa-miR-1304-5p (miRBase21_mature_hsa) | -1.620 | 0.686 | 0.72139823 | 0.025811601 |
| hsa-miR-132-3p (miRBase21_mature_hsa) | -1.580 | 0.686 | 1.4351308 | 0.7756111 |
| hsa-miR-326 (miRBase21_mature_hsa) | -1.651 | 0.687 | 0.5863842 | -0.13705505 |
| hsa-miR-3909 (miRBase21_mature_hsa) | 1.695 | 0.687 | 0.39174655 | 1.1532544 |
| hsa-miR-93-3p (miRBase21_mature_hsa) | 1.558 | 0.689 | 0.28627676 | 0.9260634 |
| hsa-miR-4446-3p (miRBase21_mature_hsa) | -1.399 | 0.691 | 0.38722298 | -0.09749309 |
| hsa-miR-361-3p (miRBase21_mature_hsa) | -1.515 | 0.692 | 0.186119 | -0.413641 |
| hsa-miR-139-5p (miRBase21_mature_hsa) | -1.259 | 0.693 | 0.7725932 | 0.4398594 |
| hsa-miR-1271-5p (miRBase21_mature_hsa) | -1.544 | 0.698 | 1.556427 | 0.9293461 |
| hsa-miR-424-3p (miRBase21_mature_hsa) | -1.740 | 0.702 | -0.040755503 | -0.84012944 |
| hsa-miR-139-3p (miRBase21_mature_hsa) | 1.231 | 0.704 | 0.09082594 | 0.39008436 |
| hsa-miR-941 (miRBase21_mature_hsa) | -1.255 | 0.717 | 0.60683596 | 0.27958307 |
| hsa-miR-550a-5p (miRBase21_mature_hsa) | -1.498 | 0.719 | 0.99200636 | 0.4087755 |
| hsa-miR-206 (miRBase21_mature_hsa) | 1.676 | 0.726 | -0.5607603 | 0.18386044 |
| hsa-miR-378c (miRBase21_mature_hsa) | -1.363 | 0.726 | 0.53227395 | 0.08535772 |
| hsa-miR-664a-5p (miRBase21_mature_hsa) | -1.486 | 0.726 | 0.9503967 | 0.3786764 |
| hsa-miR-4473 (miRBase21_mature_hsa) | -1.656 | 0.727 | 0.17179632 | -0.55604964 |
| hsa-miR-659-5p (miRBase21_mature_hsa) | -1.336 | 0.728 | 1.1166221 | 0.6983712 |
| hsa-miR-6842-3p (miRBase21_mature_hsa) | -1.275 | 0.729 | 0.56788266 | 0.21709163 |
| hsa-let-7f-5p (miRBase21_mature_hsa) | -1.319 | 0.732 | 0.743507 | 0.34447855 |
| hsa-miR-215-5p (miRBase21_mature_hsa) | 1.475 | 0.735 | -0.60889703 | -0.0483394 |
| hsa-let-7c-5p (miRBase21_mature_hsa) | 1.258 | 0.736 | 0.021064138 | 0.352656 |
| hsa-miR-409-3p (miRBase21_mature_hsa) | -1.187 | 0.742 | 0.32925797 | 0.08226853 |
| hsa-miR-503-5p (miRBase21_mature_hsa) | -1.468 | 0.743 | 0.57467234 | 0.020657275 |
| hsa-miR-4433b-5p (miRBase21_mature_hsa) | -1.239 | 0.744 | 0.28922662 | -0.020329343 |
| hsa-miR-3064-5p (miRBase21_mature_hsa) | -1.568 | 0.746 | 0.7419995 | 0.09279586 |
| hsa-miR-329-3p (miRBase21_mature_hsa) | -1.590 | 0.751 | 0.7847866 | 0.116183855 |
| hsa-miR-186-5p (miRBase21_mature_hsa) | 1.127 | 0.752 | 0.42864174 | 0.60076636 |
| hsa-miR-223-3p (miRBase21_mature_hsa) | -1.196 | 0.757 | 0.7032603 | 0.44489214 |
| hsa-miR-1287-5p (miRBase21_mature_hsa) | -1.454 | 0.769 | 0.9860256 | 0.44576395 |
| hsa-miR-194-5p (miRBase21_mature_hsa) | -1.364 | 0.775 | 0.031071221 | -0.4170082 |
| hsa-miR-130b-5p (miRBase21_mature_hsa) | -1.243 | 0.779 | 0.34966582 | 0.035271425 |
| hsa-miR-92a-3p (miRBase21_mature_hsa) | -1.104 | 0.780 | 0.20147412 | 0.05834788 |
| hsa-miR-150-3p (miRBase21_mature_hsa) | -1.455 | 0.786 | -9.55E-04 | -0.5420083 |
| hsa-miR-3173-5p (miRBase21_mature_hsa) | -1.272 | 0.789 | 0.54159075 | 0.19436052 |
| hsa-miR-660-5p (miRBase21_mature_hsa) | -1.353 | 0.791 | 0.6004202 | 0.16441157 |
| hsa-miR-3913-5p (miRBase21_mature_hsa) | -1.275 | 0.794 | 0.13112476 | -0.21893945 |
| hsa-miR-769-5p (miRBase21_mature_hsa) | -1.543 | 0.795 | -0.0982096 | -0.7239299 |
| hsa-miR-6505-3p (miRBase21_mature_hsa) | -1.225 | 0.796 | 0.82521904 | 0.5322977 |
| hsa-miR-6514-5p (miRBase21_mature_hsa) | -1.225 | 0.796 | 0.73595333 | 0.44303197 |
| hsa-miR-320d (miRBase21_mature_hsa) | 1.361 | 0.798 | -0.6215908 | -0.17739558 |
| hsa-miR-548l (miRBase21_mature_hsa) | -1.342 | 0.799 | 1.4065667 | 0.9825859 |
| hsa-miR-135a-3p (miRBase21_mature_hsa) | -1.330 | 0.801 | 0.88074535 | 0.46884915 |
| hsa-miR-615-3p (miRBase21_mature_hsa) | 1.265 | 0.802 | 0.12652709 | 0.46590948 |
| hsa-miR-27b-3p (miRBase21_mature_hsa) | -1.203 | 0.802 | 0.39802516 | 0.13095973 |

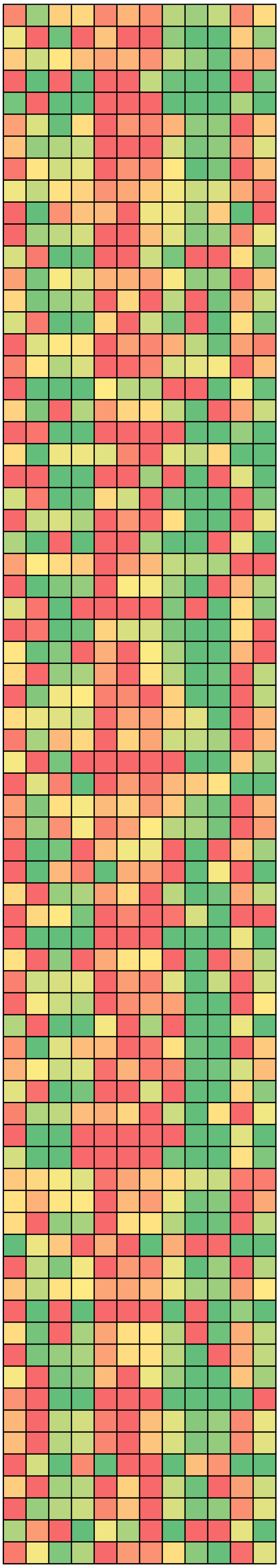

| | | | | |
|---|---|---|---|---|
| hsa-miR-25-3p (miRBase21_mature_hsa) | -1.105 | 0.803 | -0.018281536 | -0.16283105 |
| hsa-miR-146a-3p (miRBase21_mature_hsa) | -1.206 | 0.813 | 0.32376197 | 0.0532579 |
| hsa-miR-582-3p (miRBase21_mature_hsa) | -1.278 | 0.819 | -0.22799863 | -0.58201367 |
| hsa-miR-95-3p (miRBase21_mature_hsa) | -1.179 | 0.821 | 0.003991462 | -0.23407374 |
| hsa-miR-214-5p (miRBase21_mature_hsa) | 1.291 | 0.824 | 0.47870836 | 0.84670216 |
| hsa-miR-541-3p (miRBase21_mature_hsa) | -1.235 | 0.824 | 1.0653801 | 0.7613209 |
| hsa-miR-5189-5p (miRBase21_mature_hsa) | -1.418 | 0.824 | 0.37517184 | -0.12853758 |
| hsa-miR-7110-3p (miRBase21_mature_hsa) | -1.284 | 0.826 | 0.54793715 | 0.18706055 |
| hsa-miR-5187-5p (miRBase21_mature_hsa) | -1.187 | 0.831 | -0.42617154 | -0.6730878 |
| hsa-miR-3191-3p (miRBase21_mature_hsa) | -1.293 | 0.833 | 0.55887073 | 0.18820468 |
| hsa-miR-142-5p (miRBase21_mature_hsa) | -1.154 | 0.839 | 0.1471596 | -0.059636917 |
| hsa-miR-28-5p (miRBase21_mature_hsa) | -1.159 | 0.840 | 1.3428491 | 1.1301738 |
| hsa-miR-362-5p (miRBase21_mature_hsa) | 1.222 | 0.843 | 0.44588768 | 0.7355188 |
| hsa-miR-363-5p (miRBase21_mature_hsa) | 1.255 | 0.848 | 0.26553538 | 0.5934698 |
| hsa-miR-196b-5p (miRBase21_mature_hsa) | 1.270 | 0.849 | -0.23796506 | 0.106678896 |
| hsa-miR-1343-3p (miRBase21_mature_hsa) | -1.206 | 0.858 | 0.029571628 | -0.24041438 |
| hsa-miR-1294 (miRBase21_mature_hsa) | -1.247 | 0.860 | -0.32233244 | -0.64132744 |
| hsa-miR-143-3p (miRBase21_mature_hsa) | -1.123 | 0.860 | -0.12996402 | -0.29795322 |
| hsa-miR-548j-3p (miRBase21_mature_hsa) | -1.217 | 0.874 | 0.56443757 | 0.28089973 |
| hsa-miR-1255a (miRBase21_mature_hsa) | -1.281 | 0.876 | 1.0085102 | 0.650996 |
| hsa-miR-1285-3p (miRBase21_mature_hsa) | -1.113 | 0.877 | -0.38706404 | -0.54110014 |
| hsa-miR-106b-5p (miRBase21_mature_hsa) | -1.113 | 0.877 | -0.9452139 | -1.0992501 |
| hsa-miR-148b-5p (miRBase21_mature_hsa) | -1.168 | 0.883 | 1.2165958 | 0.99261487 |
| hsa-miR-937-3p (miRBase21_mature_hsa) | 1.186 | 0.885 | 0.46249074 | 0.7089286 |
| hsa-miR-3605-3p (miRBase21_mature_hsa) | 1.131 | 0.885 | 0.84439254 | 1.0214779 |
| hsa-miR-92b-5p (miRBase21_mature_hsa) | -1.111 | 0.885 | 0.19789964 | 0.046035305 |
| hsa-miR-628-3p (miRBase21_mature_hsa) | -1.158 | 0.886 | -1.3187813 | -1.5306774 |
| hsa-miR-6866-5p (miRBase21_mature_hsa) | -1.245 | 0.888 | 0.7980017 | 0.48239326 |
| hsa-miR-4750-5p (miRBase21_mature_hsa) | 1.211 | 0.888 | 0.3423066 | 0.6185058 |
| hsa-miR-140-5p (miRBase21_mature_hsa) | -1.238 | 0.889 | -0.3337208 | -0.64125836 |
| hsa-miR-1227-3p (miRBase21_mature_hsa) | -1.166 | 0.899 | 0.6808849 | 0.45948347 |
| hsa-miR-409-5p (miRBase21_mature_hsa) | -1.139 | 0.900 | 0.53531814 | 0.34746236 |
| hsa-miR-99b-3p (miRBase21_mature_hsa) | 1.160 | 0.907 | 0.44870484 | 0.6625525 |
| hsa-miR-532-5p (miRBase21_mature_hsa) | -1.045 | 0.913 | 0.098161235 | 0.03527476 |
| hsa-miR-4786-5p (miRBase21_mature_hsa) | -1.121 | 0.913 | 0.689178 | 0.5249194 |
| hsa-miR-6780a-5p (miRBase21_mature_hsa) | -1.121 | 0.913 | 0.3838891 | 0.21963051 |
| hsa-miR-548d-5p (miRBase21_mature_hsa) | -1.121 | 0.913 | 0.20325519 | 0.038996637 |
| hsa-let-7a-5p (miRBase21_mature_hsa) | -1.082 | 0.919 | 0.5807424 | 0.4677053 |
| hsa-miR-485-5p (miRBase21_mature_hsa) | 1.056 | 0.922 | 0.31981093 | 0.3980371 |
| hsa-miR-7-5p (miRBase21_mature_hsa) | -1.066 | 0.925 | 0.7762186 | 0.68373734 |
| hsa-miR-323b-3p (miRBase21_mature_hsa) | -1.133 | 0.932 | -0.25284803 | -0.43246573 |
| hsa-miR-6505-5p (miRBase21_mature_hsa) | -1.068 | 0.934 | 0.8629308 | 0.7677227 |
| hsa-let-7d-3p (miRBase21_mature_hsa) | -1.040 | 0.934 | 0.29545346 | 0.23858722 |
| hsa-miR-184 (miRBase21_mature_hsa) | -1.075 | 0.934 | -0.034004517 | -0.13806365 |
| hsa-miR-654-5p (miRBase21_mature_hsa) | -1.064 | 0.935 | 0.45454273 | 0.36548042 |
| hsa-miR-26a-1-3p (miRBase21_mature_hsa) | -1.085 | 0.938 | 0.91139245 | 0.793139 |
| hsa-miR-3158-3p (miRBase21_mature_hsa) | 1.035 | 0.939 | 0.77752614 | 0.8270883 |
| hsa-miR-499a-5p (miRBase21_mature_hsa) | -1.079 | 0.940 | -0.39659557 | -0.505998 |
| hsa-miR-7854-3p (miRBase21_mature_hsa) | -1.079 | 0.940 | 0.4300805 | 0.3206781 |
| hsa-miR-3615 (miRBase21_mature_hsa) | 1.030 | 0.945 | 0.035177518 | 0.07719878 |
| hsa-miR-141-3p (miRBase21_mature_hsa) | -1.034 | 0.945 | -0.017556215 | -0.065864965 |
| hsa-miR-92a-1-5p (miRBase21_mature_hsa) | -1.034 | 0.945 | -0.38981178 | -0.43812054 |
| hsa-miR-106a-5p (miRBase21_mature_hsa) | -1.034 | 0.945 | -0.08465499 | -0.13296373 |
| hsa-miR-324-3p (miRBase21_mature_hsa) | -1.034 | 0.945 | 0.026794415 | -0.021514324 |
| hsa-miR-5189-3p (miRBase21_mature_hsa) | -1.034 | 0.945 | 0.20766363 | 0.1593549 |
| hsa-miR-618 (miRBase21_mature_hsa) | -1.034 | 0.945 | 0.20766363 | 0.1593549 |
| hsa-miR-6802-3p (miRBase21_mature_hsa) | -1.034 | 0.945 | 0.20766363 | 0.1593549 |
| hsa-miR-4745-5p (miRBase21_mature_hsa) | -1.034 | 0.945 | 0.15425366 | 0.105944924 |
| hsa-miR-3175 (miRBase21_mature_hsa) | -1.034 | 0.945 | -0.15644518 | -0.2047539 |
| hsa-miR-4467 (miRBase21_mature_hsa) | -1.034 | 0.945 | -0.03365313 | -0.08196188 |
| hsa-miR-6857-3p (miRBase21_mature_hsa) | -1.034 | 0.945 | 0.07068319 | 0.02237445 |
| hsa-miR-144-5p (miRBase21_mature_hsa) | -1.034 | 0.945 | -0.78573525 | -0.83404404 |
| hsa-miR-4646-3p (miRBase21_mature_hsa) | -1.034 | 0.945 | -0.04760482 | -0.09591355 |
| hsa-miR-4433a-5p (miRBase21_mature_hsa) | -1.034 | 0.945 | 0.064458594 | 0.01614985 |
| hsa-miR-487a-5p (miRBase21_mature_hsa) | -1.034 | 0.945 | -0.09224679 | -0.14055555 |
| hsa-miR-3130-3p (miRBase21_mature_hsa) | -1.034 | 0.945 | 0.15249917 | 0.10419043 |
| hsa-miR-1238-3p (miRBase21_mature_hsa) | -1.034 | 0.945 | 0.27352512 | 0.22521639 |
| hsa-miR-1976 (miRBase21_mature_hsa) | -1.034 | 0.945 | 0.27352512 | 0.22521639 |
| hsa-miR-3164 (miRBase21_mature_hsa) | -1.034 | 0.945 | -0.03812673 | -0.086435474 |
| hsa-miR-34a-5p (miRBase21_mature_hsa) | -1.034 | 0.945 | 0.27352512 | 0.22521639 |
| hsa-miR-3911 (miRBase21_mature_hsa) | -1.034 | 0.945 | 0.27352512 | 0.22521639 |

| | | | | |
|---|---|---|---|---|
| hsa-miR-4497 (miRBase21_mature_hsa) | -1.034 | 0.945 | 0.27352512 | 0.22521639 |
| hsa-miR-4508 (miRBase21_mature_hsa) | -1.034 | 0.945 | -0.11419358 | -0.16250232 |
| hsa-miR-598-3p (miRBase21_mature_hsa) | -1.034 | 0.945 | 0.17448121 | 0.12617248 |
| hsa-miR-6736-3p (miRBase21_mature_hsa) | -1.034 | 0.945 | 0.1742383 | 0.12592956 |
| hsa-miR-6750-3p (miRBase21_mature_hsa) | -1.034 | 0.945 | 0.27352512 | 0.22521639 |
| hsa-miR-6821-3p (miRBase21_mature_hsa) | -1.034 | 0.945 | 0.27352512 | 0.22521639 |
| hsa-miR-933 (miRBase21_mature_hsa) | -1.034 | 0.945 | 0.19933331 | 0.15102458 |
| hsa-miR-3148 (miRBase21_mature_hsa) | -1.034 | 0.945 | 0.2497263 | 0.20141755 |
| hsa-miR-3613-3p (miRBase21_mature_hsa) | -1.034 | 0.945 | 0.2497263 | 0.20141755 |
| hsa-miR-487b-5p (miRBase21_mature_hsa) | -1.034 | 0.945 | 0.2497263 | 0.20141755 |
| hsa-miR-548aq-3p (miRBase21_mature_hsa) | -1.034 | 0.945 | 0.2497263 | 0.20141755 |
| hsa-miR-708-3p (miRBase21_mature_hsa) | -1.034 | 0.945 | -0.25021568 | -0.2985244 |
| hsa-miR-194-3p (miRBase21_mature_hsa) | -1.034 | 0.945 | 0.26868057 | 0.22037184 |
| hsa-miR-5582-3p (miRBase21_mature_hsa) | -1.034 | 0.945 | 0.26868057 | 0.22037184 |
| hsa-miR-34c-5p (miRBase21_mature_hsa) | -1.034 | 0.945 | 0.2813972 | 0.23308845 |
| hsa-miR-452-5p (miRBase21_mature_hsa) | -1.034 | 0.945 | -0.108285055 | -0.15659381 |
| hsa-miR-4784 (miRBase21_mature_hsa) | -1.034 | 0.945 | 0.2813972 | 0.23308845 |
| hsa-miR-488-3p (miRBase21_mature_hsa) | -1.034 | 0.945 | 0.2813972 | 0.23308845 |
| hsa-miR-5192 (miRBase21_mature_hsa) | -1.034 | 0.945 | 0.2813972 | 0.23308845 |
| hsa-miR-5196-3p (miRBase21_mature_hsa) | -1.034 | 0.945 | 0.2813972 | 0.23308845 |
| hsa-miR-668-3p (miRBase21_mature_hsa) | -1.034 | 0.945 | -0.051112555 | -0.0994213 |
| hsa-miR-6827-3p (miRBase21_mature_hsa) | -1.034 | 0.945 | 0.2813972 | 0.23308845 |
| hsa-miR-642a-3p (miRBase21_mature_hsa) | -1.034 | 0.945 | -0.67489845 | -0.7232072 |
| hsa-miR-1277-5p (miRBase21_mature_hsa) | -1.034 | 0.945 | 0.015376627 | -0.032932103 |
| hsa-miR-205-5p (miRBase21_mature_hsa) | -1.034 | 0.945 | -0.13765281 | -0.18596154 |
| hsa-miR-3150b-3p (miRBase21_mature_hsa) | -1.034 | 0.945 | 0.22894451 | 0.18063577 |
| hsa-miR-4686 (miRBase21_mature_hsa) | -1.034 | 0.945 | 0.30313632 | 0.2548276 |
| hsa-miR-4772-5p (miRBase21_mature_hsa) | -1.034 | 0.945 | 0.30313632 | 0.2548276 |
| hsa-miR-4787-3p (miRBase21_mature_hsa) | -1.034 | 0.945 | 0.30313632 | 0.2548276 |
| hsa-miR-550a-3-5p (miRBase21_mature_hsa) | -1.034 | 0.945 | 0.30313632 | 0.2548276 |
| hsa-miR-627-3p (miRBase21_mature_hsa) | -1.034 | 0.945 | 0.21572691 | 0.16741818 |
| hsa-miR-6765-5p (miRBase21_mature_hsa) | -1.034 | 0.945 | 0.22894451 | 0.18063577 |
| hsa-miR-6812-5p (miRBase21_mature_hsa) | -1.034 | 0.945 | 0.30313632 | 0.2548276 |
| hsa-miR-6837-5p (miRBase21_mature_hsa) | -1.034 | 0.945 | 0.30313632 | 0.2548276 |
| hsa-miR-6849-3p (miRBase21_mature_hsa) | -1.034 | 0.945 | 0.30313632 | 0.2548276 |
| hsa-miR-935 (miRBase21_mature_hsa) | -1.034 | 0.945 | 0.30313632 | 0.2548276 |
| hsa-miR-103a-2-5p (miRBase21_mature_hsa) | -1.034 | 0.945 | 0.26589125 | 0.21758252 |
| hsa-miR-1250-5p (miRBase21_mature_hsa) | -1.034 | 0.945 | 0.13433883 | 0.08603009 |
| hsa-miR-299-5p (miRBase21_mature_hsa) | -1.034 | 0.945 | 0.26589125 | 0.21758252 |
| hsa-miR-30c-2-3p (miRBase21_mature_hsa) | -1.034 | 0.945 | -0.07772885 | -0.12603761 |
| hsa-miR-3190-3p (miRBase21_mature_hsa) | -1.034 | 0.945 | 0.26589125 | 0.21758252 |
| hsa-miR-4654 (miRBase21_mature_hsa) | -1.034 | 0.945 | -0.34231177 | -0.3906205 |
| hsa-miR-4659b-3p (miRBase21_mature_hsa) | -1.034 | 0.945 | 0.19169945 | 0.14339072 |
| hsa-miR-6767-5p (miRBase21_mature_hsa) | -1.034 | 0.945 | -0.20858513 | -0.25689387 |
| hsa-miR-7702 (miRBase21_mature_hsa) | -1.034 | 0.945 | 0.26589125 | 0.21758252 |
| hsa-miR-4429 (miRBase21_mature_hsa) | -1.034 | 0.945 | 0.17950831 | 0.13119957 |
| hsa-miR-4758-3p (miRBase21_mature_hsa) | -1.034 | 0.945 | 0.2537001 | 0.20539138 |
| hsa-miR-615-5p (miRBase21_mature_hsa) | -1.034 | 0.945 | 0.13286318 | 0.08455443 |
| hsa-miR-6733-5p (miRBase21_mature_hsa) | -1.034 | 0.945 | 0.15776919 | 0.10946044 |
| hsa-miR-1185-2-3p (miRBase21_mature_hsa) | -1.034 | 0.945 | 0.30044588 | 0.25213715 |
| hsa-miR-3157-3p (miRBase21_mature_hsa) | -1.034 | 0.945 | 0.18019463 | 0.13188589 |
| hsa-miR-4504 (miRBase21_mature_hsa) | -1.034 | 0.945 | 0.10572929 | 0.05742055 |
| hsa-miR-4521 (miRBase21_mature_hsa) | -1.034 | 0.945 | 0.30044588 | 0.25213715 |
| hsa-miR-509-3p (miRBase21_mature_hsa) | -1.034 | 0.945 | 0.30044588 | 0.25213715 |
| hsa-miR-556-3p (miRBase21_mature_hsa) | -1.034 | 0.945 | 0.30044588 | 0.25213715 |
| hsa-miR-6503-5p (miRBase21_mature_hsa) | -1.034 | 0.945 | 0.30044588 | 0.25213715 |
| hsa-miR-6736-5p (miRBase21_mature_hsa) | -1.034 | 0.945 | 0.30044588 | 0.25213715 |
| hsa-miR-6764-5p (miRBase21_mature_hsa) | -1.034 | 0.945 | 0.30044588 | 0.25213715 |
| hsa-miR-6787-5p (miRBase21_mature_hsa) | -1.034 | 0.945 | 0.30044588 | 0.25213715 |
| hsa-miR-892a (miRBase21_mature_hsa) | -1.034 | 0.945 | 0.30044588 | 0.25213715 |
| hsa-miR-378f (miRBase21_mature_hsa) | -1.034 | 0.945 | 0.25935873 | 0.21105 |
| hsa-miR-4306 (miRBase21_mature_hsa) | -1.034 | 0.945 | -0.036488317 | -0.08479706 |
| hsa-miR-4326 (miRBase21_mature_hsa) | -1.034 | 0.945 | 0.25935873 | 0.21105 |
| hsa-miR-4755-3p (miRBase21_mature_hsa) | -1.034 | 0.945 | 0.25935873 | 0.21105 |
| hsa-miR-550b-3p (miRBase21_mature_hsa) | -1.034 | 0.945 | 0.25935873 | 0.21105 |
| hsa-miR-6885-3p (miRBase21_mature_hsa) | -1.034 | 0.945 | 0.16031486 | 0.11200612 |
| hsa-miR-3691-5p (miRBase21_mature_hsa) | -1.034 | 0.945 | -0.0813823 | -0.12969103 |
| hsa-miR-542-5p (miRBase21_mature_hsa) | -1.034 | 0.945 | 0.27383304 | 0.22552429 |
| hsa-miR-6734-3p (miRBase21_mature_hsa) | -1.034 | 0.945 | 0.14282092 | 0.09451219 |
| hsa-miR-675-3p (miRBase21_mature_hsa) | -1.034 | 0.945 | 0.27383304 | 0.22552429 |
| hsa-miR-7113-5p (miRBase21_mature_hsa) | -1.034 | 0.945 | 0.27383304 | 0.22552429 |

| | | | | |
|---|---|---|---|---|
| hsa-miR-7703 (miRBase21_mature_hsa) | -1.034 | 0.945 | 0.27383304 | 0.22552429 |
| hsa-miR-2278 (miRBase21_mature_hsa) | -1.034 | 0.945 | 0.21463141 | 0.16632266 |
| hsa-miR-6740-5p (miRBase21_mature_hsa) | -1.034 | 0.945 | 0.21463141 | 0.16632266 |
| hsa-miR-6818-5p (miRBase21_mature_hsa) | -1.034 | 0.945 | 0.23644504 | 0.18813631 |
| hsa-miR-1237-3p (miRBase21_mature_hsa) | -1.034 | 0.945 | 0.06895627 | 0.020647524 |
| hsa-miR-1275 (miRBase21_mature_hsa) | -1.034 | 0.945 | 0.267098 | 0.21878928 |
| hsa-miR-1307-5p (miRBase21_mature_hsa) | -1.034 | 0.945 | 0.067872375 | 0.019563634 |
| hsa-miR-195-5p (miRBase21_mature_hsa) | -1.034 | 0.945 | 0.14684677 | 0.09853803 |
| hsa-miR-2116-3p (miRBase21_mature_hsa) | -1.034 | 0.945 | 0.16276169 | 0.11445295 |
| hsa-miR-21-3p (miRBase21_mature_hsa) | -1.034 | 0.945 | 0.16805409 | 0.119745344 |
| hsa-miR-3135a (miRBase21_mature_hsa) | -1.034 | 0.945 | 0.1929062 | 0.14459747 |
| hsa-miR-3154 (miRBase21_mature_hsa) | -1.034 | 0.945 | 0.15794946 | 0.10964072 |
| hsa-miR-376a-3p (miRBase21_mature_hsa) | -1.034 | 0.945 | 0.267098 | 0.21878928 |
| hsa-miR-424-5p (miRBase21_mature_hsa) | -1.034 | 0.945 | 0.13608587 | 0.08777713 |
| hsa-miR-4638-5p (miRBase21_mature_hsa) | -1.034 | 0.945 | 0.267098 | 0.21878928 |
| hsa-miR-4644 (miRBase21_mature_hsa) | -1.034 | 0.945 | -0.005479002 | -0.05378775 |
| hsa-miR-4727-3p (miRBase21_mature_hsa) | -1.034 | 0.945 | 0.267098 | 0.21878928 |
| hsa-miR-627-5p (miRBase21_mature_hsa) | -1.034 | 0.945 | 0.16736262 | 0.11905389 |
| hsa-miR-1288-3p (miRBase21_mature_hsa) | -1.034 | 0.945 | -0.15282717 | -0.20113592 |
| hsa-miR-6500-3p (miRBase21_mature_hsa) | -1.034 | 0.945 | 0.24916162 | 0.20085286 |
| hsa-miR-6892-5p (miRBase21_mature_hsa) | -1.034 | 0.945 | 0.13517648 | 0.08686774 |
| hsa-miR-1193 (miRBase21_mature_hsa) | -1.034 | 0.945 | 0.2709008 | 0.22259204 |
| hsa-miR-3614-3p (miRBase21_mature_hsa) | -1.034 | 0.945 | 0.2709008 | 0.22259204 |
| hsa-miR-4710 (miRBase21_mature_hsa) | -1.034 | 0.945 | 0.13934836 | 0.09103962 |
| hsa-miR-552-3p (miRBase21_mature_hsa) | -1.034 | 0.945 | 0.059983023 | 0.011674278 |
| hsa-miR-6509-5p (miRBase21_mature_hsa) | -1.034 | 0.945 | 0.2709008 | 0.22259204 |
| hsa-miR-6827-5p (miRBase21_mature_hsa) | -1.034 | 0.945 | 0.2709008 | 0.22259204 |
| hsa-miR-6894-5p (miRBase21_mature_hsa) | -1.034 | 0.945 | 0.18349135 | 0.13518262 |
| hsa-miR-7107-3p (miRBase21_mature_hsa) | -1.034 | 0.945 | 0.1569156 | 0.10860687 |
| hsa-miR-3187-3p (miRBase21_mature_hsa) | -1.034 | 0.945 | 0.16714804 | 0.118839316 |
| hsa-miR-4734 (miRBase21_mature_hsa) | -1.034 | 0.945 | 0.17318723 | 0.12487848 |
| hsa-miR-4753-5p (miRBase21_mature_hsa) | -1.034 | 0.945 | 0.14160444 | 0.09329569 |
| hsa-miR-4804-5p (miRBase21_mature_hsa) | -1.034 | 0.945 | 0.16334361 | 0.11503487 |
| hsa-miR-522-5p (miRBase21_mature_hsa) | -1.034 | 0.945 | 0.263079 | 0.21477026 |
| hsa-miR-6889-3p (miRBase21_mature_hsa) | -1.034 | 0.945 | 0.263079 | 0.21477026 |
| hsa-miR-377-5p (miRBase21_mature_hsa) | -1.034 | 0.945 | 0.14782652 | 0.099517785 |
| hsa-miR-6873-3p (miRBase21_mature_hsa) | -1.034 | 0.945 | -0.5265224 | -0.5748312 |
| hsa-miR-3127-5p (miRBase21_mature_hsa) | -1.034 | 0.945 | -0.34513953 | -0.39344826 |
| hsa-miR-4645-3p (miRBase21_mature_hsa) | -1.034 | 0.945 | 0.1562522 | 0.10794347 |
| hsa-miR-642a-5p (miRBase21_mature_hsa) | -1.034 | 0.945 | 0.16760632 | 0.11929758 |
| hsa-let-7c-3p (miRBase21_mature_hsa) | -1.034 | 0.945 | 0.23744541 | 0.18913668 |
| hsa-miR-122-3p (miRBase21_mature_hsa) | -1.034 | 0.945 | 0.2646615 | 0.21635276 |
| hsa-miR-1224-5p (miRBase21_mature_hsa) | -1.034 | 0.945 | 0.039506167 | -0.008802573 |
| hsa-miR-1228-3p (miRBase21_mature_hsa) | -1.034 | 0.945 | 0.23798567 | 0.18967694 |
| hsa-miR-1256 (miRBase21_mature_hsa) | -1.034 | 0.945 | 0.2699539 | 0.22164518 |
| hsa-miR-1260a (miRBase21_mature_hsa) | -1.034 | 0.945 | 0.1711075 | 0.12279877 |
| hsa-miR-1269b (miRBase21_mature_hsa) | -1.034 | 0.945 | 0.294806 | 0.24649724 |
| hsa-miR-1306-3p (miRBase21_mature_hsa) | -1.034 | 0.945 | 0.24116203 | 0.1928533 |
| hsa-miR-1827 (miRBase21_mature_hsa) | -1.034 | 0.945 | 0.3689978 | 0.32068905 |
| hsa-miR-187-3p (miRBase21_mature_hsa) | -1.034 | 0.945 | 0.269711 | 0.22140224 |
| hsa-miR-190b (miRBase21_mature_hsa) | -1.034 | 0.945 | 0.1746086 | 0.12629984 |
| hsa-miR-193b-3p (miRBase21_mature_hsa) | -1.034 | 0.945 | 0.2699539 | 0.22164518 |
| hsa-miR-19b-1-5p (miRBase21_mature_hsa) | -1.034 | 0.945 | 0.2634642 | 0.21515548 |
| hsa-miR-202-3p (miRBase21_mature_hsa) | -1.034 | 0.945 | 0.2692624 | 0.22095367 |
| hsa-miR-202-5p (miRBase21_mature_hsa) | -1.034 | 0.945 | 0.2692624 | 0.22095367 |
| hsa-miR-210-5p (miRBase21_mature_hsa) | -1.034 | 0.945 | 0.2692624 | 0.22095367 |
| hsa-miR-216b-5p (miRBase21_mature_hsa) | -1.034 | 0.945 | 0.16442034 | 0.1161116 |
| hsa-miR-217 (miRBase21_mature_hsa) | -1.034 | 0.945 | 0.294806 | 0.24649724 |
| hsa-miR-219a-1-3p (miRBase21_mature_hsa) | -1.034 | 0.945 | 0.092004634 | 0.043695893 |
| hsa-miR-2276-3p (miRBase21_mature_hsa) | -1.034 | 0.945 | 0.26529095 | 0.21698222 |
| hsa-miR-2467-5p (miRBase21_mature_hsa) | -1.034 | 0.945 | 0.2536058 | 0.20529708 |
| hsa-miR-296-3p (miRBase21_mature_hsa) | -1.034 | 0.945 | 0.2699539 | 0.22164518 |
| hsa-miR-29c-3p (miRBase21_mature_hsa) | -1.034 | 0.945 | 0.25668055 | 0.20837182 |
| hsa-miR-301b-3p (miRBase21_mature_hsa) | -1.034 | 0.945 | 0.001439583 | -0.046869166 |
| hsa-miR-301b-5p (miRBase21_mature_hsa) | -1.034 | 0.945 | 0.24116203 | 0.1928533 |
| hsa-miR-3074-3p (miRBase21_mature_hsa) | -1.034 | 0.945 | 0.269711 | 0.22140224 |
| hsa-miR-3138 (miRBase21_mature_hsa) | -1.034 | 0.945 | 0.294806 | 0.24649724 |
| hsa-miR-3140-3p (miRBase21_mature_hsa) | -1.034 | 0.945 | 0.1950706 | 0.14676186 |
| hsa-miR-3143 (miRBase21_mature_hsa) | -1.034 | 0.945 | 0.2646615 | 0.21635276 |
| hsa-miR-3144-5p (miRBase21_mature_hsa) | -1.034 | 0.945 | 0.294806 | 0.24649724 |
| hsa-miR-3146 (miRBase21_mature_hsa) | -1.034 | 0.945 | 0.27910602 | 0.23079729 |

| | | | | |
|---|---|---|---|---|
| hsa-miR-3149 (miRBase21_mature_hsa) | -1.034 | 0.945 | 0.294806 | 0.24649724 |
| hsa-miR-3157-5p (miRBase21_mature_hsa) | -1.034 | 0.945 | 0.2646615 | 0.21635276 |
| hsa-miR-3159 (miRBase21_mature_hsa) | -1.034 | 0.945 | 0.26450035 | 0.21619162 |
| hsa-miR-3179 (miRBase21_mature_hsa) | -1.034 | 0.945 | 0.294806 | 0.24649724 |
| hsa-miR-3180 (miRBase21_mature_hsa) | -1.034 | 0.945 | 0.3689978 | 0.32068905 |
| hsa-miR-3180-3p (miRBase21_mature_hsa) | -1.034 | 0.945 | 0.027414411 | -0.020894332 |
| hsa-miR-3198 (miRBase21_mature_hsa) | -1.034 | 0.945 | -0.1566708 | -0.20497954 |
| hsa-miR-3678-5p (miRBase21_mature_hsa) | -1.034 | 0.945 | 0.27841967 | 0.23011094 |
| hsa-miR-3682-3p (miRBase21_mature_hsa) | -1.034 | 0.945 | 0.16624701 | 0.11793827 |
| hsa-miR-378a-5p (miRBase21_mature_hsa) | -1.034 | 0.945 | 0.2550126 | 0.20670387 |
| hsa-miR-378e (miRBase21_mature_hsa) | -1.034 | 0.945 | 0.3689978 | 0.32068905 |
| hsa-miR-378g (miRBase21_mature_hsa) | -1.034 | 0.945 | 0.294806 | 0.24649724 |
| hsa-miR-380-5p (miRBase21_mature_hsa) | -1.034 | 0.945 | 0.27841967 | 0.23011094 |
| hsa-miR-383-5p (miRBase21_mature_hsa) | -1.034 | 0.945 | 0.24116203 | 0.1928533 |
| hsa-miR-3912-3p (miRBase21_mature_hsa) | -1.034 | 0.945 | 0.294806 | 0.24649724 |
| hsa-miR-3918 (miRBase21_mature_hsa) | -1.034 | 0.945 | 0.294806 | 0.24649724 |
| hsa-miR-3939 (miRBase21_mature_hsa) | -1.034 | 0.945 | 0.269711 | 0.22140224 |
| hsa-miR-3940-3p (miRBase21_mature_hsa) | -1.034 | 0.945 | 0.294806 | 0.24649724 |
| hsa-miR-422a (miRBase21_mature_hsa) | -1.034 | 0.945 | 0.294806 | 0.24649724 |
| hsa-miR-4485-3p (miRBase21_mature_hsa) | -1.034 | 0.945 | 0.24874657 | 0.20043786 |
| hsa-miR-4488 (miRBase21_mature_hsa) | -1.034 | 0.945 | 0.1505763 | 0.102267556 |
| hsa-miR-4489 (miRBase21_mature_hsa) | -1.034 | 0.945 | 0.0374775 | -0.010831245 |
| hsa-miR-450a-2-3p (miRBase21_mature_hsa) | -1.034 | 0.945 | 0.24355182 | 0.19524308 |
| hsa-miR-4533 (miRBase21_mature_hsa) | -1.034 | 0.945 | 0.27910602 | 0.23079729 |
| hsa-miR-4635 (miRBase21_mature_hsa) | -1.034 | 0.945 | 0.2692624 | 0.22095367 |
| hsa-miR-4646-5p (miRBase21_mature_hsa) | -1.034 | 0.945 | 0.17913283 | 0.13082409 |
| hsa-miR-4648 (miRBase21_mature_hsa) | -1.034 | 0.945 | 0.2692624 | 0.22095367 |
| hsa-miR-4657 (miRBase21_mature_hsa) | -1.034 | 0.945 | 0.17402296 | 0.12571423 |
| hsa-miR-4658 (miRBase21_mature_hsa) | -1.034 | 0.945 | 0.2550126 | 0.20670387 |
| hsa-miR-4662a-5p (miRBase21_mature_hsa) | -1.034 | 0.945 | 0.05305762 | 0.004748881 |
| hsa-miR-4697-3p (miRBase21_mature_hsa) | -1.034 | 0.945 | 0.27841967 | 0.23011094 |
| hsa-miR-4708-3p (miRBase21_mature_hsa) | -1.034 | 0.945 | 0.294806 | 0.24649724 |
| hsa-miR-4721 (miRBase21_mature_hsa) | -1.034 | 0.945 | 0.2550126 | 0.20670387 |
| hsa-miR-4722-5p (miRBase21_mature_hsa) | -1.034 | 0.945 | 0.2815884 | 0.23327968 |
| hsa-miR-4775 (miRBase21_mature_hsa) | -1.034 | 0.945 | 0.294806 | 0.24649724 |
| hsa-miR-4796-3p (miRBase21_mature_hsa) | -1.034 | 0.945 | 0.25984928 | 0.21154055 |
| hsa-miR-4800-5p (miRBase21_mature_hsa) | -1.034 | 0.945 | 0.2692624 | 0.22095367 |
| hsa-miR-483-3p (miRBase21_mature_hsa) | -1.034 | 0.945 | -0.128678 | -0.17698672 |
| hsa-miR-5001-3p (miRBase21_mature_hsa) | -1.034 | 0.945 | 0.264173 | 0.21586426 |
| hsa-miR-5001-5p (miRBase21_mature_hsa) | -1.034 | 0.945 | 0.18317509 | 0.13486634 |
| hsa-miR-5004-5p (miRBase21_mature_hsa) | -1.034 | 0.945 | 0.2550126 | 0.20670387 |
| hsa-miR-5006-3p (miRBase21_mature_hsa) | -1.034 | 0.945 | 0.2536058 | 0.20529708 |
| hsa-miR-500a-5p (miRBase21_mature_hsa) | -1.034 | 0.945 | 0.294806 | 0.24649724 |
| hsa-miR-504-3p (miRBase21_mature_hsa) | -1.034 | 0.945 | 0.2318667 | 0.18355796 |
| hsa-miR-505-3p (miRBase21_mature_hsa) | -1.034 | 0.945 | 0.045366377 | -0.002942366 |
| hsa-miR-508-3p (miRBase21_mature_hsa) | -1.034 | 0.945 | 0.26529095 | 0.21698222 |
| hsa-miR-514a-3p (miRBase21_mature_hsa) | -1.034 | 0.945 | 0.27664107 | 0.22833234 |
| hsa-miR-517a-3p (miRBase21_mature_hsa) | -1.034 | 0.945 | 0.2550126 | 0.20670387 |
| hsa-miR-517b-3p (miRBase21_mature_hsa) | -1.034 | 0.945 | 0.3689978 | 0.32068905 |
| hsa-miR-518e-5p (miRBase21_mature_hsa) | -1.034 | 0.945 | 0.3689978 | 0.32068905 |
| hsa-miR-5190 (miRBase21_mature_hsa) | -1.034 | 0.945 | 0.26529095 | 0.21698222 |
| hsa-miR-519a-5p (miRBase21_mature_hsa) | -1.034 | 0.945 | 0.3689978 | 0.32068905 |
| hsa-miR-519b-5p (miRBase21_mature_hsa) | -1.034 | 0.945 | 0.3689978 | 0.32068905 |
| hsa-miR-519c-5p (miRBase21_mature_hsa) | -1.034 | 0.945 | 0.3689978 | 0.32068905 |
| hsa-miR-523-5p (miRBase21_mature_hsa) | -1.034 | 0.945 | 0.3689978 | 0.32068905 |
| hsa-miR-539-3p (miRBase21_mature_hsa) | -1.034 | 0.945 | 0.24116203 | 0.1928533 |
| hsa-miR-548aa (miRBase21_mature_hsa) | -1.034 | 0.945 | 0.3689978 | 0.32068905 |
| hsa-miR-548ad-5p (miRBase21_mature_hsa) | -1.034 | 0.945 | 0.3689978 | 0.32068905 |
| hsa-miR-548ae-5p (miRBase21_mature_hsa) | -1.034 | 0.945 | 0.26529095 | 0.21698222 |
| hsa-miR-548ag (miRBase21_mature_hsa) | -1.034 | 0.945 | 0.27910602 | 0.23079729 |
| hsa-miR-548ay-5p (miRBase21_mature_hsa) | -1.034 | 0.945 | 0.3689978 | 0.32068905 |
| hsa-miR-548g-3p (miRBase21_mature_hsa) | -1.034 | 0.945 | 0.19615616 | 0.14784741 |
| hsa-miR-548n (miRBase21_mature_hsa) | -1.034 | 0.945 | 0.27664107 | 0.22833234 |
| hsa-miR-550b-2-5p (miRBase21_mature_hsa) | -1.034 | 0.945 | 0.27664107 | 0.22833234 |
| hsa-miR-551b-5p (miRBase21_mature_hsa) | -1.034 | 0.945 | 0.2536058 | 0.20529708 |
| hsa-miR-5589-5p (miRBase21_mature_hsa) | -1.034 | 0.945 | 0.26450035 | 0.21619162 |
| hsa-miR-6087 (miRBase21_mature_hsa) | -1.034 | 0.945 | 0.13562848 | 0.087319724 |
| hsa-miR-6130 (miRBase21_mature_hsa) | -1.034 | 0.945 | 0.3689978 | 0.32068905 |
| hsa-miR-619-5p (miRBase21_mature_hsa) | -1.034 | 0.945 | 0.2536058 | 0.20529708 |
| hsa-miR-629-3p (miRBase21_mature_hsa) | -1.034 | 0.945 | 0.14187714 | 0.0935684 |
| hsa-miR-6502-5p (miRBase21_mature_hsa) | -1.034 | 0.945 | 0.2536058 | 0.20529708 |

| | | | | |
|---|---|---|---|---|
| hsa-miR-6510-3p (miRBase21_mature_hsa) | -1.034 | 0.945 | 0.2699539 | 0.22164518 |
| hsa-miR-6716-3p (miRBase21_mature_hsa) | -1.034 | 0.945 | 0.2815884 | 0.23327968 |
| hsa-miR-6726-5p (miRBase21_mature_hsa) | -1.034 | 0.945 | 0.2536058 | 0.20529708 |
| hsa-miR-6737-3p (miRBase21_mature_hsa) | -1.034 | 0.945 | 0.16624701 | 0.11793827 |
| hsa-miR-6747-5p (miRBase21_mature_hsa) | -1.034 | 0.945 | 0.23798567 | 0.18967694 |
| hsa-miR-6755-5p (miRBase21_mature_hsa) | -1.034 | 0.945 | 0.2646615 | 0.21635276 |
| hsa-miR-6757-5p (miRBase21_mature_hsa) | -1.034 | 0.945 | 0.15325397 | 0.104945235 |
| hsa-miR-676-3p (miRBase21_mature_hsa) | -1.034 | 0.945 | 0.27664107 | 0.22833234 |
| hsa-miR-6772-5p (miRBase21_mature_hsa) | -1.034 | 0.945 | 0.07206358 | 0.023754835 |
| hsa-miR-6777-5p (miRBase21_mature_hsa) | -1.034 | 0.945 | 0.22700745 | 0.17869873 |
| hsa-miR-6783-3p (miRBase21_mature_hsa) | -1.034 | 0.945 | 0.24752328 | 0.19921455 |
| hsa-miR-6784-3p (miRBase21_mature_hsa) | -1.034 | 0.945 | 0.2550126 | 0.20670387 |
| hsa-miR-6796-5p (miRBase21_mature_hsa) | -1.034 | 0.945 | 0.2699539 | 0.22164518 |
| hsa-miR-6802-5p (miRBase21_mature_hsa) | -1.034 | 0.945 | 0.17378005 | 0.12547131 |
| hsa-miR-6804-3p (miRBase21_mature_hsa) | -1.034 | 0.945 | 0.27664107 | 0.22833234 |
| hsa-miR-6810-3p (miRBase21_mature_hsa) | -1.034 | 0.945 | 0.092001304 | 0.043692563 |
| hsa-miR-6810-5p (miRBase21_mature_hsa) | -1.034 | 0.945 | 0.17709097 | 0.12878224 |
| hsa-miR-6812-3p (miRBase21_mature_hsa) | -1.034 | 0.945 | 0.01415397 | -0.03415478 |
| hsa-miR-6815-5p (miRBase21_mature_hsa) | -1.034 | 0.945 | 0.27841967 | 0.23011094 |
| hsa-miR-6825-5p (miRBase21_mature_hsa) | -1.034 | 0.945 | 0.2699539 | 0.22164518 |
| hsa-miR-6853-3p (miRBase21_mature_hsa) | -1.034 | 0.945 | 0.17539915 | 0.12709042 |
| hsa-miR-6867-5p (miRBase21_mature_hsa) | -1.034 | 0.945 | 0.294806 | 0.24649724 |
| hsa-miR-6869-5p (miRBase21_mature_hsa) | -1.034 | 0.945 | 0.23744541 | 0.18913668 |
| hsa-miR-6876-5p (miRBase21_mature_hsa) | -1.034 | 0.945 | 0.27910602 | 0.23079729 |
| hsa-miR-6878-5p (miRBase21_mature_hsa) | -1.034 | 0.945 | 0.27664107 | 0.22833234 |
| hsa-miR-6882-3p (miRBase21_mature_hsa) | -1.034 | 0.945 | 0.24874657 | 0.20043786 |
| hsa-miR-6884-5p (miRBase21_mature_hsa) | -1.034 | 0.945 | 0.13213131 | 0.08382258 |
| hsa-miR-6891-5p (miRBase21_mature_hsa) | -1.034 | 0.945 | 0.24116203 | 0.1928533 |
| hsa-miR-7110-5p (miRBase21_mature_hsa) | -1.034 | 0.945 | 0.27910602 | 0.23079729 |
| hsa-miR-7151-5p (miRBase21_mature_hsa) | -1.034 | 0.945 | 0.2699539 | 0.22164518 |
| hsa-miR-7641 (miRBase21_mature_hsa) | -1.034 | 0.945 | 0.16265592 | 0.11434718 |
| hsa-miR-7855-5p (miRBase21_mature_hsa) | -1.034 | 0.945 | 0.27841967 | 0.23011094 |
| hsa-miR-7856-5p (miRBase21_mature_hsa) | -1.034 | 0.945 | 0.23744541 | 0.18913668 |
| hsa-miR-7976 (miRBase21_mature_hsa) | -1.034 | 0.945 | 0.10367424 | 0.0553655 |
| hsa-miR-7978 (miRBase21_mature_hsa) | -1.034 | 0.945 | 0.2550126 | 0.20670387 |
| hsa-miR-3115 (miRBase21_mature_hsa) | -1.034 | 0.945 | 0.05497182 | 0.006663076 |
| hsa-miR-324-5p (miRBase21_mature_hsa) | -1.034 | 0.945 | 0.20528111 | 0.15697238 |
| hsa-miR-154-5p (miRBase21_mature_hsa) | -1.034 | 0.945 | 0.20234883 | 0.15404008 |
| hsa-miR-212-5p (miRBase21_mature_hsa) | -1.034 | 0.945 | -0.090980075 | -0.13928881 |
| hsa-miR-6894-3p (miRBase21_mature_hsa) | -1.034 | 0.945 | -0.08913412 | -0.13744286 |
| hsa-miR-127-5p (miRBase21_mature_hsa) | -1.034 | 0.945 | 0.19854607 | 0.15023732 |
| hsa-miR-6789-3p (miRBase21_mature_hsa) | -1.034 | 0.945 | 0.08584453 | 0.037535783 |
| hsa-miR-365b-5p (miRBase21_mature_hsa) | -1.034 | 0.945 | -0.17234245 | -0.22065118 |
| hsa-miR-532-3p (miRBase21_mature_hsa) | -1.034 | 0.945 | -0.03916334 | -0.08747208 |
| hsa-miR-429 (miRBase21_mature_hsa) | -1.034 | 0.945 | 0.023546994 | -0.024761736 |
| hsa-miR-3188 (miRBase21_mature_hsa) | 1.095 | 0.948 | 1.1757294 | 1.3062173 |
| hsa-miR-6738-3p (miRBase21_mature_hsa) | -1.091 | 0.957 | 1.2360923 | 1.1101375 |
| hsa-miR-335-3p (miRBase21_mature_hsa) | -1.047 | 0.959 | 0.7149027 | 0.64914805 |
| hsa-miR-101-3p (miRBase21_mature_hsa) | -1.024 | 0.960 | 0.18511392 | 0.15100034 |
| hsa-miR-6852-5p (miRBase21_mature_hsa) | 1.032 | 0.965 | 1.3127717 | 1.3588023 |
| hsa-miR-338-5p (miRBase21_mature_hsa) | 1.066 | 0.966 | 0.30411392 | 0.39565253 |
| hsa-let-7e-3p (miRBase21_mature_hsa) | 1.041 | 0.967 | 0.71895015 | 0.7771089 |
| hsa-miR-2277-5p (miRBase21_mature_hsa) | 1.053 | 0.968 | 1.482951 | 1.5579404 |
| hsa-miR-432-5p (miRBase21_mature_hsa) | 1.027 | 0.968 | 0.565422 | 0.60385174 |
| hsa-miR-182-5p (miRBase21_mature_hsa) | -1.021 | 0.970 | 0.5170917 | 0.4865276 |
| hsa-miR-92b-3p (miRBase21_mature_hsa) | -1.025 | 0.978 | -0.43972877 | -0.47553813 |
| hsa-miR-218-5p (miRBase21_mature_hsa) | -1.025 | 0.979 | 0.23574543 | 0.20026138 |
| hsa-miR-7-1-3p (miRBase21_mature_hsa) | 1.025 | 0.980 | 0.7507986 | 0.7871074 |
| hsa-miR-5010-5p (miRBase21_mature_hsa) | 1.023 | 0.982 | 0.005458172 | 0.03891292 |
| hsa-miR-99b-5p (miRBase21_mature_hsa) | -1.011 | 0.984 | 0.2504641 | 0.23494744 |
| hsa-miR-3174 (miRBase21_mature_hsa) | -1.025 | 0.985 | 0.76529247 | 0.73026407 |
| hsa-miR-5004-3p (miRBase21_mature_hsa) | -1.018 | 0.986 | 0.40173525 | 0.37636188 |
| hsa-miR-3679-5p (miRBase21_mature_hsa) | -1.015 | 0.989 | 0.4848979 | 0.46349648 |
| hsa-miR-664a-3p (miRBase21_mature_hsa) | 1.015 | 0.991 | 0.48881128 | 0.5098208 |
| hsa-miR-502-3p (miRBase21_mature_hsa) | -1.008 | 0.992 | 0.40592363 | 0.3947446 |
| hsa-miR-433-3p (miRBase21_mature_hsa) | -1.005 | 0.996 | 0.8174098 | 0.8096725 |
| hsa-miR-30e-5p (miRBase21_mature_hsa) | -1.004 | 0.998 | -0.6168241 | -0.6227832 |
| hsa-miR-148a-3p (miRBase21_mature_hsa) | 1.002 | 0.998 | 0.48403314 | 0.48643073 |
| hsa-miR-1246 (miRBase21_mature_hsa) | 1.002 | 0.999 | -0.24141394 | -0.23882394 |

**Data Set for Fig 3.**

| Sample Name | Detector Name | -2ΔΔCT | Detector Name | -2ΔΔCT | Detector Name | -2ΔΔCT |
|---|---|---|---|---|---|---|
| CTL1 | has-miR-20b-5p | 1.157221207 | hsa-miR-204-5p | 0.000158855 | has-miR-330-3p | 0.509870226 |
| CTL2 | has-miR-20b-5p | 1.239218282 | hsa-miR-204-5p | 0.000177487 | has-miR-330-3p | 1.149475726 |
| CTL3 | has-miR-20b-5p | 1.939767399 | hsa-miR-204-5p | 0.000034334 | has-miR-330-3p | 3.947693547 |
| CTL4 | has-miR-20b-5p | 0.962737906 | hsa-miR-204-5p | 0.000050970 | has-miR-330-3p | 0.833968039 |
| CTL5 | has-miR-20b-5p | 2.843803273 | hsa-miR-204-5p | 0.000186311 | has-miR-330-3p | 3.133328433 |
| CTL6 | has-miR-20b-5p | 5.529955994 | hsa-miR-204-5p | 0.000372622 | has-miR-330-3p | 3.597473351 |
| AF1 | has-miR-20b-5p | 0.557605231 | hsa-miR-204-5p | 0.000015153 | has-miR-330-3p | 0.194777508 |
| AF2 | has-miR-20b-5p | 1.124191647 | hsa-miR-204-5p | 0.000046256 | has-miR-330-3p | 0.269208152 |
| AF3 | has-miR-20b-5p | 0.239783839 | hsa-miR-204-5p | 0.000040268 | has-miR-330-3p | 0.371642080 |
| AF4 | has-miR-20b-5p | 0.201684390 | hsa-miR-204-5p | 0.000020274 | has-miR-330-3p | 0.069722881 |
| AF5 | has-miR-20b-5p | 0.267503684 | hsa-miR-204-5p | 0.000045619 | has-miR-330-3p | 0.146492509 |
| AF6 | has-miR-20b-5p | 0.236563325 | hsa-miR-204-5p | 0.000014139 | has-miR-330-3p | 0.165704881 |

**Data Set for Table 4, Fig 4, Fig 5.**

| Pt No. | AF Recurence | Age, y/o | Gender | AF Type | BMI | CHADS2 score, pts | CHA2DS2 VASc score, pts | CHF | HT | DM | Stroke TIA | Vascular disease | β-blocker | ACEI/ARB | Diuretics | AAD | Cr, mg/dl | CrCl, ml/min | NTProBNP, pg/ml | LVDd, mm | LVDs, mm | EF, % | LAD, mm | miRNA | -2ΔΔCT | miRNA | -2ΔΔCT | miRNA | -2ΔΔCT |
|---|---|---|---|---|---|---|---|---|---|---|---|---|---|---|---|---|---|---|---|---|---|---|---|---|---|---|---|---|---|
| 1 | Yes | 66 | Female | PAF | 26.8 | 2 | 3 | 0 | 1 | 0 | 0 | 0 | 1 | 0 | 0 | 0 | 0.58 | 85 | 325 | 48 | 37 | 60 | 41 | has-miR-20b-5p | 0.052096208 | hsa-miR-204-5p | 0.018473891 | has-miR-330-3p | 0.008341003 |
| 2 | No | 30 | Male | PAF | 24.5 | 0 | 0 | 0 | 0 | 0 | 0 | 0 | 0 | 0 | 0 | 1 | 0.79 | 143 | 14 | 44 | 33 | 61 | 32 | has-miR-20b-5p | 0.024386057 | hsa-miR-204-5p | 0.010238726 | has-miR-330-3p | 0.021786748 |
| 3 | No | 75 | Female | PAF | 15.5 | 2 | 4 | 0 | 1 | 0 | 0 | 0 | 1 | 0 | 0 | 0 | 0.72 | 47 | 213 | 36 | 27 | 64 | 25 | has-miR-20b-5p | 0.031733790 | hsa-miR-204-5p | 0.033321335 | has-miR-330-3p | 0.023067743 |
| 4 | Yes | 79 | Male | PeAF | 27.2 | 2 | 3 | 0 | 1 | 0 | 0 | 0 | 1 | 1 | 0 | 1 | 1.00 | 64 | 324 | 50 | 35 | 60 | 40 | has-miR-20b-5p | 0.029029462 | hsa-miR-204-5p | 0.061949051 | has-miR-330-3p | 0.016088694 |
| 5 | No | 65 | Male | PAF | 19.7 | 2 | 3 | 1 | 0 | 1 | 0 | 0 | 0 | 0 | 0 | 0 | 0.98 | 61 | 351 | 50 | 35 | 56 | 31 | has-miR-20b-5p | 0.118207679 | hsa-miR-204-5p | 0.013211831 | has-miR-330-3p | 0.028202327 |
| 6 | No | 73 | Male | PAF | 25.5 | 4 | 5 | 0 | 1 | 1 | 1 | 0 | 0 | 1 | 0 | 0 | 0.89 | 75 | 191 | 49 | 33 | 60 | 42 | has-miR-20b-5p | 0.026386955 | hsa-miR-204-5p | 0.027426931 | has-miR-330-3p | 0.003077600 |
| 7 | No | 75 | Male | PeAF | 24.5 | 3 | 4 | 1 | 1 | 0 | 0 | 1 | 1 | 0 | 0 | 0 | 0.92 | 64 | 523 | 48 | 27 | 63 | 41 | has-miR-20b-5p | 0.070066096 | hsa-miR-204-5p | 0.010282888 | has-miR-330-3p | 0.006691897 |
| 8 | Yes | 61 | Female | PAF | 19.2 | 1 | 2 | 0 | 0 | 1 | 0 | 0 | 0 | 0 | 0 | 0 | 0.79 | 55 | 290 | 43 | 29 | 61 | 39 | has-miR-20b-5p | 0.015737185 | hsa-miR-204-5p | 0.022473630 | has-miR-330-3p | 0.014810036 |
| 9 | No | 57 | Male | PAF | 19.1 | 0 | 0 | 0 | 0 | 0 | 0 | 0 | 0 | 0 | 0 | 0 | 0.79 | 98 | 10 | 44 | 31 | 61 | 32 | has-miR-20b-5p | 0.021383606 | hsa-miR-204-5p | 0.013219615 | has-miR-330-3p | 0.005586039 |
| 10 | No | 73 | Female | PeAF | 23.5 | 2 | 4 | 0 | 1 | 1 | 0 | 0 | 1 | 1 | 0 | 0 | 0.71 | 61 | 1074 | 35 | 25 | 57 | 41 | has-miR-20b-5p | 0.019119337 | hsa-miR-204-5p | 0.006747945 | has-miR-330-3p | 0.004637437 |
| 11 | No | 71 | Male | PAF | 17.3 | 1 | 2 | 0 | 0 | 1 | 0 | 0 | 0 | 0 | 0 | 1 | 0.76 | 67 | 77 | 37 | 26 | 59 | 55 | has-miR-20b-5p | 0.006308437 | hsa-miR-204-5p | 0.019143200 | has-miR-330-3p | 0.003432543 |
| 12 | No | 68 | Male | PeAF | 24.9 | 2 | 3 | 1 | 1 | 0 | 0 | 0 | 1 | 1 | 0 | 0 | 0.93 | 77 | 830 | 48 | 34 | 51 | 48 | has-miR-20b-5p | 0.024074072 | hsa-miR-204-5p | 0.009137821 | has-miR-330-3p | 0.001126016 |
| 13 | No | 75 | Male | PAF | 29.9 | 1 | 3 | 0 | 1 | 0 | 0 | 0 | 0 | 0 | 0 | 1 | 0.81 | 86 | 162 | 54 | 38 | 55 | 53 | has-miR-20b-5p | 0.000602523 | hsa-miR-204-5p | 0.011002660 | has-miR-330-3p | 0.001305290 |
| 14 | No | 77 | Female | PAF | 22.3 | 3 | 5 | 0 | 1 | 1 | 0 | 0 | 0 | 1 | 1 | 0 | 0.69 | 57 | 2125 | 35 | 23 | 65 | 29 | has-miR-20b-5p | 0.034389072 | hsa-miR-204-5p | 0.007989351 | has-miR-330-3p | 0.003633845 |
| 15 | No | 81 | Male | PAF | 24.3 | 3 | 4 | 1 | 1 | 0 | 0 | 0 | 1 | 1 | 1 | 0 | 0.99 | 58 | 861 | 46 | 31 | 47 | 34 | has-miR-20b-5p | 0.041012050 | hsa-miR-204-5p | 0.009117820 | has-miR-330-3p | 0.010065684 |
| 16 | No | 54 | Male | PAF | 27.4 | 1 | 1 | 0 | 0 | 1 | 0 | 0 | 0 | 0 | 0 | 0 | 1.02 | 85 | 525 | 39 | 29 | 66 | 40 | has-miR-20b-5p | 0.114551189 | hsa-miR-204-5p | 0.012157724 | has-miR-330-3p | 0.016101120 |
| 17 | No | 82 | Female | PAF | 21.6 | 3 | 5 | 1 | 1 | 0 | 0 | 0 | 1 | 1 | 0 | 0 | 0.66 | 48 | 535 | 44 | 28 | 64 | 37 | has-miR-20b-5p | 0.017494238 | hsa-miR-204-5p | 0.003127919 | has-miR-330-3p | 0.009562406 |
| 18 | No | 58 | Male | PeAF | 22.7 | 2 | 2 | 1 | 1 | 0 | 0 | 0 | 1 | 1 | 1 | 0 | 0.99 | 76 | 150 | 51 | 36 | 65 | 49 | has-miR-20b-5p | 0.057585764 | hsa-miR-204-5p | 0.018594323 | has-miR-330-3p | 0.008484923 |
| 19 | No | 70 | Male | PeAF | 23.6 | 1 | 2 | 0 | 1 | 0 | 0 | 0 | 0 | 0 | 0 | 0 | 1.10 | 64 | 614 | 45 | 29 | 58 | 39 | has-miR-20b-5p | 0.023711691 | hsa-miR-204-5p | 0.003378260 | has-miR-330-3p | 0.009663287 |
| 20 | Yes | 55 | Male | PAF | 23.3 | 1 | 1 | 1 | 0 | 0 | 0 | 0 | 0 | 0 | 0 | 0 | 0.77 | 106 | 146 | 46 | 31 | 67 | 37 | has-miR-20b-5p | 0.129145505 | hsa-miR-204-5p | 0.044160582 | has-miR-330-3p | 0.037703590 |
| 21 | Yes | 66 | Male | PeAF | 21.4 | 5 | 6 | 1 | 1 | 1 | 1 | 0 | 0 | 0 | 0 | 0 | 0.75 | 85 | 1011 | 51 | 41 | 48 | 47 | has-miR-20b-5p | 0.024221113 | hsa-miR-204-5p | 0.010637803 | has-miR-330-3p | 0.017070562 |
| 22 | No | 80 | Female | PeAF | 26.4 | 3 | 5 | 1 | 1 | 0 | 0 | 0 | 1 | 1 | 1 | 0 | 0.91 | 45 | 1158 | 51 | 33 | 56 | 42 | has-miR-20b-5p | 0.023973339 | hsa-miR-204-5p | 0.003207685 | has-miR-330-3p | 0.006815297 |
| 23 | Yes | 40 | Male | PAF | 23.3 | 0 | 0 | 0 | 0 | 0 | 0 | 0 | 0 | 0 | 0 | 0 | 0.89 | 107 | 40 | 55 | 35 | 60 | 41 | has-miR-20b-5p | 0.051234497 | hsa-miR-204-5p | 0.002861896 | has-miR-330-3p | 0.007512991 |
| 24 | No | 67 | Female | PAF | 24.5 | 1 | 3 | 0 | 1 | 0 | 0 | 0 | 0 | 1 | 0 | 0 | 0.54 | 99 | 70 | 49 | 30 | 55 | 42 | has-miR-20b-5p | 0.049113110 | hsa-miR-204-5p | 0.015162619 | has-miR-330-3p | 0.007609814 |
| 25 | Yes | 71 | Male | PAF | 27.4 | 2 | 3 | 0 | 0 | 0 | 1 | 0 | 0 | 0 | 0 | 0 | 0.90 | 80 | 42 | 48 | 30 | 61 | 49 | has-miR-20b-5p | 0.011353692 | hsa-miR-204-5p | 0.000609196 | has-miR-330-3p | 0.003302114 |
| 26 | No | 73 | Male | PAF | 20.5 | 2 | 3 | 1 | 1 | 0 | 0 | 0 | 0 | 0 | 0 | 0 | 1.01 | 49 | 73 | 45 | 41 | 30 | 39 | has-miR-20b-5p | 0.092381343 | hsa-miR-204-5p | 0.002692265 | has-miR-330-3p | 0.008812357 |
| 27 | Yes | 50 | Male | PAF | 24.8 | 0 | 0 | 0 | 0 | 0 | 0 | 0 | 1 | 0 | 0 | 1 | 0.82 | 113 | 134 | 46 | 27 | 57 | 31 | has-miR-20b-5p | 0.021993246 | hsa-miR-204-5p | 0.025936298 | has-miR-330-3p | 0.006467858 |
| 28 | No | 60 | Female | PeAF | 29.2 | 1 | 2 | 0 | 1 | 0 | 0 | 0 | 1 | 1 | 0 | 0 | 0.84 | 85 | 365 | 51 | 43 | 54 | 37 | has-miR-20b-5p | 0.033663376 | hsa-miR-204-5p | 0.003512386 | has-miR-330-3p | 0.015250133 |
| 29 | No | 74 | Female | PAF | 22.9 | 0 | 1 | 0 | 0 | 0 | 0 | 1 | 1 | 0 | 0 | 0 | 0.66 | 63 | 118 | 37 | 23 | 58 | 32 | has-miR-20b-5p | 0.039050450 | hsa-miR-204-5p | 0.004513202 | has-miR-330-3p | 0.030762040 |
| 30 | No | 74 | Male | PeAF | 25.2 | 2 | 3 | 1 | 1 | 0 | 0 | 0 | 1 | 1 | 1 | 0 | 0.92 | 63 | 544 | 41 | 28 | 61 | 42 | has-miR-20b-5p | 0.010894532 | hsa-miR-204-5p | 0.012098216 | has-miR-330-3p | 0.004498050 |
| 31 | No | 78 | Male | PeAF | 22.6 | 1 | 2 | 0 | 0 | 0 | 0 | 0 | 0 | 0 | 0 | 0 | 1.30 | 39 | 342 | 40 | 29 | 53 | 51 | has-miR-20b-5p | 0.067933163 | hsa-miR-204-5p | 0.010657028 | has-miR-330-3p | 0.028756316 |
| 32 | No | 74 | Male | PeAF | 27 | 1 | 2 | 0 | 0 | 1 | 0 | 0 | 1 | 0 | 0 | 0 | 0.87 | 84 | 419 | 53 | 39 | 53 | 51 | has-miR-20b-5p | 0.091603911 | hsa-miR-204-5p | 0.003989233 | has-miR-330-3p | 0.022690628 |
| 33 | No | 73 | Female | PeAF | 26.6 | 1 | 3 | 1 | 0 | 0 | 0 | 0 | 1 | 0 | 0 | 0 | 0.69 | 75 | 950 | 42 | 25 | 56 | 40 | has-miR-20b-5p | 0.026229177 | hsa-miR-204-5p | 0.013111059 | has-miR-330-3p | 0.009291417 |
| 34 | No | 69 | Male | PAF | 25.6 | 0 | 1 | 0 | 0 | 0 | 0 | 0 | 1 | 0 | 0 | 0 | 0.77 | 97 | 90 | 41 | 26 | 63 | 27 | has-miR-20b-5p | 0.109417823 | hsa-miR-204-5p | 0.017414948 | has-miR-330-3p | 0.039905443 |
| 35 | No | 76 | Male | PAF | 24.1 | 2 | 3 | 0 | 1 | 0 | 0 | 0 | 1 | 1 | 0 | 0 | 1.00 | 49 | 84 | 42 | 23 | 65 | 33 | has-miR-20b-5p | 0.024008305 | hsa-miR-204-5p | 0.010789314 | has-miR-330-3p | 0.006969348 |
| 36 | No | 75 | Female | PAF | 20.2 | 2 | 3 | 0 | 1 | 0 | 0 | 0 | 0 | 0 | 0 | 0 | 0.83 | 44 | 71 | 42 | 28 | 63 | 35 | has-miR-20b-5p | 0.024876943 | hsa-miR-204-5p | 0.007921196 | has-miR-330-3p | 0.011125968 |
| 37 | No | 73 | Male | PeAF | 18.6 | 2 | 3 | 0 | 1 | 1 | 0 | 0 | 1 | 0 | 0 | 0 | 0.74 | 58 | 463 | 41 | 27 | 57 | 29 | has-miR-20b-5p | 0.057004186 | hsa-miR-204-5p | 0.007998488 | has-miR-330-3p | 0.002357681 |
| 38 | No | 40 | Male | PAF | 31.1 | 3 | 3 | 0 | 0 | 1 | 1 | 0 | 1 | 0 | 0 | 0 | 1.06 | 111 | 182 | 52 | 36 | 55 | 42 | has-miR-20b-5p | 0.208319433 | hsa-miR-204-5p | 0.026072902 | has-miR-330-3p | 0.061363886 |
| 39 | No | 53 | Male | PeAF | 22.6 | 0 | 0 | 0 | 0 | 0 | 0 | 0 | 0 | 0 | 0 | 0 | 0.79 | 87 | 209 | 53 | 38 | 42 | 40 | has-miR-20b-5p | 0.090554867 | hsa-miR-204-5p | 0.005603826 | has-miR-330-3p | 0.018222996 |
| 40 | No | 49 | Male | PeAF | 22.9 | 0 | 0 | 0 | 0 | 0 | 0 | 0 | 0 | 0 | 0 | 0 | 0.78 | 121 | 645 | 58 | 47 | 32 | 39 | has-miR-20b-5p | 0.025143588 | hsa-miR-204-5p | 0.007203761 | has-miR-330-3p | 0.001944471 |
| 41 | No | 68 | Male | PAF | 21.9 | 1 | 2 | 0 | 1 | 0 | 0 | 0 | 0 | 0 | 0 | 0 | 0.82 | 73 | 55 | 44 | 33 | 63 | 38 | has-miR-20b-5p | 0.022884152 | hsa-miR-204-5p | 0.018707077 | has-miR-330-3p | 0.000356524 |
| 42 | No | 62 | Male | PeAF | 22.5 | 1 | 1 | 1 | 0 | 0 | 0 | 0 | 1 | 0 | 0 | 0 | 0.84 | 89 | 1671 | 53 | 36 | 50 | 44 | has-miR-20b-5p | 0.037388135 | hsa-miR-204-5p | 0.005547744 | has-miR-330-3p | 0.004865876 |
| 43 | No | 76 | Male | PAF | 24 | 2 | 3 | 0 | 0 | 1 | 0 | 0 | 0 | 0 | 0 | 0 | 0.80 | 75 | 219 | 40 | 38 | 52 | 42 | has-miR-20b-5p | 0.062220897 | hsa-miR-204-5p | 0.009567416 | has-miR-330-3p | 0.012588053 |
| 44 | No | 59 | Male | PAF | 27.4 | 1 | 1 | 0 | 1 | 0 | 0 | 0 | 1 | 1 | 0 | 0 | 1.06 | 86 | 180 | 50 | 30 | 65 | 39 | has-miR-20b-5p | 0.025142519 | hsa-miR-204-5p | 0.001754479 | has-miR-330-3p | 0.012370409 |
| 45 | No | 70 | Male | PAF | 20.9 | 1 | 2 | 0 | 1 | 0 | 0 | 0 | 0 | 1 | 0 | 0 | 0.80 | 60 | 110 | 42 | 27 | 62 | 34 | has-miR-20b-5p | 0.042428236 | hsa-miR-204-5p | 0.004908218 | has-miR-330-3p | 0.007105497 |
| 46 | Yes | 74 | Female | PAF | 26.8 | 0 | 2 | 0 | 0 | 0 | 0 | 0 | 0 | 0 | 0 | 0 | 0.70 | 72 | 75 | 50 | 38 | 56 | 48 | has-miR-20b-5p | 0.024750966 | hsa-miR-204-5p | 0.025940282 | has-miR-330-3p | 0.006426150 |
| 47 | No | 72 | Female | PAF | 23.3 | 1 | 3 | 0 | 1 | 0 | 0 | 0 | 0 | 1 | 0 | 0 | 0.65 | 68 | 282 | 40 | 25 | 59 | 41 | has-miR-20b-5p | 0.046086664 | hsa-miR-204-5p | 0.013782061 | has-miR-330-3p | 0.014855586 |
| 48 | No | 63 | Male | PeAF | 30.4 | 1 | 1 | 0 | 1 | 0 | 0 | 0 | 0 | 0 | 0 | 0 | 0.81 | 114 | 231 | 46 | 31 | 65 | 47 | has-miR-20b-5p | 0.046431032 | hsa-miR-204-5p | 0.014811997 | has-miR-330-3p | 0.016601334 |
| 49 | No | 41 | Male | PAF | 21.4 | 0 | 0 | 0 | 0 | 0 | 0 | 0 | 1 | 0 | 0 | 0 | 0.92 | 105 | 35 | 52 | 34 | 60 | 33 | has-miR-20b-5p | 0.116601857 | hsa-miR-204-5p | 0.007552961 | has-miR-330-3p | 0.032575896 |
| 50 | No | 59 | Female | PAF | 24.5 | 1 | 2 | 0 | 0 | 1 | 0 | 0 | 0 | 0 | 0 | 0 | 0.71 | 84 | 22 | 40 | 22 | 65 | 34 | has-miR-20b-5p | 0.148345938 | hsa-miR-204-5p | 0.006345599 | has-miR-330-3p | 0.032487165 |
| 51 | No | 76 | Female | PeAF | 23.1 | 2 | 4 | 0 | 1 | 0 | 0 | 0 | 1 | 1 | 0 | 0 | 0.64 | 64 | 1522 | 43 | 31 | 60 | 45 | has-miR-20b-5p | 0.047126227 | hsa-miR-204-5p | 0.004627831 | has-miR-330-3p | 0.005085601 |
| 52 | No | 54 | Male | PeAF | 24.2 | 1 | 1 | 1 | 0 | 0 | 0 | 0 | 1 | 1 | 0 | 0 | 0.73 | 133 | 1914 | 67 | 60 | 30 | 42 | has-miR-20b-5p | 0.205894424 | hsa-miR-204-5p | 0.025665248 | has-miR-330-3p | 0.015489557 |
| 53 | No | 73 | Male | PAF | 20.9 | 0 | 1 | 0 | 0 | 0 | 0 | 0 | 0 | 0 | 0 | 0 | 0.65 | 83 | 58 | 43 | 31 | 59 | 31 | has-miR-20b-5p | 0.038591003 | hsa-miR-204-5p | 0.000429128 | has-miR-330-3p | 0.007873557 |
| 54 | No | 70 | Male | PeAF | 23 | 1 | 2 | 0 | 1 | 0 | 0 | 0 | 1 | 1 | 0 | 0 | 0.75 | 79 | 329 | 38 | 21 | 57 | 38 | has-miR-20b-5p | 0.040721213 | hsa-miR-204-5p | 0.002955332 | has-miR-330-3p | 0.011909129 |
| 55 | Yes | 74 | Male | PeAF | 22.5 | 2 | 3 | 1 | 0 | 1 | 0 | 0 | 0 | 0 | 1 | 0 | 0.68 | 88 | 496 | 46 | 29 | 56 | 41 | has-miR-20b-5p | 0.052470172 | hsa-miR-204-5p | 0.003553792 | has-miR-330-3p | 0.008213431 |
| 56 | Yes | 76 | Female | PAF | 28.5 | 1 | 3 | 0 | 0 | 0 | 0 | 0 | 0 | 0 | 0 | 0 | 0.65 | 86 | 134 | 44 | 31 | 60 | 36 | has-miR-20b-5p | 0.134188722 | hsa-miR-204-5p | 0.002971316 | has-miR-330-3p | 0.015914298 |
| 57 | No | 75 | Female | PAF | 33.9 | 6 | 8 | 1 | 1 | 1 | 1 | 0 | 0 | 1 | 1 | 0 | 2.07 | 26 | 101 | 48 | 27 | 61 | 46 | has-miR-20b-5p | 0.020875183 | hsa-miR-204-5p | 0.008147821 | has-miR-330-3p | 0.004303817 |
| 58 | No | 61 | Male | PAF | 25.6 | 1 | 1 | 0 | 1 | 0 | 0 | 0 | 0 | 1 | 0 | 1 | 0.76 | 103 | 33 | 47 | 32 | 59 | 36 | has-miR-20b-5p | 0.087066467 | hsa-miR-204-5p | 0.012706945 | has-miR-330-3p | 0.014571774 |
| 59 | No | 66 | Male | PAF | 25.5 | 1 | 2 | 0 | 1 | 0 | 0 | 0 | 0 | 1 | 0 | 0 | 0.75 | 86 | 149 | 50 | 29 | 57 | 42 | has-miR-20b-5p | 0.061450931 | hsa-miR-204-5p | 0.002304677 | has-miR-330-3p | 0.003148525 |
| 60 | No | 79 | Female | PAF | 33.6 | 2 | 4 | 0 | 1 | 0 | 0 | 0 | 0 | 1 | 1 | 1 | 0.79 | 73 | 57 | 53 | 36 | 66 | 35 | has-miR-20b-5p | 0.080277009 | hsa-miR-204-5p | 0.010653586 | has-miR-330-3p | 0.004688735 |
| 61 | No | 55 | Male | PAF | 19.3 | 1 | 1 | 0 | 1 | 0 | 0 | 0 | 1 | 0 | 0 | 1 | 0.87 | 81 | 28 | 41 | 21 | 60 | 30 | has-miR-20b-5p | 0.069932198 | hsa-miR-204-5p | 0.005435121 | has-miR-330-3p | 0.013337703 |
| 62 | No | 76 | Female | PAF | 25.6 | 4 | 6 | 1 | 1 | 1 | 0 | 0 | 1 | 1 | 1 | 0 | 0.92 | 52 | 121 | 36 | 29 | 66 | 40 | has-miR-20b-5p | 0.042638170 | hsa-miR-204-5p | 0.000823639 | has-miR-330-3p | 0.003109272 |
| 63 | No | 46 | Male | PAF | 24.8 | 0 | 0 | 0 | 0 | 0 | 0 | 0 | 0 | 0 | 0 | 1 | 0.65 | 144 | 26 | 47 | 31 | 59 | 33 | has-miR-20b-5p | 0.063553307 | hsa-miR-204-5p | 0.007417573 | has-miR-330-3p | 0.004594773 |
| 64 | No | 54 | Male | PAF | 32.9 | 1 | 1 | 0 | 1 | 0 | 0 | 0 | 1 | 0 | 0 | 0 | 1.00 | 124 | 65 | 52 | 35 | 59 | 41 | has-miR-20b-5p | 0.118332068 | hsa-miR-204-5p | 0.014105924 | has-miR-330-3p | 0.015438442 |
| 65 | No | 65 | Male | PAF | 27.9 | 0 | 1 | 0 | 0 | 0 | 0 | 0 | 0 | 0 | 0 | 1 | 0.73 | 116 | 17 | 40 | 25 | 63 | 34 | has-miR-20b-5p | 0.074497630 | hsa-miR-204-5p | 0.004464118 | has-miR-330-3p | 0.026221390 |
| 66 | Yes | 67 | Male | PeAF | 31.3 | 2 | 3 | 1 | 1 | 0 | 0 | 0 | 1 | 0 | 1 | 0 | 2.05 | 46 | 6720 | 56 | 48 | 34 | 47 | has-miR-20b-5p | 0.017811889 | hsa-miR-204-5p | 0.005848332 | has-miR-330-3p | 0.007391996 |

| | | | | | | | | | | | | | | | | | | | | | | | | | | | | | |
|---|---|---|---|---|---|---|---|---|---|---|---|---|---|---|---|---|---|---|---|---|---|---|---|---|---|---|---|---|---|
| 67 | No | 68 | Female | PeAF | 24.9 | 0 | 2 | 0 | 0 | 0 | 0 | 0 | 0 | 0 | 0 | 0 | 0.48 | 121 | 719 | 46 | 30 | 60 | 40 | has-miR-20b-5p | 0.049330339 | hsa-miR-204-5p | 0.010383085 | has-miR-330-3p | 0.007609048 |
| 68 | No | 53 | Female | PAF | 24 | 0 | 1 | 0 | 0 | 0 | 0 | 0 | 0 | 0 | 0 | 0 | 0.74 | 86 | 22 | 44 | 31 | 60 | 33 | has-miR-20b-5p | 0.047209750 | hsa-miR-204-5p | 0.004302611 | has-miR-330-3p | 0.005542562 |
| 69 | No | 69 | Female | PAF | 21.1 | 1 | 3 | 0 | 1 | 0 | 0 | 0 | 0 | 0 | 0 | 0 | 0.50 | 87 | 64 | 48 | 33 | 59 | 38 | has-miR-20b-5p | 0.095049268 | hsa-miR-204-5p | 0.004326944 | has-miR-330-3p | 0.017360344 |
| 70 | No | 66 | Male | PeAF | 25.4 | 2 | 3 | 0 | 1 | 1 | 0 | 0 | 0 | 1 | 1 | 0 | 0.87 | 88 | 689 | 47 | 30 | 55 | 49 | has-miR-20b-5p | 0.109727446 | hsa-miR-204-5p | 0.005487543 | has-miR-330-3p | 0.005317845 |
| 71 | No | 67 | Male | PAF | 18 | 0 | 1 | 0 | 0 | 0 | 0 | 0 | 0 | 0 | 0 | 1 | 0.89 | 60 | 51 | 44 | 27 | 61 | 32 | has-miR-20b-5p | 0.095970046 | hsa-miR-204-5p | 0.006115253 | has-miR-330-3p | 0.006153514 |
| 72 | No | 44 | Male | PAF | 27.5 | 0 | 0 | 0 | 0 | 0 | 0 | 0 | 0 | 0 | 0 | 0 | 0.81 | 142 | 12 | 50 | 31 | 65 | 38 | has-miR-20b-5p | 0.041981912 | hsa-miR-204-5p | 0.005855337 | has-miR-330-3p | 0.013727931 |
| 73 | No | 55 | Male | PeAF | 23.2 | 0 | 0 | 0 | 0 | 0 | 0 | 0 | 0 | 0 | 0 | 0 | 0.93 | 83 | 393 | 48 | 35 | 65 | 31 | has-miR-20b-5p | 0.063857269 | hsa-miR-204-5p | 0.002315986 | has-miR-330-3p | 0.005348812 |
| 74 | No | 61 | Male | PAF | 20.7 | 1 | 1 | 0 | 0 | 1 | 0 | 0 | 0 | 0 | 0 | 0 | 0.71 | 91 | 108 | 49 | 38 | 62 | 35 | has-miR-20b-5p | 0.222409348 | hsa-miR-204-5p | 0.001181155 | has-miR-330-3p | 0.065230975 |
| 75 | No | 80 | Male | PeAF | 17.8 | 3 | 4 | 1 | 0 | 1 | 0 | 0 | 1 | 0 | 1 | 0 | 0.87 | 48 | 982 | 43 | 34 | 40 | 33 | has-miR-20b-5p | 0.022013627 | hsa-miR-204-5p | 0.000506942 | has-miR-330-3p | 0.002288025 |
| 76 | No | 64 | Female | PAF | 20.6 | 0 | 1 | 0 | 0 | 0 | 0 | 0 | 0 | 0 | 0 | 0 | 0.59 | 78 | 1036 | 41 | 26 | 60 | 39 | has-miR-20b-5p | 0.018212461 | hsa-miR-204-5p | 0.005479572 | has-miR-330-3p | 0.029676985 |
| 77 | Yes | 48 | Male | PAF | 26.4 | 1 | 1 | 1 | 0 | 0 | 0 | 0 | 1 | 0 | 1 | 0 | 0.68 | 147 | 270 | 41 | 26 | 62 | 35 | has-miR-20b-5p | 0.027464839 | hsa-miR-204-5p | 0.002600239 | has-miR-330-3p | 0.000997385 |
| 78 | No | 64 | Male | PeAF | 21.2 | 0 | 0 | 0 | 0 | 0 | 0 | 0 | 0 | 0 | 0 | 0 | 0.57 | 113 | 414 | 43 | 30 | 47 | 35 | has-miR-20b-5p | 0.079592594 | hsa-miR-204-5p | 0.004084434 | has-miR-330-3p | 0.023712359 |
| 79 | No | 62 | Male | PAF | 23 | 1 | 1 | 0 | 1 | 0 | 0 | 0 | 0 | 1 | 0 | 0 | 0.72 | 98 | 43 | 46 | 28 | 64 | 36 | has-miR-20b-5p | 0.084088831 | hsa-miR-204-5p | 0.003701932 | has-miR-330-3p | 0.005862800 |
| 80 | No | 74 | Male | PeAF | 29.5 | 1 | 2 | 1 | 0 | 0 | 0 | 0 | 1 | 1 | 1 | 0 | 0.91 | 87 | 783 | 53 | 35 | 64 | 44 | has-miR-20b-5p | 0.047005422 | hsa-miR-204-5p | 0.002746949 | has-miR-330-3p | 0.013493371 |
| 81 | No | 80 | Male | PAF | 22.9 | 5 | 6 | 0 | 1 | 1 | 1 | 0 | 0 | 1 | 0 | 0 | 1.55 | 38 | 131 | 52 | 36 | 60 | 46 | has-miR-20b-5p | 0.077590903 | hsa-miR-204-5p | 0.001254215 | has-miR-330-3p | 0.014535636 |
| 82 | No | 37 | Male | PAF | 17.4 | 0 | 0 | 0 | 0 | 0 | 0 | 0 | 0 | 0 | 0 | 0 | 0.66 | 106 | 61 | 48 | 31 | 63 | 28 | has-miR-20b-5p | 0.252912924 | hsa-miR-204-5p | 0.038233074 | has-miR-330-3p | 0.032607109 |
| 83 | No | 75 | Male | PAF | 23.4 | 3 | 4 | 0 | 1 | 1 | 0 | 0 | 1 | 1 | 0 | 1 | 1.58 | 37 | 139 | 52 | 35 | 59 | 41 | has-miR-20b-5p | 0.007112665 | hsa-miR-204-5p | 0.000612319 | has-miR-330-3p | 0.001735450 |
| 84 | No | 70 | Male | PAF | 29.2 | 1 | 2 | 0 | 1 | 0 | 0 | 0 | 1 | 1 | 0 | 0 | 0.50 | 151 | 63 | 51 | 33 | 60 | 45 | has-miR-20b-5p | 0.174883247 | hsa-miR-204-5p | 0.011496576 | has-miR-330-3p | 0.009050710 |
| 85 | No | 65 | Female | PAF | 20 | 1 | 2 | 0 | 1 | 0 | 0 | 0 | 1 | 1 | 0 | 0 | 0.63 | 77 | 107 | 41 | 19 | 68 | 30 | has-miR-20b-5p | 0.143165952 | hsa-miR-204-5p | 0.002512045 | has-miR-330-3p | 0.054450571 |
| 86 | Yes | 81 | Male | PAF | 22.6 | 2 | 3 | 0 | 1 | 0 | 0 | 0 | 0 | 1 | 0 | 0 | 1.10 | 48 | 211 | 51 | 40 | 36 | 44 | has-miR-20b-5p | 0.016646034 | hsa-miR-204-5p | 0.037070464 | has-miR-330-3p | 0.003870383 |
| 87 | No | 71 | Female | PeAF | 27 | 2 | 4 | 0 | 1 | 1 | 0 | 0 | 1 | 1 | 0 | 0 | 0.88 | 61 | 752 | 42 | 29 | 58 | 36 | has-miR-20b-5p | 0.046807202 | hsa-miR-204-5p | 0.008020561 | has-miR-330-3p | 0.014571774 |
| 88 | No | 67 | Female | PeAF | 18.5 | 0 | 2 | 0 | 0 | 0 | 0 | 0 | 0 | 0 | 0 | 0 | 0.89 | 42 | 594 | 37 | 25 | 66 | 30 | has-miR-20b-5p | 0.136749425 | hsa-miR-204-5p | 0.009179968 | has-miR-330-3p | 0.052715962 |
| 89 | No | 56 | Male | PeAF | 24.6 | 0 | 0 | 0 | 0 | 0 | 0 | 0 | 0 | 0 | 0 | 0 | 0.92 | 90 | 897 | 46 | 29 | 57 | 45 | has-miR-20b-5p | 0.022695535 | hsa-miR-204-5p | 0.014305889 | has-miR-330-3p | 0.001599694 |
| 90 | No | 75 | Female | PAF | 18.1 | 1 | 3 | 0 | 0 | 0 | 0 | 0 | 0 | 0 | 0 | 0 | 0.49 | 64 | 350 | 37 | 25 | 64 | 24 | has-miR-20b-5p | 0.057744739 | hsa-miR-204-5p | Undetermined | has-miR-330-3p | 0.008573092 |
| 91 | No | 72 | Male | PeAF | 26.1 | 1 | 2 | 1 | 0 | 0 | 0 | 0 | 1 | 0 | 0 | 1 | 1.07 | 59 | 704 | 53 | 32 | 58 | 48 | has-miR-20b-5p | 0.031733680 | hsa-miR-204-5p | 0.014204531 | has-miR-330-3p | 0.013361298 |
| 92 | No | 37 | Male | PAF | 37.9 | 1 | 1 | 0 | 0 | 1 | 0 | 0 | 0 | 0 | 0 | 0 | 0.72 | 213 | 36 | 52 | 30 | 59 | 38 | has-miR-20b-5p | 0.221526931 | hsa-miR-204-5p | 0.056553495 | has-miR-330-3p | 0.033646253 |
| 93 | No | 69 | Female | PAF | 32 | 0 | 2 | 0 | 0 | 0 | 0 | 0 | 0 | 0 | 0 | 1 | 0.73 | 86 | 278 | 46 | 25 | 66 | 43 | has-miR-20b-5p | 0.028240010 | hsa-miR-204-5p | 0.021000532 | has-miR-330-3p | 0.050412617 |
| 94 | No | 71 | Male | PeAF | 19.3 | 0 | 1 | 0 | 0 | 0 | 0 | 0 | 0 | 0 | 1 | 0 | 0.60 | 92 | 594 | 44 | 29 | 61 | 36 | has-miR-20b-5p | 0.190651616 | hsa-miR-204-5p | 0.014305889 | has-miR-330-3p | 0.019769799 |
| 95 | No | 74 | Male | PeAF | 25.1 | 0 | 1 | 0 | 0 | 0 | 0 | 0 | 1 | 0 | 0 | 0 | 0.86 | 82 | 91 | 42 | 22 | 66 | 40 | has-miR-20b-5p | 0.155546985 | hsa-miR-204-5p | 0.053964221 | has-miR-330-3p | 0.007977765 |
| 96 | No | 69 | Male | PAF | 19.4 | 1 | 2 | 0 | 1 | 0 | 0 | 0 | 0 | 1 | 0 | 0 | 0.95 | 60 | 130 | 47 | 29 | 60 | 30 | has-miR-20b-5p | 0.109899318 | hsa-miR-204-5p | 0.013382131 | has-miR-330-3p | 0.079907810 |
| 97 | No | 70 | Male | PAF | 21 | 1 | 2 | 0 | 1 | 0 | 0 | 0 | 0 | 1 | 0 | 0 | 0.81 | 75 | 1190 | 50 | 32 | 61 | 43 | has-miR-20b-5p | 0.022469731 | hsa-miR-204-5p | 0.014582221 | has-miR-330-3p | 0.013634152 |
| 98 | No | 64 | Male | PeAF | 30.2 | 2 | 2 | 0 | 1 | 1 | 0 | 0 | 1 | 1 | 0 | 0 | 1.12 | 75 | 584 | 50 | 30 | 58 | 42 | has-miR-20b-5p | 0.023247376 | hsa-miR-204-5p | 0.007209612 | has-miR-330-3p | 0.005844890 |
| 99 | No | 59 | Male | PeAF | 34 | 2 | 2 | 1 | 1 | 0 | 0 | 0 | 1 | 1 | 1 | 0 | 0.96 | 122 | 731 | 67 | 61 | 33 | 49 | has-miR-20b-5p | 0.006610996 | hsa-miR-204-5p | 0.003678155 | has-miR-330-3p | 0.001708662 |
| 100 | No | 63 | Male | PAF | 25.4 | 0 | 0 | 0 | 0 | 0 | 0 | 0 | 1 | 0 | 0 | 0 | 1.13 | 69 | 72 | 51 | 21 | 61 | 37 | has-miR-20b-5p | 0.056970979 | hsa-miR-204-5p | 0.015006770 | has-miR-330-3p | 0.037703590 |
| 101 | No | 82 | Female | PAF | 22.5 | 3 | 5 | 0 | 0 | 0 | 1 | 0 | 0 | 0 | 0 | 0 | 0.65 | 51 | 799 | 43 | 27 | 68 | 34 | has-miR-20b-5p | 0.042341739 | hsa-miR-204-5p | 0.010162512 | has-miR-330-3p | 0.083148862 |
| 102 | No | 83 | Female | PeAF | 17.4 | 3 | 5 | 1 | 1 | 0 | 0 | 0 | 1 | 1 | 1 | 0 | 1.04 | 24 | 6016 | 30 | 21 | 61 | 39 | has-miR-20b-5p | 0.041120729 | hsa-miR-204-5p | 0.091077531 | has-miR-330-3p | 0.043422663 |
| 103 | No | 76 | Female | PAF | 20.3 | 1 | 3 | 0 | 0 | 0 | 0 | 0 | 0 | 0 | 0 | 0 | 1.13 | 27 | 918 | 44 | 29 | 61 | 36 | has-miR-20b-5p | 0.010204413 | hsa-miR-204-5p | 0.007622136 | has-miR-330-3p | 0.023429896 |
| 104 | No | 70 | Male | PAF | 24.3 | 0 | 1 | 0 | 0 | 0 | 0 | 0 | 0 | 0 | 0 | 0 | 0.87 | 79 | 52 | 40 | 24 | 62 | 27 | has-miR-20b-5p | 0.046114971 | hsa-miR-204-5p | 0.009888867 | has-miR-330-3p | 0.043762855 |
| 105 | No | 58 | Male | PAF | 27.4 | 2 | 2 | 1 | 1 | 0 | 0 | 0 | 1 | 1 | 0 | 0 | 0.83 | 115 | 145 | 47 | 36 | 60 | 39 | has-miR-20b-5p | 0.066619608 | hsa-miR-204-5p | 0.006450077 | has-miR-330-3p | 0.009140660 |
| 106 | No | 82 | Male | PAF | 22.6 | 2 | 3 | 0 | 1 | 0 | 0 | 0 | 0 | 1 | 0 | 0 | 0.57 | 93 | 41 | 45 | 23 | 63 | 36 | has-miR-20b-5p | 0.046897516 | hsa-miR-204-5p | 0.036806133 | has-miR-330-3p | 0.056807951 |
| 107 | No | 70 | Male | PeAF | 21.6 | 2 | 3 | 1 | 1 | 0 | 0 | 0 | 0 | 0 | 0 | 0 | 0.68 | 92 | 556 | 50 | 37 | 55 | 44 | has-miR-20b-5p | 0.098600311 | hsa-miR-204-5p | 0.005026114 | has-miR-330-3p | 0.021982410 |
| 108 | No | 73 | Male | PAF | 22.1 | 2 | 3 | 0 | 1 | 1 | 0 | 0 | 1 | 0 | 0 | 1 | 0.60 | 103 | 333 | 50 | 33 | 59 | 41 | has-miR-20b-5p | 0.097135918 | hsa-miR-204-5p | 0.036578279 | has-miR-330-3p | 0.033088354 |
| 109 | No | 66 | Male | PAF | 22.5 | 0 | 1 | 0 | 0 | 0 | 0 | 0 | 0 | 0 | 0 | 0 | 0.69 | 97 | 27 | 50 | 31 | 62 | 39 | has-miR-20b-5p | 0.022939096 | hsa-miR-204-5p | 0.011782061 | has-miR-330-3p | 0.020245447 |
| 110 | No | 64 | Male | PeAF | 27 | 2 | 2 | 1 | 1 | 0 | 0 | 0 | 1 | 1 | 1 | 0 | 0.74 | 113 | 134 | 55 | 38 | 42 | 44 | has-miR-20b-5p | 0.030730889 | hsa-miR-204-5p | 0.107820643 | has-miR-330-3p | 0.021142894 |
| 111 | No | 69 | Male | PeAF | 24.5 | 2 | 3 | 1 | 1 | 0 | 0 | 0 | 1 | 1 | 1 | 0 | 1.37 | 53 | 2518 | 61 | 53 | 32 | 45 | has-miR-20b-5p | 0.018289065 | hsa-miR-204-5p | 0.001027065 | has-miR-330-3p | 0.008996815 |
| 112 | No | 50 | Male | PAF | 27.5 | 1 | 1 | 0 | 1 | 0 | 0 | 0 | 0 | 0 | 0 | 0 | 0.89 | 112 | 6 | 54 | 28 | 61 | 41 | has-miR-20b-5p | 0.025919823 | hsa-miR-204-5p | 0.016805881 | has-miR-330-3p | 0.008349115 |
| 113 | No | 49 | Male | PAF | 24.2 | 0 | 0 | 0 | 0 | 0 | 0 | 0 | 0 | 0 | 0 | 0 | 0.88 | 103 | 25 | 50 | 34 | 66 | 29 | has-miR-20b-5p | 0.247137127 | hsa-miR-204-5p | 0.001378206 | has-miR-330-3p | 0.025430213 |
| 114 | No | 54 | Male | PAF | 22.1 | 2 | 2 | 1 | 1 | 0 | 0 | 0 | 1 | 0 | 1 | 0 | 0.63 | 135 | 1177 | 50 | 40 | 60 | 40 | has-miR-20b-5p | 0.028489471 | hsa-miR-204-5p | 0.005210234 | has-miR-330-3p | 0.013480069 |
| 115 | No | 65 | Male | PAF | 22.9 | 0 | 1 | 0 | 0 | 0 | 0 | 0 | 1 | 0 | 0 | 1 | 0.83 | 84 | 63 | 49 | 34 | 61 | 31 | has-miR-20b-5p | 0.023360171 | hsa-miR-204-5p | 0.001087452 | has-miR-330-3p | 0.004848201 |
| 116 | No | 70 | Male | PAF | 28.1 | 1 | 2 | 0 | 1 | 0 | 0 | 0 | 1 | 0 | 0 | 0 | 0.84 | 96 | 42 | 55 | 33 | 57 | 41 | has-miR-20b-5p | 0.045723257 | hsa-miR-204-5p | 0.005420381 | has-miR-330-3p | 0.025584402 |
| 117 | No | 76 | Male | PAF | 25 | 4 | 5 | 0 | 1 | 0 | 1 | 0 | 1 | 1 | 0 | 0 | 0.95 | 64 | 337 | 50 | 33 | 58 | 39 | has-miR-20b-5p | 0.129928365 | hsa-miR-204-5p | 0.012882061 | has-miR-330-3p | 0.044207511 |
| 118 | No | 74 | Male | PAF | 25.7 | 2 | 3 | 0 | 1 | 1 | 0 | 0 | 0 | 0 | 0 | 0 | 0.85 | 76 | 113 | 48 | 30 | 59 | 37 | has-miR-20b-5p | 0.049578875 | hsa-miR-204-5p | 0.006993745 | has-miR-330-3p | 0.005491952 |
| 119 | No | 56 | Male | PAF | 21 | 0 | 0 | 0 | 0 | 0 | 0 | 0 | 0 | 0 | 0 | 0 | 0.81 | 84 | 32 | 43 | 33 | 60 | 36 | has-miR-20b-5p | 0.085611772 | hsa-miR-204-5p | 0.022665487 | has-miR-330-3p | 0.021817254 |
| 120 | No | 78 | Male | PeAF | 24.3 | 3 | 4 | 1 | 1 | 0 | 0 | 0 | 1 | 1 | 1 | 0 | 0.91 | 56 | 926 | 50 | 37 | 42 | 43 | has-miR-20b-5p | 0.028250101 | hsa-miR-204-5p | 0.009443347 | has-miR-330-3p | 0.003409442 |
| 121 | No | 57 | Male | PAF | 23.8 | 1 | 1 | 0 | 1 | 0 | 0 | 0 | 0 | 0 | 0 | 0 | 1.10 | 81 | 45 | 44 | 30 | 61 | 35 | has-miR-20b-5p | 0.134431021 | hsa-miR-204-5p | 0.027976940 | has-miR-330-3p | 0.030632395 |
| 122 | No | 57 | Male | PeAF | 22.8 | 1 | 1 | 1 | 0 | 0 | 0 | 0 | 0 | 0 | 0 | 0 | 0.88 | 95 | 431 | 45 | 31 | 62 | 36 | has-miR-20b-5p | 0.095573447 | hsa-miR-204-5p | 0.025796247 | has-miR-330-3p | 0.019769799 |
| 123 | No | 69 | Male | PAF | 22.9 | 0 | 1 | 0 | 0 | 0 | 0 | 0 | 1 | 0 | 0 | 1 | 1.03 | 59 | 76 | 42 | 25 | 61 | 36 | has-miR-20b-5p | 0.070612839 | hsa-miR-204-5p | 0.021177807 | has-miR-330-3p | 0.006488735 |
| 124 | No | 70 | Female | PAF | 20 | 0 | 2 | 0 | 0 | 0 | 0 | 0 | 0 | 0 | 0 | 1 | 0.54 | 73 | 148 | 48 | 34 | 59 | 39 | has-miR-20b-5p | 0.111466596 | hsa-miR-204-5p | 0.062909382 | has-miR-330-3p | 0.021712154 |
| 125 | No | 68 | Male | PAF | 21.7 | 0 | 1 | 0 | 0 | 0 | 0 | 0 | 1 | 0 | 0 | 0 | 0.87 | 75 | 37 | 52 | 29 | 64 | 37 | has-miR-20b-5p | 0.153177220 | hsa-miR-204-5p | 0.006278941 | has-miR-330-3p | 0.037953068 |
| 126 | No | 50 | Male | PeAF | 26.3 | 0 | 0 | 0 | 0 | 0 | 0 | 0 | 0 | 0 | 0 | 0 | 0.75 | 129 | 87 | 48 | 31 | 57 | 34 | has-miR-20b-5p | 0.199261330 | hsa-miR-204-5p | 0.060490987 | has-miR-330-3p | 0.026911110 |
| 127 | No | 71 | Female | PAF | 20.3 | 0 | 2 | 0 | 0 | 0 | 0 | 0 | 0 | 0 | 0 | 0 | 0.49 | 84 | 87 | 39 | 22 | 57 | 35 | has-miR-20b-5p | 0.147556359 | hsa-miR-204-5p | 0.003276572 | has-miR-330-3p | 0.023632170 |
| 128 | No | 76 | Male | PAF | 24.8 | 2 | 3 | 0 | 1 | 0 | 0 | 0 | 1 | 0 | 0 | 0 | 0.81 | 77 | 47 | 45 | 29 | 57 | 38 | has-miR-20b-5p | 0.027364263 | hsa-miR-204-5p | 0.005259681 | has-miR-330-3p | 0.006395662 |
| 129 | No | 34 | Male | PeAF | 26.1 | 0 | 0 | 0 | 0 | 0 | 0 | 0 | 1 | 0 | 0 | 0 | 0.95 | 125 | 342 | 51 | 42 | 51 | 38 | has-miR-20b-5p | 0.027051305 | hsa-miR-204-5p | 0.045436106 | has-miR-330-3p | 0.058321703 |
| 130 | Yes | 64 | Female | PAF | 28 | 1 | 2 | 0 | 1 | 0 | 0 | 0 | 0 | 0 | 0 | 0 | 0.60 | 123 | 78 | 42 | 25 | 61 | 31 | has-miR-20b-5p | 0.050276360 | hsa-miR-204-5p | 0.027037883 | has-miR-330-3p | 0.031309414 |
| 131 | Yes | 60 | Female | PAF | 28 | 1 | 2 | 0 | 0 | 1 | 0 | 0 | 0 | 0 | 0 | 0 | 0.39 | 160 | 28 | 44 | 25 | 61 | 34 | has-miR-20b-5p | 0.010178455 | hsa-miR-204-5p | 0.005960454 | has-miR-330-3p | 0.010065684 |
| 132 | Yes | 68 | Male | PeAF | 28.3 | 1 | 2 | 1 | 0 | 0 | 0 | 0 | 1 | 1 | 1 | 1 | 0.95 | 85 | 605 | 67 | 56 | 25 | 43 | has-miR-20b-5p | 0.004299687 | hsa-miR-204-5p | 0.000340821 | has-miR-330-3p | 0.002288093 |
| 133 | Yes | 71 | Male | PeAF | 21.8 | 2 | 3 | 1 | 1 | 0 | 0 | 0 | 1 | 1 | 1 | 1 | 0.89 | 70 | 816 | 49 | 38 | 51 | 49 | has-miR-20b-5p | 0.016792048 | hsa-miR-204-5p | 0.001148515 | has-miR-330-3p | 0.003747485 |
| 134 | Yes | 65 | Male | PeAF | 28 | 2 | 3 | 1 | 1 | 0 | 0 | 0 | 1 | 1 | 1 | 0 | 1.64 | 47 | 3562 | 54 | 37 | 56 | 55 | has-miR-20b-5p | 0.032792886 | hsa-miR-204-5p | 0.013676539 | has-miR-330-3p | 0.005012529 |
| 135 | Yes | 79 | Male | PeAF | 22.1 | 2 | 3 | 1 | 0 | 0 | 0 | 0 | 0 | 0 | 0 | 0 | 0.91 | 62 | 995 | 48 | 31 | 57 | 46 | has-miR-20b-5p | 0.101136944 | hsa-miR-204-5p | 0.019382362 | has-miR-330-3p | 0.002168195 |

| | | | | | | | | | | | | | | | | | | | | | | | | | | | | | |
|---|---|---|---|---|---|---|---|---|---|---|---|---|---|---|---|---|---|---|---|---|---|---|---|---|---|---|---|---|---|
| 136 | Yes | 71 | Male | PeAF | 22.1 | 1 | 2 | 0 | 1 | 0 | 0 | 0 | 1 | 1 | 0 | 0 | 0.78 | 67 | 265 | 52 | 35 | 55 | 59 | has-miR-20b-5p | 0.028751366 | hsa-miR-204-5p | 0.018039044 | has-miR-330-3p | 0.009263763 |
| 137 | Yes | 79 | Male | PeAF | 27 | 3 | 4 | 1 | 1 | 0 | 0 | 0 | 1 | 1 | 0 | 0 | 1.72 | 32 | 1806 | 44 | 26 | 62 | 40 | has-miR-20b-5p | 0.018532826 | hsa-miR-204-5p | 0.005293143 | has-miR-330-3p | 0.007386952 |
| 138 | Yes | 66 | Female | PeAF | 15 | 1 | 3 | 1 | 0 | 0 | 0 | 0 | 0 | 0 | 1 | 0 | 0.44 | 71 | 626 | 50 | 27 | 67 | 44 | has-miR-20b-5p | 0.090777401 | hsa-miR-204-5p | 0.040615494 | has-miR-330-3p | 0.016910979 |
| 139 | Yes | 77 | Male | PeAF | 24.1 | 3 | 4 | 1 | 1 | 1 | 0 | 0 | 1 | 0 | 1 | 0 | 0.78 | 76 | 1788 | 44 | 31 | 49 | 46 | has-miR-20b-5p | 0.058673316 | hsa-miR-204-5p | 0.001829874 | has-miR-330-3p | 0.019452308 |
| 140 | Yes | 48 | Female | PeAF | 18.9 | 1 | 2 | 1 | 0 | 0 | 0 | 0 | 1 | 0 | 0 | 0 | 0.72 | 71 | 760 | 42 | 25 | 65 | 46 | has-miR-20b-5p | 0.052606432 | hsa-miR-204-5p | 0.007114608 | has-miR-330-3p | 0.018858656 |
| 141 | Yes | 74 | Male | PeAF | 22.4 | 1 | 2 | 1 | 0 | 0 | 0 | 0 | 1 | 0 | 0 | 0 | 1.02 | 52 | 1509 | 53 | 50 | 25 | 40 | has-miR-20b-5p | 0.007031824 | hsa-miR-204-5p | 0.006585486 | has-miR-330-3p | 0.011760178 |
